# Ribosomal subunit protein typing using matrix-assisted laser desorption ionization time-of-flight mass spectrometry (MALDI-TOF MS) for the identification and discrimination of *Aspergillus* species

**DOI:** 10.1186/s12866-017-1009-3

**Published:** 2017-04-26

**Authors:** Sayaka Nakamura, Hiroaki Sato, Reiko Tanaka, Yoko Kusuya, Hiroki Takahashi, Takashi Yaguchi

**Affiliations:** 1 Research Institute for Sustainable Chemistry, National Institute of Advanced Industrial Science and Technology (AIST), Higashi 1-1-1, Tsukuba, Ibaraki, 305-8565 Japan; 2 Medical Mycology Research Center, Chiba University, 1-8-1 Inohana, Chuo-ku, Chiba, 260-8673 Japan

**Keywords:** *Aspergillus*, Ribosomal subunit proteins, Matrix-assisted laser desorption ionization time-of-flight mass spectrometry

## Abstract

**Background:**

Accurate identification of *Aspergillus* species is a very important subject. Mass spectral fingerprinting using matrix-assisted laser desorption ionization time-of-flight mass spectrometry (MALDI-TOF MS) is generally employed for the rapid identification of fungal isolates. However, the results are based on simple mass spectral pattern-matching, with no peak assignment and no taxonomic input. We propose here a ribosomal subunit protein (RSP) typing technique using MALDI-TOF MS for the identification and discrimination of *Aspergillus* species. The results are concluded to be phylogenetic in that they reflect the molecular evolution of housekeeping RSPs.

**Results:**

The amino acid sequences of RSPs of genome-sequenced strains of *Aspergillus* species were first verified and compared to compile a reliable biomarker list for the identification of *Aspergillus* species. In this process, we revealed that many amino acid sequences of RSPs (about 10–60%, depending on strain) registered in the public protein databases needed to be corrected or newly added. The verified RSPs were allocated to RSP types based on their mass. Peak assignments of RSPs of each sample strain as observed by MALDI-TOF MS were then performed to set RSP type profiles, which were then further processed by means of cluster analysis. The resulting dendrogram based on RSP types showed a relatively good concordance with the tree based on β-tubulin gene sequences. RSP typing was able to further discriminate the strains belonging to *Aspergillus* section *Fumigati*.

**Conclusions:**

The RSP typing method could be applied to identify *Aspergillus* species, even for species within section *Fumigati*. The discrimination power of RSP typing appears to be comparable to conventional β-tubulin gene analysis. This method would therefore be suitable for species identification and discrimination at the strain to species level. Because RSP typing can characterize the strains within section *Fumigati*, this method has potential as a powerful and reliable tool in the field of clinical microbiology.

**Electronic supplementary material:**

The online version of this article (doi:10.1186/s12866-017-1009-3) contains supplementary material, which is available to authorized users.

## Background

*Aspergillus* is a saprophytic genus found in diverse environments [1]. Some species, typically *A. fumigatus*, are causative agents of aspergillosis [2]. Because the degree of virulence and susceptibility to antifungal agents are known to vary among species [3], accurate identification of *Aspergillus* species is a very important subject, especially in the field of clinical mycology.

The identification of fungal species has up to now been based on the morphological characteristics of colonies and filaments as observed by microscopy [4]. However, the morphology-based method suffers several drawbacks. It requires specialized skills and knowledge and is tedious and time-consuming work. Nevertheless, some strains lack obvious characteristic features under laboratory conditions. To achieve objective identification, molecular biological methods based on the DNA sequences of particular genes are increasingly being adopted. The internal transcribed spacer (ITS) regions between 18S rRNA, 5.8S rRNA, and 28S rRNA are regarded as “barcode regions” and are frequently used as biomarkers for species identification [5]. DNA sequences that code housekeeping proteins such as β-tubulin [6] and calmodulin [7] are also often used for detailed molecular studies. To improve the resolution of species discrimination, combinations of multi-genes have been attempted. For example, a combination of two genes (β-tubulin and calmodulin) [8] or four genes (β-tubulin, calmodulin, ITS and large-subunit rDNA, and RNA polymerase II) [9] has been used to characterize *A. fumigatus* strains. Multi-locus sequence typing (MLST), focusing on seven types of gene fragments, has also been applied to characterize *A. fumigatus* strains [10]. These DNA-based methods provide a more objective evaluation than the traditional morphological method.

In the field of medical microbiology, much attention has been paid recently to the mass spectrometric technique of matrix-assisted laser desorption ionization time-of-flight mass spectrometry (MALDI–TOF MS) as a tool for the rapid identification of fungal isolates [11]. MALDI–TOF MS has major clinical advantages, since it requires much smaller samples and the total process from sample preparation to data analysis is very rapid. This method is a type of mass spectral fingerprinting, for which mass spectral databases are commercially available from several mass spectrometer companies. The rapid identification and discrimination of clinical *A. fumigatus* isolates has been reported using this method [12, 13]. Several research groups have further attempted to discriminate *Aspergillus* isolates at the species and strain level [14–16]. However, Welker [17] has pointed out the following problems with this method in his review.

(i) the general finding that the proteome is very dynamic in living cells and hence protein pattern expectedly could be subject to changes in response to growth conditions,
(ii) doubts whether differences and similarities in mass spectral patterns are completely consistent with the established taxonomy,
(iii) a lack of comprehensive databases covering all clinically relevant species.

Furthermore, in the author’s opinion, the reported mass spectra of fungal samples sometimes show too few peaks when sample preparation is performed using the recommended protocol proposed by the manufacturers.

To overcome these problems relating to mass spectral fingerprinting, we have proposed a method using ribosomal proteins as biomarkers for microorganism analysis by MALDI–TOF MS [18–25]. Ribosomal proteins are typical housekeeping proteins and are abundantly present in microorganisms’ cells. Prokaryotic (bacterial) ribosomes consist of 57 kinds of ribosomal subunit proteins (RSPs), whereas eukaryotic ribosomes typically consist of 78 RSPs. The combination of subunit proteins and their structures are not influenced by culture conditions. Because most RSPs are basic proteins with higher proton affinity (i.e., easily producing [M + H]^+^ ions) and their masses are distributed in the range of ca. 4 - 30 kDa, RSPs can be easily observed in MALDI mass spectra [26]. We have reported that the identification of bacterial species and classification at the strain level can be accomplished based on the expressed mass types of RSPs [18–25]. The masses of RSPs used as biomarkers can be estimated from translational amino acid sequences of genome-sequenced strains, which can be obtained from public databases such as UniProt Knowledgebase (UniProtKB) [27]. Our proposed method is a form of molecular typing like MLST, based on bioinformatics. The biomarker RSPs are a complex of typical housekeeping proteins. Since the sequence variation of RSPs observed as the peak shift on the MALDI mass spectra results from molecular evolution, the results of identification and discrimination of microorganisms are assumed to phylogenetic ally. This is the crucial difference between our proposed RSP typing as “phylogenetic” method and the conventional mass spectral fingerprinting as “chemotaxonomic” method.

The aim of our project is to extend the RSP-based method to the identification of eukaryotic fungi. As the first step, we have investigated the actual state of information of RSPs of fungi registered in public protein databases through the characterization of ribosomal protein fractions extracted from genome-sequenced *A. fumigatus* strains as a model [28]. In our previous paper [28], we revealed that more than half of the amino acid sequences of RSPs registered in the public databases were incorrect, due chiefly to mis-annotation of exon/intron structures. We were able to successfully correct the sequence errors using a combination of *in silico* inspection by sequence homology analysis and MALDI–TOF MS measurements. Post-translational modifications such as acetylation and methylation could also be verified. In this way, the expressed masses of RSPs observed under 16,000 Da could finally be confirmed.

As the next step, this paper describes the results of comparable characterization of RSPs of eleven *Aspergillus* species to establish biomarker references for the reliable identification of *Aspergillus* species. First, verification and correction of the amino acid sequences of RSPs and confirmation of post-translational modifications common to all sample strains were performed to accurately determine the expressed mass, as described in our previous paper [28]. RSPs with appropriate intensity commonly observed in each strain were then selected as reliable biomarkers for the identification of *Aspergillus* species. The selected RSPs of each strain were categorized into “RSP types” based on their mass and used to construct a dendrogram. The resulting dendrogram was compared with that arrived at using the DNA-based method; the reliability of the species identification and discrimination of this method was then assessed.

## Results and discussion

### Characterization of RSPs of genome-sequenced *Aspergillus* strains

In our previous paper [28], the amino acid sequences of RSPs in *A. fumigatus* strains were verified to compile the reference mass list of expressed RSPs. We noted that more than half of the amino acid sequences in the public databases, such as UniProtKB [27] and the NCBI protein database [29] were incorrect. These errors could be corrected by a combination of *in silico* inspection using sequence homology analysis and verification of actual expressed masses of RSPs by MALDI–TOF MS measurements. In this study, by applying this strategy, the amino acid sequences of RSPs of ten genome-sequenced strains of *Aspergillus* species were further verified and compared to build a reliable biomarker list for the identification of *Aspergillus* species. The sample strains used in this study are summarized in Table 1. For RSPs with amino acid sequences not registered in the public databases, corresponding gene sequences were manually identified in the genome sequence by referring to gene sequences of *A. fumigatu*s strains. The genome sequence of *A. viridinutans* is not yet published, but the authors have annotated the RSP sequences manually. Finally, 26 kinds of RSPs whose molecular weights were under 16,000 Da were selected that are common to those already verified for *A. fumigatus* strains [28]. Supporting Information Additional file 3: Figures SI-1 to SI-10 show the mass spectra of each genome-sequenced strain (except for *A. fumigatus*, for which the mass spectra were reported in ref. [28]). Additional file 1: Table SI-1 summarizes accession numbers in public protein databases of ribosomal protein biomarkers of genome sequenced strains used in this study. Here, the names of RSPs are adopted from the yeast nomenclature system [30] to prevent confusion in the RSP’s nomenclature. Supporting Information Additional file 2: Table SI-2 summarizes the data of the RSPs of ten genome-sequenced strains such as the accession number, post-translational modifications, corrected amino acid sequences, and corrected exon/intron structures (the corrected sequences of *A. fumigatus* A1163 and Af293 have been reported in ref [28]).

**Table 1:** List of sample strains used in this study

species	strain names ^a)^
*A. fumigatus*	* IFM 53842 (= A1163), *IFM 54229 (= Af293), IFM 57323^NT^
*N. fischeri*	* IFM 57324^T^ (= NRRL 181^T^)
*A. lentulus*	* IFM 54703^T^, IFM 47547, IFM 58399, IFM 60648, IFM 61392, IFM 62073, IFM 62096
*A. viridinutans*	* IFM 47045^T^
*A. felis* (former *A. viridinutans*)	IFM 59564, IFM 60053, IFM 62093
*A. pseudoviridinutans* (former *A. viridinutans*)	IFM 55266, IFM 62075
*A. wyomingensis* (former *A. viridinutans*)	IFM 62083
*A. udagawae*	*IFM 46973^T^, IFM 46972, IFM 5058, IFM 51744, IFM 53868, IFM 61606, IFM 62070, IFM 62100
*A. clavatus*	* IFM 60676^NT^ (= NRRL 1^NT^)
*A. niger*	* CBS 513.88
*A. kawachii*	* IFO 4308
*A. flavus*	* IFM 60677 (= NRRL 3357)
*A. oryzae*	* IFM 59475 (=RIB 40)
*A. nidulans*	* IFM 60678 (= FGSC A4)

a) * genome sequenced strain.

Table 2 shows the number and ratio of incorrect or not registered sequences among 26 RSPs for nine newly analyzed genome-sequenced strains (the result of *A. viridinutans*, annotated by the authors, was not added to this list). Depending on the species, about 10 - 60% RSPs needed to be corrected or newly added. The source of sequence errors in prokaryotic bacteria was chiefly due to misidentification of the start codon [20]. The main reason in *Aspergillus* fungi seemed to be due to misidentification of the exon/intron structure, resulting in incorrect CDS as well as an incorrect stop codon caused by frame shift. Because this type of error is unique to prokaryotes, a similar problem in the annotations of RSP genes might have occurred in other fungi.

**Table 2:** The number of corrected RSPs and their ratio to analyzed RSPs

Species	Strains	The number of the corrected RSPs	The ratio of the corrected RSPs to the analyzed RSPs (%)
*N. fischeri*	IFM 57324^T^ (= NRRL 181^T^)	11	42
*A. lentulus*	IFM 54703^T^	14	54
*A. udagawae*	IFM 46973^T^	15	58
*A. clavatus*	IFM 60676^NT^ (= NRRL 1^NT^)	9	35
*A. niger*	CBS 513.88	4	15
*A. kawachii*	IFO 4308	11	42
*A. flavus*	IFM 60677 (= NRRL 3357)	7	27
*A. oryzae*	IFM 59475 (=RIB 40)	3	12
*A. nidulans*	IFM 60678 (= FGSC A4)	4	15

The post-translational modifications were then confirmed by referring to already-reported modifications in eukaryotic RSPs. Details of the assignments of each modification are described in the Supporting Information (Additional file 4: Figures SI-11 to SI-14). Acetylation (S16, S21, S24, S28, L31, and L35), methylation (L42), and two hydroxylations (S23) have been reported in several papers (see citations in ref. [28]) and have also observed in *A. fumigatus* [28]. A mass shift of these RSPs from the calculated sequence mass after taking into account N-terminal methionine loss was commonly observed in all sample strains (+42 Da for acetylation, +14 Da for methylation, and +32 Da for two hydroxylations). This result suggests these modifications to be evolutionarily-conserved modifications, at least in *Aspergillus* species. In addition, S27 showed a common +28 Da shift, suggesting two methylations. Although this modification has, to our knowledge, not been reported before, two methylations of S27 were concluded to be common modifications in *Aspergillus* species.

### Species identification using the RSP types

**Fig. 1:**
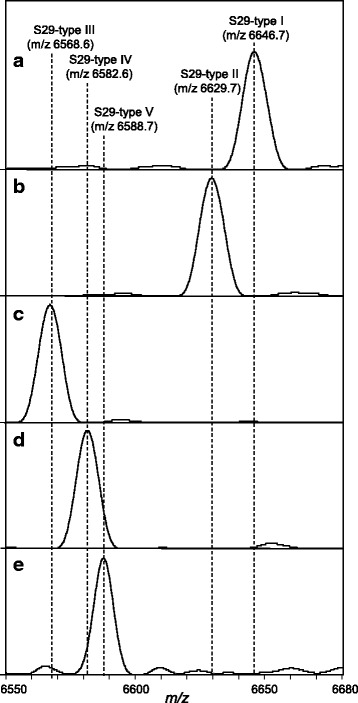
Expanded view of S29 (*m/z* 6550-6680) regions. **a** *A. fumigatus* A1163, **b** *A. clavatus* NRRL 1^NT^, (C) *A. niger* CBS 513.88, (D) *A. flavus* NRRL 3357, and (E) *A. nidulans* FGSC A4

The amino acid sequences and the theoretical mass of RSPs thus determined mostly varied among species. This finding strongly suggests that species identification can be performed using RSPs as biomarkers. To make a reference table for the RSP typing, each RSP was classified into different types based on mass. For example, S29 has five types of different expressed mass, of which the peaks are distributed approximately from *m/z* 6570 to *m/z* 6650, as shown in Fig. 1 (the whole-range mass spectra are shown in Supporting Information Additional file 3: Figures SI-1 to SI-10). For S29, Type I was first allocated to *A. fumigatus* A1163 observed at *m/z* 6647, which was common with that of *N. fischeri* NRRL 181^T^, *A. lentulus* IFM 54703^T^, *A. viridinutans* IFM 47045^T^, and *A. udagawae* IFM 46973^T^. Interestingly, these species belong to *Aspergillus* section *Fumigati*. The mass of S29 of *A. clavatus* NRRL 1^NT^ was different from Type I, so it was allocated to Type II. In the same manner, S29 of *A. niger* CBS 513.88 and *A. kawachii* IFO 4308 were allocated to Type III, that of *A. flavus* NRRL 3357 and *A. oryzae* RIB 40 to Type IV, and that of *A. nidulans* FGSC A4 to Type V. The type classification was conducted in the same way in other RSPs. Table 3 summarizes the mass and types of RSPs of each genome-sequenced strain, in which the post-translational modifications were taken into consideration as affecting the mass in this list.

**Table 3:** The expressed masses and the types of ribosomal protein biomarkers of genome-sequenced strains used in this study

RP name	*A. fumigatus* IFM 53842	*A. fumigatus* IFM 54229	*N. fischeri* IFM 57324^T^	*A. lentulus* IFM 54703^T^	*A. viridinutans* IFM 47045^T^	*A. udagawae* IFM 46973^T^
	= A1163	= Af293	= NRRL 181^T^			
L40	I (6002.3)	I	II (6016.3)	II	II	II
L39	I (6151.2)	I	I	I	I	I
S29	I (6646.7)	I	I	I	I	I
S30	I (6789.1)	I	II (6807.1)	II	III (6793.1)	II
L29	I (7456.6)	I	I	I	I	II (7470.6)
S28	I (7710.0)	I	I	I	I	I
S27	I (8765.3)	I	I	I	I	I
S31	I (9134.9)	I	I	I	I	II (9120.9)
L38	I (9153.8)	I	II (9169.8)	II	II	II
S21	I (10038.1)	II (10052.2)	I	III (10035.1)	IV (10008.1)	V (10024.1)
L43	I (10025.8)	I	I	I	I	I
L37	I (10386.9)	I	I	II (10387.9)	III (10358.9)	II
L30	I (11171.1)	I	I	I	I	I
L36	I (11869.8)	I	II (11861.8)	III (11847.8)	II	IV (11875.8)
L42	I (12028.3)	I	II (12009.2)	III (11979.2)	IV (11995.2)	V (11965.2)
L33	I (12215.1)	I	I	I	II (12233.1)	III (12206.1)
L34	I (13164.5)	I	I	I	I	I
S26	I (13338.7)	I	I	I	I	I
L31	I (13919.1)	I	II (13937.1)	II	II	III (13937.1)
L35	I (14532.0)	I	II (14518.0)	II	^a)^	III (14548.0)
L32	I (14836.6)	I	II (14822.6)	II	II	III (14880.6)
L26	I (14979.4)	I	II (14982.5)	II	II	III (14954.4)
S24	I (15226.6)	I	I	I	II (15212.6)	I
L27	I (15682.6)	I	II (15654.5)	II	II	III (15667.5)
S23	I (15802.5)	I	II (15786.5)	II	II	II
S16	I(15883.4)	I	II (15853.4)	II	II	II
RP name	*A. clavatus* IFM 60676^NT^	*A. niger* CBS 513.88	*A. kawachii* IFO 4308	*A. flavus* NRRL 3357	*A. oryzae* RIB 40	*A. nidulans* FGSC A4
	= NRRL 1^NT^					
L40	II	II	II	III (6043.3)	III	IV (6057.3)
L39	I	II(6123.2)	II	I	I	III (6123.2)
S29	II (6629.7)	III	III (6568.6)	IV (6582.6)	IV	V (6588.7)
S30	IV (6790.0)	V (6745.0)	VI (6729.0)	VII (6691.9)	VII	VIII (6761.1)
L29	III (7433.6)	IV (7368.4)	IV	V (7414.5)	V	VI (7387.5)
S28	I	II (7681.9)	II	III (7708.0)	III	IV (7754.0)
S27	I	II (8735.3)	II	III (8792.4)	III	IV (8807.3)
S31	II	III (9101.8)	III	IV (9090.8)	IV	V (9111.9)
L38	III (9160.8)	IV (9073.7)	IV	V (9173.9)	V	VI (9128.8)
S21	VI (9922.9)	VII (10027.1)	VIII (10041.2)	IX (10050.1)	IX	X (10064.1)
L43	II (10011.8)	IV (9997.7)	IV	IV	IV	V (10011.8)
L37	IV (10279.7)	V (10451.9)	VI (10435.9)	VII (10436.9)	VII	VIII (10371.8)
L30	II (11169.1)	III (11210.1)	III	IV (11223.1)	IV	V (11259.2)
L36	V (11850.8)	VI (11831.8)	VI	III	III	VII (11757.7)
L42	VI (11921.1)	VII (12025.2)	VII	VIII (12029.2)	VIII	IX (11994.2)
L33	II	IV (12250.1)	IV	V (12299.2)	V	VI (12216.1)
L34	I	II (13147.4)	II	III (13178.5)	III	IV (13133.4)
S26	I	II (13381.7)	II	II	II	IV (13349.7)
L31	IV (13809.9)	V (13878.0)	V	VI (13981.2)	VI	VII (13847.9)
L35	IV (14517.0)	V (14384.8)	VI (14411.9)	VII (14536.9)	VII	VIII (14458.0)
L32	IV (14852.6)	V (14891.6)	V	VI (14794.5)	VI	VII (14838.5)
L26	IV (14915.3)	V (14877.2)	VI (14891.2)	VII (14999.3)	VII	VIII (15084.6)
S24	III (15130.5)	IV (15163.5)	IV	V (15157.5)	V	VI (15232.7)
L27	IV (15636.5)	V (15610.4)	V	VI (15613.4)	VI	VII (15642.5)
S23	II	III (15739.4)	III	IV (15752.4)	IV	V (15757.5)
S16	III (15849.4)	IV (15881.4)	IV	V (15835.3)	V	VI (15852.3)

^a)^ The amino acid sequence of L35 in *A. viridinutans* IFM 47045^T^ was not obtained from the draft genome sequences

**Fig. 2:**
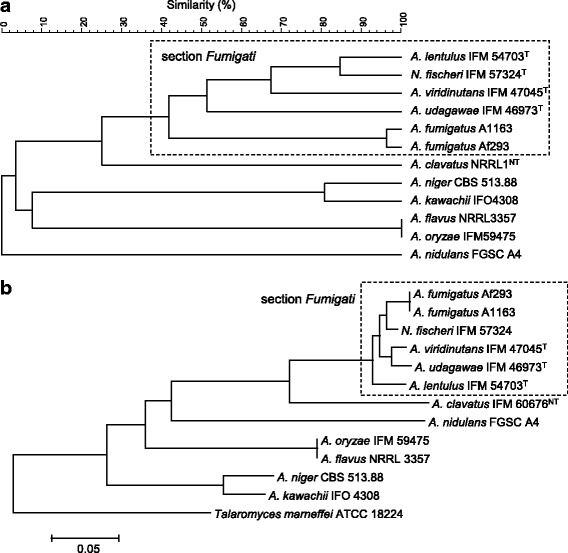
Dendrograms constructed based on RSP typing (**a**) and the DNA sequence of β-tubulin (**b**)

The distribution of the RSP types shown in Table 3 was then processed using the unweighted pair group method with arithmetic mean (UPGMA) cluster analysis using a categorical coefficient. Fig. 2 compares the dendrogram based on the RSP types (Fig. 2a) with that based on the β-tubulin gene sequence (Fig. 2b). Among the *Aspergillus* species used in this study, *A. fumigatus*, *N. fischeri*, *A. lentulus*, *A. viridinutans*, and *A. udagawae* are known to be genetically related species, belonging to section *Fumigati* [31] (note that *Neosartorya* is a teleomorph of *Aspergillus*). Interestingly, these five species form a cluster in the dendrograms based on both the RSP type (Fig. 2a) and the β-tubulin gene sequence (Fig. 2b). In these sample strains, eight RSPs (L30, L34, L39, L43, S26, S27, S28, and S29) of the analyzed 26 RSPs (31%) matched completely.

*Aspergillus clavatus* has been suggested to be closest to section *Fumigati* as shown in the dendrogram based on β-tubulin (Fig. 2b). RSP typing (Fig. 2a) also shows the closest position of *A. clavatus* next to section *Fumigati*. Five RSPs (S26, S27, S28, L34, and L39) of common eight RSPs in section *Fumigati* are also shared with *A. clavatus*.

The positions of *A. niger*, *A. kawachii*, *A. flavus*, *A. oryzae*, and *A. nidulans* are distinct from the cluster of section *Fumigati*. The RSP types of these species show little in common with section *Fumigati* species: only L39 of *A. oryzae* and *A. flavus* was common. Of these species, pairs of ‘*A. niger* and *A. kawachii*’ and ‘*A. flavus* and *A. oryzae*’ are known to be very closely interrelated: *A. flavus* and *A. oryzae* are assumed to be ecotypes of the same species because they have only 350 unique genes, even when comparing the total genome sequences [32]. In the types of 26 RSPs used in this study, 21 out of 26 RSPs agreed between *A. niger* and *A. kawachii* and also 100% of RSPs agreed between *A. flavus* and *A. oryzae*. The ratio of conformity between species thus appears to be reflected in the relatedness between species.

As discussed above, the dendrogram based on the RSP types shows relatively good concordance with the tree based on β-tubulin gene sequences (Fig. 2b) and with the tree arrived at by genomic analysis of *Aspergillus* fungi [1]. This result suggests that RSP typing can perform accurate species identification that reflects molecular evolution. Therefore, the dendrogram constructed by the RSP typing can be considered as a kind of phylogenetic tree.

### The effectiveness of RSP typing for discriminating section *Fumigati* strains

Because sensitivity to antifungal agents differs between *A. fumigatus* and other species within section *Fumigati*, accurate discrimination of these strains is very important [31]. However, traditional morphological analysis does not always accurately locate species within section *Fumigati*, so misidentifications often occur. To overcome this problem, Yaguchi *et al*. have characterized the species within section *Fumigati* by molecular phylogenetic analysis using multiple genes [33]. On the other hand, as described in the previous section, RSP typing shows the potential of species discrimination within section *Fumigati*. To reveal the effectiveness of this method, the strains of *A. fumigatus*, *A. lentulus*, *A. viridinutans*, *A. felis*, *A. pseudoviridinutans*, *A. wyomingensis*, *A. udagawae*, and *N. fischeri* belonging to section *Fumigati* were characterized.

Table 4 shows the RSP types of the sample strains assigned using the RSP reference list (Table 3). In this section, 18 RSPs with clearly separated peaks observed for all strains were adopted from 26 RSPs. Some RSP peaks were not matched to the reference due to peak shift or not detected. These RSPs are designated as N (Not matched) in Table 4.

**Table 4:** The RSP typing profile of the sample strains belonging to *Aspergillus* section *Fumigati*

Sample strains ^a)^	The types of biomarker RSPs																	
	L40	L39	S29	S30	S28	S31	L38	L30	L42	L33	L34	S26	L32	L26	S24	L27	S23	S16
IFM 53842 (Afu)	I	I	I	I	I	I	I	I	I	I	I	I	I	I	I	I	I	I
IFM 54229 (Afu)	I	I	I	I	I	I	I	I	I	I	I	I	I	I	I	I	I	I
IFM 57323^NT^ (Afu)	I	I	I	I	I	I	I	I	I	I	I	I	I	I	I	I	I	I
IFM 54703^T^ (Al)	II	I	I	II	I	I	II	I	III	I	I	I	II	II	I	II	II	II
IFM 47457 (Al)	II	I	I	II	I	I	II	I	III	I	I	I	II	II	I	II	II	II
IFM 58399 (Al)	II	I	I	II	I	I	II	I	III	I	I	I	II	II	I	II	II	II
IFM 60648 (Al)	II	I	I	II	I	I	II	I	III	I	I	I	II	II	I	II	II	II
IFM 61392 (Al)	II	I	I	II	I	I	II	I	III	I	I	I	II	II	I	II	II	II
IFM 62073 (Al)	II	I	I	II	I	I	II	I	III	I	I	I	II	II	I	II	II	II
IFM 62096 (Al)	II	I	I	II	I	I	II	I	III	I	I	I	II	II	I	II	II	II
IFM 47045^T^ (Av)	II	I	I	III	I	I	II	I	IV	II	I	I	II	II	II	II	II	II
IFM 55266 (Ap)^b)^	II	I	I	II	I	II	N	I	II	III	I	I	II	II	N	III	II	II
IFM 62075 (Ap)	II	I	I	II	I	II	II	I	II	III	I	I	II	II	I	III	II	II
IFM 59564 (Afe) ^b)^	II	I	I	II	I	II	I	N	III	II	I	I	II	N	N	III	II	II
IFM 62093 (Afe)	II	I	I	II	I	II	I	N	III	II	I	I	II	N	N	III	II	II
IFM 60053 (Afe)	II	I	I	II	I	II	I	N	III	II	I	I	II	N	N	III	II	N
IFM 62083 (Aw) ^b)^	II	I	N	II	I	II	N	I	N	II	I	I	II	N	I	I	II	II
IFM 46972^T^ (Au)	II	I	I	II	I	II	II	I	V	III	I	N	III	III	I	III	II	II
IFM 46973 (Au)	II	I	I	II	I	II	II	I	V	III	I	I	III	III	I	III	II	II
IFM 5058 (Au)	II	I	I	II	I	II	II	I	V	III	I	I	III	III	I	III	II	II
IFM 51744 (Au)	II	I	I	II	I	II	II	I	V	III	I	N	III	III	N	III	II	N
IFM 53868 (Au)	II	I	I	II	I	N	II	I	V	III	I	I	III	III	I	III	II	II
IFM 61606 (Au)	II	I	I	II	I	II	II	I	V	III	I	N	III	III	I	III	II	II
IFM 62070 (Au)	II	I	I	II	I	N	II	I	V	III	I	I	III	III	I	III	II	II
IFM 62100 (Au)	II	I	I	II	I	II	II	I	V	III	I	I	III	III	I	III	II	II
IFM 57324^T^ (Nf)	II	I	I	II	I	I	II	I	II	I	I	I	II	II	I	II	II	II

^a)^ Abbreviations: Afu; *A. fumigatus*, Afe; *A. felis*, Al; *A. lentulus*, Ap; *A. pseudoviridinutans*, Av; *A. viridinutans*, Au; *A. udagawae*, Aw; *A. wyomingensis* and Nf; *N. fischeri*

^b)^ *A. felis*, *A. pseudoviridinutans* and *A. wyomingensis* are former *A. viridinutans*

**Fig. 3:**
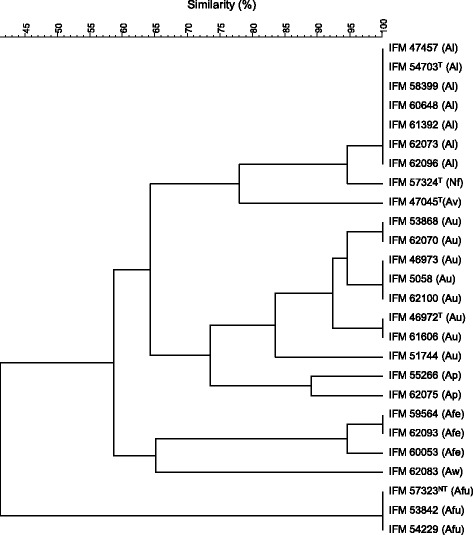
The result of the UPGMA cluster analysis of the RSP typing of the strains belonging to *Aspergillus* section *Fumigati*. Abbreviations: Al; *A. lentulus*, Nf; *N. fischeri*, Av; *A. viridinutans*, Au; *A. udagawae*, Ap; *A. pseudoviridinutans*, Afe; *A. felis*, Aw; *A. wyomingensis*, and Afu; *A. fumigatus*

The distribution of the RSP types within species tended to be consistent, and the variation was assumed to be small. This allowed the typing of sample strains to be conducted using the mass list of the genome-sequenced strain. Fig. 3 shows the result of UPGMA cluster analysis based on the RSP typing profile. In the dendrogram based on RSP typing, every species formed one general cluster.

The RSP types of *A. lentulus* strains completely match those of the type strain. Because *A. lentulus* was originally regarded as a sibling species of *A. fumigatus*, it has proved difficult to discriminate them morphologically [34]. Several mass spectrometric strategies based on mass spectral fingerprinting have been able to discriminate these species [35, 36]; however, these reports do not record the criteria used for discrimination. Our proposed RSP typing, on the other hand, can discriminate these species based on variations in RSPs. The types of 9 RSPs (L40, S30, L38, L42, L32, L26, L27, S23 and S16) differed from that of *A. fumigatus*, which can be used for discrimination between *A. fumigatus* and *A. lentulus*. Interestingly, *N. fischeri* is very close to the *A. lentulus* cluster, in which only L42 was different from that of *A. lentulus*. This species is close to the *A. fumigatus* cluster in the case of the β-tubulin gene as shown in Fig. 2b. Such differences are likely to be caused by the difference in genes used: the tree compiled using RSP typing (Fig. 3a) is constructed based on a combination of the types of 18 RSPs that reflect 18 different housekeeping genes.

RSP typing has demonstrated that *A. udagawae* strains form a relatively clear-cut cluster. Although some RSP peaks were not detected clearly, moderate concordance with the reference mass of the type strain was confirmed. Five RSPs (L39, S29, S28, L30, and L34) are further matched completely to those of *A. fumigatus*, whereas three RSPs (L42, L32, and L26) are totally different from the other species. These RSPs can therefore be used to discriminate *A. udagawae* from other species. The three strains (IFM 5058, IFM 51744, and IFM 53868) were formerly reported as variant isolates of *A. fumigatus* (or *Aspergillus* sp.) but were re-identified, using multiple genes (β-tubulin, hydrophobin, and calmodulin), as *A. udagawae* [33]. In RSP typing, these strains are included in the *A. udagawae* cluster, supporting this re-identification.

In contrast to the high conformity of RSPs in *A. lentulus* and *A. udagawae*, former *A. viridinutans* strains (*A. felis*, *A. pseudoviridinutans*, and *A. wyomingensis*) do not form an obvious cluster. Interestingly, the type strain of *A. viridinutans* (IFM 47045^T^) is located separately from other former *A. viridinutans* strains. The diversity of *A. viridinutans* has already been reported, and this species was divided into some species [37–39]. The strains IFM 55266 and 62075, IFM 59564, 60053 and 62093, and IFM 62083 were re-identified as *A. pseudoviridinutans, A. felis* and *A. wyomingensis*, respectively. In each species, the high conformity of RSPs was indicated.

## Conclusions

In the context of the ongoing conversion, on the basis of fungal taxonomy, from morphology to molecular phylogeny, molecular biological methods have been adopted for the identification and discrimination of fungal strains. To avoid misidentification of closely-related species, especially within section *Fumigati*, the reliability of identification results increases on increasing the numbers of evaluation points (*i.e.*, the numbers of genes or proteins). This gives MALDI-TOF MS great potential, since many proteomic peaks that assist with the identification of fungal species are processed. Our proposed RSP typing represents the next generation of mass spectral identification/discrimination of fungal strains, in that it supersedes current mass spectral fingerprinting, which is simple pattern-matching without peak assignment.

The merits of RSP typing are (1) it requires no commercial database and (2) it can be used to perform phylogenetic analysis. As for the first point, RSP typing requires reference to the RSP biomarker lists, via the internet, constructed from the public protein databases. At this time, of course, commercial mass spectral databases are more substantial than available RSP information. However, as whole genome-sequencing of fungal species progresses, information on RSPs is expected to expand exponentially in the near future. Although we initially encountered a confused situation as concerns the protein information registered on the public protein databases, we have successfully corrected the errors in the amino acid sequences and the names of representative *Aspergillus* RSPs. The sequences and expressed mass of RSPs of other fungal species can now be easily verified and corrected by homology analysis using the sequence list summarized in Supporting Information Additonal file 2: Table SI-2.

The second benefit of this method is valuable, because the identification results have a phylogenetic rationale: they relate to a combination of more than a dozen housekeeping proteins. This method can eliminate the influence of growth and experimental conditions, if only the RSP peaks are observed. RSPs are one of the most expressed proteins, and RSP fractions are easily collected by cell-grinding and ultracentrifugation. The discriminatory power of the RSP typing appears to be comparable with the conventional β-tubulin gene analysis. This method would therefore be suitable for species identification and discrimination at strain to species level. Because RSP typing can characterize the strains within section *Fumigati*, this method is potentially a powerful and reliable tool in the field of clinical microbiology.

## Methods

### Cell culture and preparation of ribosomal protein samples

The strains of the 14 species used in this study are summarized in Table 1. All sample strains were provided by Chiba University’s Medical Mycology Research Center (Chiba, Japan) and were grown in potato dextrose broth (PDB) medium at 25 °C for three days. After incubation, harvesting and the preparation of the ribosomal fractions were similar to the methods described in our previous paper [28]. The cultured mycelia were harvested by centrifugation and ground between zirconia silica beads. After removing the beads and cell debris by centrifugation, the fungus lysates were subjected to ultracentrifugation. The resulting ribosome fraction was solubilized in 20 - 50 μL of 50% acetonitrile containing 1% trifluoroacetic acid (TFA), and then subjected to MALDI–TOF MS measurement. Detailed sample preparation procedures are shown in Supporting Information Additional file 5: Figure SI-15.

### MALDI–TOF MS measurements

Sample preparation, apparatus, and MALDI–TOF MS data acquisition methods were similar to those described in our previous papers [18–25]. The ribosomal protein sample solution (ca. 1 μL) was spotted onto the MALDI target. About 1 μL of a sinapinic acid matrix solution at a concentration of 20 mg/mL in 50% acetonitrile with 1% TFA was then overlaid and dried in air. The MALDI–TOF MS measurements were performed using an AXIMA CFR-plus time-of-flight mass spectrometer (Shimadzu/Kratos, Kyoto, Japan) in positive linear mode. At least nine mass spectra for each sample were collected by each of three repeated measurements for each of three sample spots (total 3 spots × 3 measurements). External mass calibration was carried out using three peaks of ACTH (human, 1-24) ([M + H]^+^, *m/z* 2932.6) and myoglobin ([M + H]^+^, *m/z* 16952.6 and [M + 2H]^2+^, *m/z* 8476.8) as references.

### Calculation of the theoretical mass of RSPs

The amino acid sequence of each RSP was obtained from UniProtKB [27]. Because the genome sequence of *A. viridinutans* IFM 54703^T^, sequenced by Chiba University’s Medical Mycology Research Center, was not registered on the public databases at this time, in-house draft genome sequence data were used. The annotated RSP sequences of *A. viridinutans* have been deposited at DDBJ under the accession numbers LC213039-LC213063. The amino acid sequences of L39 and S21 of *A. niger* were not determined from shotgun sequences in the database. Therefore, these sequences were determined and have been deposited at DDBJ under the accession numbers LC255002 and LC215003. The sequence mass of each RSP was predicted using a Compute pI/Mw tool on the ExPASy proteomics server [40]. After taking into account the post-translational modifications as effected in our previous paper [28] (such as N-terminal methionine loss, acetylation, methylation and hydroxylation), the theoretical mass of each expressed RSP was calculated as [M + H]^+^ ion. Detailed construction procedure of the ribosomal protein biomarker list together with peak assignments are shown in Supporting Information Additional file 5: Figure SI-16.

### Phylogenetic analysis of RSPs

The observed masses of each RSP were compared with the reference masses constructed in this study. The matching of the average observed masses to the reference masses was judged from errors within 150 ppm. The results of mass matching were designated as RSP types. The RSP typing profiles for each sample strain were processed using UPGMA to build a dendrogram cluster for analysis employing a categorical coefficient, using BioNumerics software (version 3.5; Applied Maths, Kortrijk, Belgium).

The partial DNA sequence of β-tubulin was obtained from the UniProtKB and the alignment of the sequences was performed using ClustalW [41] software. The dendrogram was constructed using MEGA6 [42] software.

## Additional files

Additional file 1: Table SI-1. The accession number (TrEMBL) of ribosomal protein biomarkers of genome-sequenced strains used in this study. (DOCX 22 kb)

Additional file 2: Table SI-2. Corrected amino acid sequences and relating information of RSPs. (DOCX 80 kb)

Additional file 3: Mass spectra of genome sequenced sample strains used in this study. **Figure SI-1.** Mass spectra of RSPs of *N. fischeri* NRRL 181^T^. **Figure SI-2.** Mass spectra of RSPs of *A. lentulus* IFM 54703^T^. **Figure SI-3.** Mass spectra of RSPs of *A. viridinutans* IFM 47045^T^. **Figure SI-4.** Mass spectra of RSPs of *A. udagawae* IFM 46973^T^. **Figure SI-5.** Mass spectra of RSPs of *A. clavatus* NRRL 1^NT^. **Figure SI-6.** Mass spectra of RSPs of *A. niger* CBS 513.88. **Figure SI-7.** Mass spectra of RSPs of *A. kawachii* IFO 4308. **Figure SI-8.** Mass spectra of RSPs of *A. flavus* NRRL 3357. **Figure SI-9.** Mass spectra of RSPs of *A. oryzae* RIB 40. **Figure SI-10.** Mass spectra of RSPs of *A. nidulans* FGSC A4. (PPTX 472 kb)

Additional file 4: Post-translational modifications. **Figure SI-11.** Peak shift (+42 Da) of S24 with acetylation. (a) *A. fumigatus* A1163, (b) *A. viridinutans* IFM 47045^T^, (c) *A. clavatus* NRRL 1^NT^, (d) *A. niger* CBS 513.88, (e) *A. flavus* NRRL 3357, and (f) *A. nidulans* FGSC A4. **Figure SI-12.** Methylation of L42. (1) Peak shift of +14 Da from sequence mass in L42 of the *Aspergillus* species; (a) *A. fumigatus* A1163, (b) *N. fischeri* NRRL 181^T^, (c) *A. lentulus* IFM 54703^T^, (d) *A. viridinutans* IFM 47045^T^, (e) *A. udagawae* IFM 46973^T^, (f) *A. clavatus* NRRL 1^NT^, (g) *A. niger* CBS 513.88, (h) *A. flavus* NRRL 3357, and (i) *A. nidulans* FGSC A4. (2) Amino acid sequences around Lys-55. **Figure SI-13.** Dihydroxylation of S23. (1) Peak shift of +32 Da from sequence mass in L23 of *Aspergillus* species; (a) *A. fumigatus* A1163, (b) *N. fischeri* NRRL 181^T^, (c) *A. niger* CBS 513.88, (d) *A. flavus* NRRL 3357, and (e) *A. nidulans* FGSC A4. (2) Amino acid sequences around Pro-64. **Figure SI-14.** Peak shift (+28 Da) of S27 with dimethylation. (a) *A. fumigatus* A1163, (b) *A. niger* CBS 513.88, (c) *A. flavus* NRRL 3357, and (d) *A. nidulans* FGSC A4. (DOCX 187 kb)

Additional file 5: Figure SI-15. Detailed experimental protocols. Detailed sample preparation procedures. **Figure SI-16.** Detailed construction procedure of the ribosomal protein biomarker list together with peak assignments. (PPTX 61 kb)

## Abbreviations

CDS: Coding DNA sequences
MALDI-TOF MS: Matrix-assisted laser desorption ionization time-of-flight mass spectrometry
MLST: Multi-locus sequence typing
RSP: Ribosomal subunit protein
UPGMA: Unweighted pair group method with arithmetic mean
