# Supplementary material for: Ribosomal subunit protein typing using matrix-assisted laser desorption ionization time-of-flight mass spectrometry (MALDI-TOF MS) for the identification and discrimination of Aspergillus species
Source: BMC Microbiol. 2017 Apr 26;17:100. doi: 10.1186/s12866-017-1009-3 (PMC5405522; doi:10.1186/s12866-017-1009-3)
Supplement: Supplementary file 1 — The accession number (TrEMBL) of ribosomal protein biomarkers of genome-sequenced strains used in this study. (DOCX 22 kb) [file 12866_2017_1009_MOESM1_ESM.docx]

Table SI-1 The accession numbers (TrEMBL) of ribosomal protein biomarkers of genome-sequenced strains used in this study.

| RP name | *N. fischeri*  IFM 57324^T^ | *A. lentulus*  IFM 54703^T^ | *A. udagawae*  IFM 46973^T^ | *A. clavatus*  IFM 60676^NT^ | *A. niger*  CBS 513.88 | *A. kawachii* IFO 4308 | *A. flavus* NRRL 3357 | *A. oryzae*  RIB 40 | *A. nidulans*  FGSC A4 |
| --- | --- | --- | --- | --- | --- | --- | --- | --- | --- |
| L26 | A1DNH6 | A0A0S7DJS2 | A0A0K8LIR5 | A1CU11 | A2QXI7 | G7XG87 | B8NEA6 | Q2U874 | Q5BFW0 |
| L27 | A1D3Z3 | A0A0S7E8Y2 | A0A0K8LPP4 | A1CR44 | A2RB04 | G7XSR1 | B8NMV9 | XP_001818935.2^b^) | Q5B5F8 |
| L29 | A1D4U6 | -^a)^ | -^a)^ | A1CRX5 | A2Q8M9 | -^a)^ | B8MY86 | XP_003189205.1^b)^ | C8VUC5 |
| L30 | A1DGL4 | A0A0S7DWD2 | A0A0K8LGV8 | A1CSY0 | A2QZG7 | G7XHU0 | B8NBK4 | Q2TZP4 | Q5B048 |
| L31 | A1DP45 | A0A0S7DDB7 | A0A0K8LJE3 | A1CUG4 | A2QSB9 | G7Y0M0 | B8N446 | XP_003189527.1^b)^ | C8VGD2 |
| L32 | A1DIJ8 | A0A0S7DL03 | A0A0K8LH39 | A1C7X0 | A2R659 | G7XX33 | B8NQW4 | Q2UAU8 | Q5AWH6 |
| L33 | A1D7K2 | A0A0S7DQ65 | A0A0K8LBE2 | A1CJW6 | A2QES9 | -^a)^ | B8N023 | XP_003189289.1^b)^ | Q5B900 |
| L34 | A1CZ01 | A0A0S7DXN0 | A0A0K8LRA1 | -^a)^ | A5AAU3 | G7XF07 | B8NH75 | XP_001822075.2^b)^ | Q5B4K8 |
| L35 | A1D328 | A0A0S7E103 | A0A0K8LKJ2 | A1CQ64 | -^a)^ | G7XRF6 | B8NJV8 | XP_003190676.1^b)^ | Q5BE02 |
| L36 | A1CXN3 | A0A0S7DZJ9 | A0A0K8L642 | A1CH55 | A2QI04 | G7X6Q0 | B8N9U3 | Q2UGI4 | Q5B4S8 |
| L37 | A1D834 | A0A0S7DLE8 | A0A0K8LDD2 | A1CJE6 | XP_001394915.2^b)^ | -^a)^ | B8NUP1 | XP_003190519.1^b)^ | Q9C0T1 |
| L38 | A1CXI8 | A0A0S7DVJ2 | A0A0K8L6R6 | A1CH97 | A2QI48 | G7X6U4 | B8N9Q2 | XP_003189771.1^b)^ | C8V6G7 |
| L39 | A1CZG2 | -^a)^ | -^a)^ | -^a)^ | -^a)^ | -^a)^ | -^a)^ | XP_003189071.1^b)^ | C8VN49 |
| L40 | A1D4L7 | -^a)^ | A0A0K8LP90 | A1CRQ4 | A2Q831 | -^a)^ | B8N3G7 | XP_001820063.2^b)^ | Q5B614 |
| L42 | A1DGC3 | -^a)^ | A0A0K8LEN5 | A1CSM9 | A2QKM9 | G7XHH3 | B8NBW0 | XP_003190841.1^b)^ | Q5AZU9 |
| L43 | A1DIQ3 | A0A0S7DQ87 | A0A0K8LIV6 | A1C821 | A2QK12 | G7XVN6 | B8NPV3 | XP_003190126.1^b)^ | -^a)^ |
| S16 | A1DGX5 | A0A0S7DTG2 | A0A0K8LF72 | A1C6A0 | A2QD71 | G7X4X1 | B8NB36 | Q2U048 | C8V3D8 |
| S21 | A1CZ53 | A0A0S7DRI0 | A0A0K8LRE4 | A1CEU9 | -^a)^ | G7XE47 | B8NLJ6 | XP_003190415.1^b)^ | Q5B6K7 |
| S23 | A1D3D1 | A0A0S7E010 | A0A0K8LM29 | A1CQG8 | A2QQ19 | G7XKM4 | B8N7F1 | Q2UBN5 | Q5BDN5 |
| S24 | A1D993 | A0A0S7DJD0 | A0A0K8LDS5 | A1CG24 | A2QYA0 | G7XW21 | B8NJ15 | Q2U258 | Q5ARI3 |
| S26 | A1D3U8 | A0A0S7E0A6 | A0A0K8LMS6 | A1CQY4 | A2RB80 | G7XSX0 | B8NNG9 | XP_003189422.1^b)^ | Q5B165 |
| S27 | A1D846 | -^a)^ | A0A0K8LBA7 | A1CJD3 | A2QXP2 | G7XFR4 | -^a)^ | XP_003190521.1^b)^ | Q5B3V3 |
| S28 | A1DCB3 | -^a)^ | A0A0K8LFC7 | A1CDD5 | A2R1F7 | G7XRN2 | B8MZ58 | XP_003189250.1^b)^ | Q5AYJ8 |
| S29 | A1DNZ6 | -^a)^ | -^a)^ | A1CUB3 | A2QLP1 | -^a)^ | B8N234 | XP_003189521.1^b)^ | C8V7J5 |
| S30 | A1DL63 | -^a)^ | -^a)^ | A1CMS3 | A2QVJ9 | -^a)^ | B8N786 | XP_003190070.1^b)^ | C8VA41 |
| S31 | A1D6U5 | A0A0S7DPQ3 | A0A0K8LB72 | A1CKM6 | A5AA93 | G7XPI1 | B8N3Q5 | Q2UKT8 | G5EB17 |

^a)^ The protein is not registered in the databases. See Table SI-2.

^b)^ NCBI reference sequence accession numbers.
