# Supplementary material for: Ribosomal subunit protein typing using matrix-assisted laser desorption ionization time-of-flight mass spectrometry (MALDI-TOF MS) for the identification and discrimination of Aspergillus species
Source: BMC Microbiol. 2017 Apr 26;17:100. doi: 10.1186/s12866-017-1009-3 (PMC5405522; doi:10.1186/s12866-017-1009-3)
Supplement: Supplementary file 2 — Corrected amino acid sequences and relating information of RSPs. (DOCX 80 kb) [file 12866_2017_1009_MOESM2_ESM.docx]

**Table SI-2**. Corrected amino acid sequences and relating information of RSPs.

| RP name | Accession numbers of protein sequence (TrEMBL) | Accession numbers of gene sequence (NCBI) | Post-translational modifications | Corrected amino acid sequences | Corrected exon/intron structures (putative) |
| --- | --- | --- | --- | --- | --- |
| *N. fischeri* NRRL 181^NT^ | | | | | |
| L27 | A1D3Z3 |  | - | MKFMKVGRVAIITRGRYAGKKVVIVQPNDTGSKAHPFPYAIVAGIERYPLKVTRRMGKKMVEKRSRIKPFIKVVNYNHLMPTRYTLELEGLKGAVSQETFKEVSTREDAKKTVKKALEDRYTSGKNRWFFTPLRF | ATGAAGTGTTAGTTGAATGCCGTCTCATCTCGAGTTCTCAATTATTTGAATATCAGTTCATACTGACAGGATGGTGAAATAGTCATGAAAGTGGGCCGTGTGGCCATCATCACCCGTGGCCGTTACGCCGGTAAGAAGGTACGACAAGCCTTTTCTTTTCCTACGTGTTCTAGCGGAAATTTGGTTGAGGATGGAGGGTCTGGTTCGAAACTTTTCGATGGTCGATTTTGAGAGGAAGAAAGAGAGACTTTCTGGACGAATTTGGGAAGGGATATCGATTTGGGATGGGATCAATTGGACATTGAGAAGGACGTGCTGACAAGGGACCTTTTCGCGAAAACACAGGTCGTCATTGTCCAGCCTAACGACACTGGCTCCAAGGCGCACCCCTTCCCCTACGCCATCGTCGCCGGTATCGAGCGCTACCCCCTCAAGGTCACCCGCCGCATGGGCAAGAAGATGGTCGAGAAGCGCAGCCGCATCAAGCCTTTCATCAAGGTCGTCAACTACAACCACTTGATGCCCACCCGTTACACTCTTGAGCTTGAGGGTCTCAAGGGTGCCGTCAGCCAGGAGACCTTCAAGGAAGTCTCCACACGCGAGGACGCCAAGAAAACCGTCAAGAAAGCCCTCGAGGACAGATACACCAGCGGCAAGAACAGATGGTTCTTCACTCCTCTGCGTACGTGCAATCCTTGCTTATTGTTACTTTGCAGTGAGATAGTTGTGTGCTGATTGTTATCCCTACACAGGTTTCTAA |
| L35 | A1D328 |  | Met-loss, Acetylation | MSTSKVKAGQLWGKSKEDLSKQLEELKTELSQLRVQKIAAGASSKTQRIHDVRKSIARVLTVINANQRAQLRLFYKNKKYTPLDLRPRLTRALRRRLTKHEATLKTEKQRKKEIHFPQRKYAVKA | ATGGTATGTTGAATGCGCAATTTTTCGATTCGGGCTTTTTTTTCGAAGCCGTTGGGGGTTCGTTATGCTGATGAATTTTAGTCGACATCCAAGGTCAAGGCTGGTCAGCTCTGGGGAAAGAGCAAGGAAGACCTTTCCAAGCAGCTCGAGGAGTTGAAGACCGAGCTCTCCCAGCTCCGTGTCCAGAAGATCGCTGCCGGTGCCTCGTCGAAGACTCAGAGAATGTGCGTTTGAACTCGAAGCCCTTCCTTGTGCATCTGCAATTCTACCTCGAAAAATGAAACCCCTCATGGCATGGAAATTTATTGGAAAGACAACGAAAACGACAGACTGGGAAGAATCGGGGCCTACCAGAATCGCAGATTGCGCAATATACACAAGATACAATATTTGGGGACATGAATGCTGATTTGCTGCGGTTCTACAGCCACGACGTTCGCAAGTCGATCGCTCGCGTTCTCACCGTCATCAACGCCAACCAGCGCGCCCAGCTCCGTCTGTTCTACAAGAACAAGAAGTACACTCCTCTTGACCTCAGACCCCGCCTCACCCGTGCCCTCCGCCGCAGACTCACCAAGCACGAGGCCACCCTCAAGACGGAGAAGCAGCGCAAGAAGGAGATCCACTTCCCCCAGCGGAAGTACGCCGTCAAGGTATGCCTCCTGCTGCATCACCATGCGGACAAAAAATCCCGCAATCGTTGAGGCATTTTCAATTCGGGCCAGCTGAGTCACTAATGATGAATTTCCACAGGCCTAA |
| L36 | A1CXN3 |  | Met-loss | MAQERSGIVVGLNKGHKTTPLNTPKTRISRTKGQSSRRTAFVRDIAREVVGLAPYERRIIELLRNTQDKRARKLAKKRLGTFSRGKRKVEDMQRVIAESRRVAGH | ATGGCGCAGGAACGTTCCGGAATCGTGGTCGGTCTGAACAAGGGCCACGTACGTTGAATCCCTCTCCCGTTTCCCAAAGAAAAAGAATGTTTCCGGTCGGTATTTGTCCAGTCGATCTCGAGAAATTTGGATTGAGGATACGGAACATAATATGGAGGGGACATATTGTCGGATTGTCAAAAGGCACAGTTCCTTCTTGAGGGAGGAAATGGGGACGACAATGGACGCGCGAATCGAACTTATTGAGCCTGACTTGACAAAAATCGATCGGAAACATTCGACTTTTGGTTATAGGAATGAGGAACAATCAGTGAAGCTAACTTGGGTTCCCATGATAGAAAACCACCCCTCTCAACACCCCCAAGACCCGGATCAGCCGCACCAAGGGCCAGTCTTCCCGCCGCACTGCCTTCGTCCGTGACATCGCCCGTGAGGTTGTCGGTCTTGCCCCTTATGAGCGTCGTATCATCGAACTTCTGAGAAACACTCAGGACAAGAGAGCTCGTAAGCTCGCCAAGAAGAGGGTATGTCACAAGTTCTGGATTTGGTCTTCACGGGTGTTACTTTGCTCGCTTCCCTGGGGACGTCATCATACGGTATCTGCGCTTCGAAATACAATTTAATTAAGCATGCGGCAACTGTCTGTTGGACACTACACGCTCCGCGAATCTAGCCATTCGAGTACGGAGAGCGCCGACCAACGCCTTGTGGAGCGAGCCTGGGGAACACTTGCTGAGGACTTTCGCAATTATTCATGAACGATGCTAACGACTTATGTGTTATAGCTCGGTACCTTCTCCCGTGGCAAGAGAAAGGTTGAGGACATGCAGAGAGTCATCGCCGAGTCCAGACGTGTGGCTGGTCACTAA |
| L39 | A1CZG2 |  | Met-loss | MPSHKSFRTKQKLAKAQRQNRPIPQWIRLRTGNTIRYNAKRRHWRKTRLGI | ATGCCGGTTAGCAGTCCTCCCCTACGATTCCGAGTTATTTCAAGATGCCAGGACTTTATTAGTCCTGCTCTCGCATCGATGAGCCGAGTTATTCGTCTGTGATGTTGAGGTGGCAGGGTGACAATTGGCTAACGTTGATTTCTGTTGAATAGAGCCACAAGAGTTTCCGCACCAAGCAGAAGCTTGCCAAAGCTCAGAGACAGAACCGTCCTATTCCCCAGTGGATTCGTCTCAGGACCGGTAACACCATCAGGTAAATACTCTCTTTTTCCCCCTACAATCCGGAGCCGTCGATCGTGTGCACTGGTGGTGTGGGAAACTCGCTTGAATCGGACTTGGATCACATCAACAACCTTGTCATCTCCTGCATCAACAAGCGCACCAGAAAAATCACACGAATGGATTTGGAACCTTGCGCGGGTTACATGGACACAAGGAAGAATGAACCAGCTGACGGGTACTCTTCTCCTCCAGATACAACGCCAAGCGGAGACACTGGCGCAAGACCCGTCTCGGTATCTAA |
| L40 | A1D4L7 |  | N-terminal scission (Release ubiquitin with 76 amino acids at the N-terminal side) | IIEPSLKALASKYNCEKSICRKCYARLPPRATNCRKKKCGHTNQLRPKKKLK | AGTACGTACAGCATCATGTTCTCCTACGCGATGTATTTGGTCTATGGATTACGAGCTAACATACCCTTTTTTCTGCGATTTTACAGTGCAGATGTAGGTCTTCCTGAGCCGTTCGGACCAACCCAGTGCCCATTTGCCCAACCTCATCCCATTCATCGACACGATATACGCGGAACATCCGGAAAAGCCATGACTGACAAGTTTCGATCAATAGTTTCGTCAAGACCCTGACGGGTAAGACCATTACCCTCGACGTCGAGTCGAGCGACACCATCGACAACGTCAAGGCCAAGATCCAGGACAAGGAGGGTATCCCCCCTGACCAGCAGCGCCTGATCTTCGCCGGTAAGCAGCTTGAGGATGGCCGCACTCTGAGCGACTACAACATTCAGAAGGTACGCGAATTCTCTATCGGACGACGACTTGGTTTCGTTTCTTTATGCGCATGGGGTATTGCCGGAAGATGCGGGTTGTTCGCTGGATTGCTGGATTGCTGGACTTGGTTTCCGGATCCTCCGGCAGGAAACGTCTGCGCTAGGAGATCGTCTGGATTGTTATGGAGGATATACGGTTATTGAGAAGGATGTGTTTGGCTGACCGGATGCTTTTGCACATATCTAGGAGTCCACTCTGCACCTGGTCCTCCGTCTGCGTGGTGGTATCATTGAACCCTCCCTCAAGGCTCTCGCTTCCAAGTACAACTGCGAGAAGTCCATCTGCCGCAAGTGCTACGTACGTCTTTGTCCCGATCGCTCGACCCGACATGTGACCCGTTACTGGGGACGCTCGCCTTGAAGTCTTTACTAACTCTATCCCTGGATAGGCCCGTCTTCCTCCCCGTGCCACCAACTGCCGTAAGAAGAAGTGCGGTCATACCAACCAGCTGCGCCCCAAGAAGAAGCTCAAGTAA |
| L42 | A1DGC3 |  | Met-loss, Methylation | MVNVPKTRRTYCKSKECHKHTQHKVTQYKAGKASLFAQGKRRYDRKQSGYGGQTKPVFHKKAKTTKKIVLRLECTACKTKKQLALKRCKHFELGGDKKTKGAALVF | ATGGTAAGGACTCATTTGCATTTTTCTTACTCGCATGCTGTTTCCATCCTGGAGATTCGGAGAAGTCTGTCGAAAACTCGGTTGGCGATGAAAGGAGATGTTTGTTCGGGTTCGAGGACGATGAAGTTGCGGACAATGGAAAAACGGACGAAAATGCTATCGATCGCAACTATGATACGTCTCGCTCGGATATCAGAAATTGAACTATTTTCATGCGTCAGAGTTTAGAGGAGAAATATGGGATATTCCTCGACGCGATGAGGACGCAACGGACGCACTGGCAACGAAAGACTCAGCGCATCAAACACGGAAAATAACCACGAATATTCGCTCGCCAGAAGAACCTGTGACATAGTCTCTGCCAGTGATCTGAATCTCTTCCCACGATAACCTCTACCGAGTTGGACTGGATTCTCCGATGAACCGGGACATGGAAACAGGCAGCACAAGAAGTTGTTGGATTGTAGTGGACAGTTGCTGATAGCTCTTGCGCTCTAGGTCAACGTTCCAAAAACCCGCCGGACGTACTGCAAGTCCAAGGAGTGCCACAAGCACACCCAACACAAGGTCACCCAGTACAAGGCTGGCAAGGTGAATATAACCCAAACGCCACGGTAGATGAGTTGGCACAGGCACTGATGATGGATCTATTAGGCCTCCTTGTTCGCCCAGGGTAAGCGTCGTTACGACCGCAAGCAGAGCGGTTACGGTGGTCAGACCAAGCCTGTCTTCCACAAGAAGGCCAAGACTACCAAGAAGATCGTCCTGCGTCTTGAGTGCACTGCCTGCAAGACCAAGAAGCAGCTCGCTCTGAAGCGTTGCAAGCACTTCGAGTTGGGGTACGTTGAGTCCGCGAATGTGGCGATACCTGGTCCATGCATACTAACGTACGCTTTTTGCTGTTTAGTGGTGACAAGAAGACCAAGGGTGCTGCTCTTGTTTTCTAA |
| L43 | A1DIQ3 |  | Met-loss | MTKRTKKVGITGKYGTRYGASLRKQVKKMEITQHARYVCTFCGKNTVKRQAVGIWECKGCKKTVAGGAYTVSTPAAAATRSTIRRLREIAEV | ATGACGAAGCGCACTAAGAGTGAGTGATTTTGATCGAGATAAACCACGGAGAAAGGATGAAAAAATGATAGAAATACTGACTGCATGCTTTTAGAGGTCGGTATCACGGGTAAATATGGTACCAGGTATGCTTTTCGCCCTCTCACATCGAAAAATCGACCTGAAGTCGTCTGCATTTCGCTCTGCTCCATCGAAACCGGAATCCAAAGCGCGGAATATCGAATTCAGGAGAGCTAGAATGGTTGGTTGACATGGCTTGTTCATTACAGATACGGTGCCTCCCTGCGTAAGCAGGTGAAAAAGATGGAAATCACCCAGCACGCCCGCTACGTCTGCACCTTCTGCGGAAAGAACACCGTCAAGCGCCAGGCTGTTGGTATCTGGGAGTGCAAGGGCTGCAAGAAGACCGTTGCCGGTGGTGCCTACACCGTCTCGTAAGTCGACCACTCGCGCTCCCCCTTGTTCCTAGCATTCTCTGTCGTCGCGCCTTGGAACAGGCCAGACTGGTGAAAGAAAAGCCTGGAGCATTCACTGTGACACGGATACTGACCGATGTAATTGTACTACAGCACACCCGCCGCCGCTGCCACCCGCTCGACCATCCGTCGTCTCAGAGAAATCGCGGAGGTTTAA |
| S27 | A1D846 |  | Met-loss,  Methylation (2) | MVLAVDLLNPTPQAEARKHKLKTLVPAPRSFFMDVKCPGCFTITTVFSHAQTVVVCAGCSTVLCQPTGGKARLTEGCSFRRK | ATGGTAGGTTTTGCTTGCATTTCGGCTTCATGCAACACCTTGGGATATCCCCTAGCCGTTCTGCTGGACGTCTGCACAGACTACGAGCTTGCTGCCCGTCGTGTGCTCGCCGTCGCCGGACCAGTTTTCATCACAAAATGTCATCAAGGTATCATCGGCTAACACAATTTCTTTCTTTCACAATAGGTTCTCGCGGTCGACCTCCTCAACCCTACTCCTCAGGCTGAGGCTCGCAAGCACAAGCTTAAGGTATGATACCGAAATACACACCTCTCCACCAAGGTCCCGGATCCGATGGCTGATGTTGGGAATCGCTTCGACAAATAGACCCTTGTGCCTGCTCCCCGTTCCTTCTTCATGGACGTCAAGTGCCCCGGCTGCTTCACCATCACCACCGTCTTCTCCCACGCCCAGACCGTCGTCGTCTGCGCCGGCTGCTCGACCGTCCTTTGCCAGCCCACCGGTGGCAAGGCCAGACTCACTGAGGGCTGCTCCTTCCGGAGGAAGTAA |
| S29 | A1DNZ6 |  | Met-loss | MTHESVWYSRPRKFGKGSRECRVCAHRAGLIRKYGMDICRQCFREKAQDIGFYKYR | ATGACTCACGAGTCCGTTTGGTACAGCCGGCCCCGCAAGTAAGGATATTCTTCAATTCTGGAATGCGTCGATACGATTTGGCTAATTTGTTATCTCTCTATAGGTTCGGCAAGGGTTCCCGTGAATGGTGTGTGAACGAAATTCCCCAAGCGATACCAGGAGGATAGGGTCGAAATATTATGGATTGCGAGCAGGAATGGCTAATCAGATATTTTGCGTTGAAACAGCCGTGTTTGCGCCCACCGCGCTGGTCTGATCCGCAAGTACGGGGTATGGAAATGAGCCAACTATCGAGATTAACAAGCGTTGGATATGCGACTGATAGTGGTTATAGATGGACATCTGCAGACAGTGCTTCCGTGAGAAGGCTCAGGACATCGGTTTCTACAAGGTTGGTTCCCACATACGCCGCAGAGTCGGAACACCATGGCCATGGTCAACCCGACGACACTCGGAGAACTGGTTGGCAGAAGAAAGAGCGGATCACTAACAGAGTTACCTCATACAGTACCGTTAA |
| S30 | A1DL63 |  | Met-loss | MGKVHGSLARAGKVKSATPKVEKQEKKKEPKGRALKRLKYTRRFVNVTMTGGKRKMNPNPTS | ATGGGTAAGGTTCACGGATCTCTCGCCCGTGCGGGTAAGGTCAAGTCTGCGACTCCTAAGGTGAGTTCTTCTTGTTCTTCCTTATAATGAAGTTTTGATCTCCGCGTTCCTACTGGGAAGGGTTCATCGTCGCCACCGAAGAGGAGGGATTTTAAAGGAAGATTTTTGCAGCGATCCGACATGCAGAAACGGCAGAGAGGATATATGCGAAGACAATATGATGAACCGATGTTTTCCAGGGAGATTTTTGCTGAGTCAAGAGTGCTGATCGGTTCTGTTCTACTGTACAGGTCGAGAAGCAAGAGAAGAAGAAGGAGCCCAAGGGCCGTGCTCTGAAGCGCCTCAAGTACACCCGCCGTTTCGTCAACGTTACCATGACTGGTGGCAAGCGGAAGGTACGTTCAAATCCAGCTCGATACAAAGGATGCATATATGCTCTGAGCGGGAGAGTTGGGTTCTGTTCTTAGAAGGAACCAGTTCTCCGTCCAGCCAGCAGATTTTGCAGGAGGACCTTGGCATCGAAAAGGCATCAGACGCTGACAGGGCATTCGTTACAGATGAACCCCAACCCCACTTCTTA |
| S31 | A1D6U5 |  | N-terminal scission (Release ubiquitin with 76 amino acids at the N-terminal side) | AKKRKKKVYTTPKKIKHKRKKTKLAVLKYYKVDGDGKIERLRRECPSPECGAGIFMAAMHNRQYCGKCHLTYVFDESK | ATGCAGATCTTCGTCAAGACCCTTACGGGTAAGACTATCACCCTCGAGGTGGAGTCTTCGGACACCATTGACAATGTCAAGTCCAAGATCCAGGGTACGCAACCCCTCCGAGCATCGTGACTGGAGAGACTCTGAATCTAACTCGATAACAGACAAGGAGGGAATCCCCCCCGACCAGCAGCGTCTGATCTTCGCTGGCAAGCAGCTCGAGGACGGCCGTACCCTCTCTGACTACAACATCCAGAAGGAGTCGACCCTGCACCTCGTGCTCCGTCTGCGTGGTGGTGCCAAGAAGCGCAAGAAGAAGGTCTACACCACCCCCAAGAAGATCAAGCACAAGCGCAAGAAGACCAAGCTCGCTGTCCTCAAGTACTACAAGGTTGACGGCGATGGCAAGATCGAGCGTCTCCGCCGCGAGTGCCCCTCCCCCGAGGTATGTTAACTTGCTCTTCTGAAAAAGTGAGACTCCAGCTAATGACTTATAGTGCGGTGCTGGTATCTTCATGGCTGCTATGCACAACCGTCAGTACTGCGGCAAGTGCCACCTCACCTACGTCTTTGACGAGTCCAAGTAA |
| *A. lentulus* IFM 54703^T^ | | | | | |
| L27 | A0A0S7E8Y2 |  | - | MKFMKVGRVAIITRGRYAGKKVVIVQPNDTGSKAHPFPYAIVAGIERYPLKVTRRMGKKMVEKRSRIKPFIKVVNYNHLMPTRYTLELEGLKGAVSQETFKEVSTREDAKKTVKKALEDRYTSGKNRWFFTPLRF | ATGAAGTGTTAGTTGAATGCCGTCTCATCTCGAGGTCTCAATTATTTGAATATCAGTTCATACTGACCGGATGGTGAAATAGTCATGAAAGTGGGCCGTGTGGCCATCATCACCCGTGGCCGTTACGCCGGTAAGAAGGTACGACAAGCCTTTTCTTTTCCTACGTGTTCTAGCGAAAATTTGGATGAAGGATCTGGTTCGAAACTTTTCGATGGTTGATTTTGAGAGGAAGAAAGGGAGATGTTCTGGACGAATTGAGGAAGAGAGATCGATTTGGGATTGGATCAATTGGACTTAATGAGAAGGATGTGCTGACAAGGGACCTTTTCGCGAAATCACAGGTCGTCATTGTCCAGCCTAACGACACTGGCTCCAAGGCGCACCCCTTCCCCTACGCCATCGTCGCCGGTATCGAGCGCTACCCCCTCAAGGTCACCCGCCGCATGGGCAAGAAGATGGTCGAGAAGCGCAGCCGCATCAAGCCTTTCATCAAGGTCGTCAACTACAACCACTTGATGCCCACCCGTTACACTCTTGAGCTTGAGGGTCTCAAGGGTGCCGTCAGCCAGGAGACCTTCAAGGAAGTCTCCACACGCGAGGACGCCAAGAAGACCGTCAAGAAGGCCCTCGAGGACAGATACACCAGCGGCAAGAACAGATGGTTCTTCACTCCTCTGCGTACGTGCAATCCTTGCTTATCGTTACTTGCAGTGAGAGAGTTGTGTGCTGATCATTATCTCTCCACAGGTTTCTAA |
| L29 | - | BCLY01000004.1 (c2717022-2716229) | Met-loss | MAKSKNASQHHRSQKAHRNGIKKPKTHRYPSLKGVDPKFRRNHRHALHGTMKALKERKEGKREVA | ATGCCTACCCGTTTTTCGAAGACAAGAAAAGCGTGAGTCGAGAATTTCACCCCGTCATACACCTCGAATTGTCGAACGTCCCTTTGCTGTTGTTGTATTTGCGACGAGTCGATGCATCCATTCGCCATCATGAAACAATCATTCGGAATATTATGTATATCGTGAGACTGACCTGGATTCTTCTTCTACTACAGTCGCGGTCATGTGTCGGCCGGTTACGGTCGCGTTGGCAAGCACCGTAAGCACCCAGGTGGTCGTGGTATGGCCGGTGGTCAGCACCACCACCGTACCAACCTTGACAAGTTCCACCCTGGTTACTTCGGTAAGGTTGGTATGAGATACTTCCACAAGACCAACCAGCAGTTCTGGAAGCCCACCATCAACCTCGACAAGGTACGTTGAATGAATGCGTTTAGAGTCACTCCTAGTCCGACCCGACGGGAATCGCCGCGAGAGCAGAAGAAAGTTGCGTTGAGGAACGTGGAAGAGTAGCGGAGATATGATAGGATTGGGGAGGAGAATATGTTGTGGTTATGGACAAAAGTGGCTGATTGGGATGTCTTCCTTCTTTACAGCTGTGGTCCCTCGTCCCTACCGAGACTCGCGAAGCCTACCTGAGCGGCCAGAAGACCGACACCGCCCCCGTCATCGACCTCCTCTCCCTTGGCTACTCCAAGGTTCTGGGCAAGGGCCGTATCCCCGAGATCCCCATCGTTGTCCGCGCCAGATACTTCAGCCGGGACGCTGAGCAGAAGATCAAGGAGGCCGGTGGTGTTGTTGAGTTGGTTGCTTAA |
| L33 | A0A0S7DQ65 |  | Met-loss | MPSEHGHRLYVKGRHLSYQRSKRAVNPNTSLIKIDGVDSTEAANFYLGKKVAFVYRAKREVRGSNIRVIWGKVTRPHGNSGVVRAQFRHNLPPKSFGATVRVMLYPSNI | ATGCCTTCGGAACACGGTCACAGACGTGAGTGATCCCTCTCCGTTTAGCTGGATATCGATGTGTGCGCACAGGTTGGAGAATGGGTTTCGGGGATGGATGCGGTGCGATTATGGATTTTATAAAGGATCTTGCTAACGGGCTATTTTCTACTTGCACAGTCTACGTCAAGTAAGTTATCACGATTTTCTCATCGATTATCCGTCGGGTCGCGATCATTCGGAATGCGATATACGAATGTCAAATGTCAAATGTCGCACTGTGGGATCGGGCTAGGGGGCCAAGTCGAAACGAAATTATATGGACAGATGGGTTAATACGAATTCTCTCTATACAGGGGTCGCCACCTGAGCTACCAGCGCTCCAAGCGCGCGGTCAACCCCAACACCAGGTAGCTATCCACTGAAGATGGACGTCAAGGGATATGGGAGGCTGACACCTCGTATAGTCTGATCAAGATCGACGGTGTTGACAGCACTGAGGCCGCAAAGTGAGTATACACCGGTTGAAAGAATCACGAAACAAGGAAGAGAACATGCAGCTAAGACGGTTGTTTTACAGCTTCTACCTCGGCAAGAAGGTTGCTTTCGTTTACCGGGCGAAGCGTGAGGTTCGGGGTTCCAACATCCGGGTCATCTGGGGCAAGGTTACCCGGCCACACGGTACGTGAATTCAATACGATGGATCGGTGATATCGGCATTGCGGCCCTGTTGGACGATGGCTACTAGAGGAGCGAACAACTGACAATATCAACTTGACAGGCAACTCCGGCGTTGTCCGTGCTCAGTTCCGCCACAACCTCCCCCCCAAGTCCTTCGGTGCTACCGTCCGCGTCATGCTCTACCCCTCCAACATCTAA |
| L34 | A0A0S7DXN0 |  | Met-loss | MANNRLQYRRRNPYNTRSNKVRIIKTPGGELRYLHIKKKGTAPKCGDCGIKLPGIPALRPREYSQISRPKKTVSRAYGGSRCAGCVKDRIVRAFLIEEQKIVKKVLKESQEKAAGKR | ATGGCGAACAACAGATTGCAATACGTGAGTTTTTATACTGGAATATATTTTTACTTCGATGAAATGCTGATTCCCTTTTTTCCTACAGCGGAGACGGAACCCGTACGTTTGAAATTCCCTCCCAGAAGTTCACAATCCTATGCCCTCTGCGTCGCCTTCATCATCGGCGTTGTTCGCTTTAAATTCAGTGACATAGCGCATGATATTTGGCGCGACGAGACACAACAGTTCCGAGGTTGCCCTTGAAAGCACGATATTGATAGGTTTTGTTCTACAGGTACAACACGCGGTCCAACAAGGTCCGCATCATCAAGACTCCTGGCGGCGAGCTCCGTTACCTCCACATCAAGAAGAAGGGCACTGCTCCCAAGTGCGGTGACTGCGGCATCAAGCTCCCCGGCGTGAGTGCTCCCGAGTGAAATTCTGGCTAGGAAGTGGTCTTCTGTGCGCTTTTTTGGGTGTCCTTCGATCGTCGTACTGTCGAGTCACGAGAGGACAAGTTCGGGAACGGAAAGCGCACATTTGATTGCCTCTTGCCAATTGATAGGAAGATGAGCAGGATCACTGATTACAATGCTTAGATTCCCGCCCTCCGCCCCCGTGAATACTCCCAGATCTCCCGGCCCAAGAAGACCGTCAGCCGTGCCTACGGTGGTTCTCGCTGCGCCGGTTGCGTCAAGGACCGTATCGTCCGTGCTTTCCTGATTGAGGAGCAGAAGATCGTCAAGAAGGTCCTCAAGGAGTCTCAGGAGAAGGCCGCTGGCAAGCGCTAA |
| L35 | A0A0S7E103 |  | Met-loss, Acetylation | MSTSKVKAGQLWGKSKEDLSKQLEELKTELSQLRVQKIAAGASSKTQRIHDVRKSIARVLTVINANQRAQLRLFYKNKKYTPLDLRPRLTRALRRRLTKHEATLKTEKQRKKEIHFPQRKYAVKA | ATGGTATGTTGAATGCGCAATTTTTCGATTCGGGCTTTTTTTCGAAGCTGTTGGGGGTTCGTTATGCTGATGAATTCTAGTCGACATCCAAGGTCAAGGCTGGTCAGCTCTGGGGAAAGAGCAAGGAAGACCTTTCCAAGCAGCTCGAGGAGTTGAAGACCGAGCTCTCCCAGCTCCGTGTCCAGAAGATCGCTGCCGGTGCCTCGTCGAAGACTCAGAGAATGTGCGTTTGATCTCGAAACCCTTCCTTGTGCATCTGCAATTCTACATCGAAAGAAAAAATACTACAAATGGTCCCCAAAATGTGGCAATGAATTTATCGGAGGAACAGCGGAATCGACACATTGAGAGAAATTGGGGCCTGCAAAAACCGCAGATTGCGCAATATACACAAGATACAATATTGGGGACAGGAATGCTGATTTGCTACGGTTCTACAGCCACGACGTTCGCAAGTCGATCGCTCGCGTTCTCACCGTCATCAACGCCAACCAGCGCGCCCAGCTCCGTCTGTTCTACAAGAACAAGAAGTACACTCCTCTTGACCTCAGACCCCGCCTCACCCGTGCCCTCCGCCGCAGACTCACCAAGCACGAGGCCACCCTCAAGACGGAGAAGCAGCGCAAGAAGGAGATCCACTTCCCCCAGCGGAAGTACGCCGTCAAGGTATGCCTCCCGCCGCATCTCCATTCGCACCGACAAATCCCGCAATCGTGCGGCATATTCAATTCGAACCAGCAGAGTCACTAACGAGCAATTTCCACAGGCCTAA |
| L36 | A0A0S7DZJ9 |  | Met-loss | MAQERSGIVVGLNKGHKTTPLNTPKTRVSRTKGQSSRRTAFVRDIAREVVGLAPYERRIIELLRNTQDKRARKLAKKRLGTFSRGKRKVEDMQRVIAESRRVAGH | ATGGCGCAGGAACGTTCCGGAATCGTGGTCGGTCTGAACAAGGGCCACGTACGTTGAATCCCTCTCCCGTTTCCCTAAGAAAAAGAATGTTTCCCGTCGGTATTTGTCCAGTCGGTCTCGAGAAATTTGGATTGAGGATACGGAACATAAATACGGAGGGGACATATTGTTGGATTGTCAAAAGGCGCAATTTCTTATTGAGATGGTGGAAATGGAACGACAACGGACGTACCAATCGAATTTATTGGGCCCGACTTGACAGAAATTGACCGGAAGCATTTGACCTTGGTTTATAAGGAATAAGGAACAATCAGTGAAGCTGACTTGGGTTCCCATTGATAGAAAACCACCCCTCTGAACACCCCCAAGACCCGGGTCAGCCGCACCAAGGGCCAGTCTTCCCGCCGCACTGCCTTCGTCCGTGACATCGCCCGCGAGGTTGTCGGTCTTGCCCCTTATGAGCGTCGTATCATCGAACTTCTGAGAAACACTCAGGACAAGAGAGCTCGTAAGCTCGCCAAGAAGAGGGTATGTCACGAGTTCTGGATATTGGTCTTCACGGGTGTTCCTTTGCTCGCTTCCCTGGAATGTCAATCATACGGTGTCTGCTCTTCGAAGTACAATTTGATTAAGCATACGGCAACTGTCAATTGGACACTACGCCCTCCGTGAATCTAGCCATTCGAGTACAGACATCACCGACCAACGACTTGTGGAGCGAGCTTGGGGAACACTTTGTTGAGAACTTTCGCACTCATTCATAAACAATGCTAACGAATGATCTTTTATAGCTCGGTACCTTCTCCCGTGGCAAGAGAAAGGTTGAGGACATGCAGAGAGTCATCGCCGAGTCCAGACGTGTGGCTGGTCACTAA |
| L39 | - | BCLY01000012.1 (c1433760-1433236) | Met-loss | MPSHKSFRTKQKLAKAQRQNRPIPQWIRLRTGNTIRYNAKRRHWRKTRLGI | ATGCCGGTTAGCAGTCCTCCCCTACAATTCCGAGTCGTTTGAAGATGCCGGGACTTTATTTGTCCTGCTCTCGCATCGATGAACCGAGATATTCGTCTTGTGATGTTGATATGAGAGGGTGACAGTTGGCTAACGTTGCTTTGTGTTGAATAGAGCCACAAGAGTTTCCGCACCAAGCAGAAGCTTGCCAAAGCTCAGAGACAGAACCGTCCTATTCCCCAGTGGATTCGTCTCAGGACCGGTAACACCATCAGGTAAATATTCTCTTTTTCCTCTACAATTCGGAGCCGTCGATCGTGTGCAATTGTGGTGGGAGACTCGCTTGAATCGGACTTCGATCACAACGACAACCTTGCCATTTCCTGCATCAACACGCGCACCAAGGACATCACGCGAATCGAATTGGGACCTTGCGCGGGTTACATGGACCACAAAAGGAAGGAATGAATTAGCTGACGGGTACTCTTCTCATCCAGATACAACGCCAAGCGGAGACACTGGCGCAAGACCCGTCTCGGTATCTAA |
| L40 | - | BCLY01000016.1 (3802690-3803591) | N-terminal scission (Release ubiquitin with 76 amino acids at the N-terminal side) | IIEPSLKALASKYNCEKSICRKCYARLPPRATNCRKKKCGHTNQLRPKKKLK | AGTACGTACAGTATCATGTTCTCCTACGCTATATCTTTGATCTATGGATTACGAGCTAACATAACCTTTTTTCTGCGATTTTACAGTGCAGATGTAGGTCTTCCTGAGCCGTTCGGACCAACCCAGTGCCCATTTACCCAACCTCATCCCATCCATCGACTCGATATACGCGGAACATCCGGAAAAGCCATGACTGACAAATTTCGATCAATAGTTTCGTCAAGACCCTGACGGGTAAGACCATTACCCTCGACGTCGAGTCGAGCGACACCATCGACAACGTCAAGGCCAAGATCCAGGACAAGGAGGGTATCCCCCCTGACCAGCAGCGCCTGATCTTCGCCGGTAAGCAGCTTGAGGATGGCCGCACCCTGAGCGACTACAACATCCAGAAGGTACGCGAATTCTCTATCGGACGACGATTTGGTTTCGTTTCATTATGCGCATGGGATACTGTTGGATGATGCGGGTTTTTCGCTGGATTGCTGGACTTGGTTTCCGGATCCTCCGGCTGGAAGCGTCTGCGCTAGGGGATGACTGGACTGAATGGAAGATATACGGTTATTGAGAAGGATGTGTTTTGCTGACCGGATGCTTTTTGCACATATTTAGGAGTCCACTCTCCACCTGGTCCTCCGTCTGCGTGGTGGTATCATTGAACCCTCCCTTAAGGCTCTCGCTTCCAAGTACAACTGCGAGAAGTCCATCTGCCGCAAGTGCTACGTACGTCTTTGTCCCGATCGCTCGACCAGACTTGTGACCCATTACAGGGGACGCTCGCCTTGGAAGTCTTTGCTAACTCGATCCCTGGATAGGCCCGTCTTCCTCCCCGTGCCACCAACTGCCGTAAGAAGAAGTGCGGTCACACCAACCAGCTGCGCCCCAAGAAGAAGCTCAAGTAA |
| L42 | - | BCLY01000008.1 (2885120-2886069) | Met-loss, Methylation | MVNVPKTRRTYCKSKECHKHTQHKVTQYKAGKASLFAQGKRRYDRKQSGYGGQTKPVFHKKAKTTKKIVLRLECTACKAKKQLALKRCKHFELGGDKKTKGAALVF | ATGGTAAGAACATTTCTGCGTTATTCTTGCTCGCATGCTGTTTCCATCCTGGAGATTCGGAGAAGTTCTGTCGAAAACTCGGTACTTGGCGATGAAACGACATATTTGTTCTGGTTGAGGACGGAGAAGTTGCGGACAAAGGGAAAAACGGATGAAAATGCTATCGATCGCAACCACGATAAAATTCGCTCGGATATGAGAAATTGAACTATGTTCATGTGTCAGAGTTTAGAGGAGAAAATATGGGATATTCCTCGACGCGATGAGGACGCAACGGACGCCCGGATAAGAAAGACTCAGCGCATCAAACACGAAAAATAAGGACGAATAATCTCGCCCCAGAAGAACTTGTGACCTATAGTCTGCCGGGAATCTCAATCTCTTCCTATCACAACCGGTGCCGAGTTTGATAGGATTCTCCGATGACCGGGACATGGAAACAGGCAGCACCAAATAATTGTTGGATTATTGCAGATAGTTGCTGATAGCTCTTGCGCTCAGGTCAACGTTCCCAAAACCCGCCGGACGTACTGCAAGTCCAAGGAGTGCCACAAGCACACCCAGCACAAGGTCACCCAGTACAAGGCTGGCAAGGTGATTACAACCCGCACGCCATGGTAGACGAGTTGGCACAGGCACTGATGATGGATCTATTAGGCCTCCCTGTTCGCCCAGGGTAAGCGTCGTTACGACCGCAAGCAGAGCGGTTACGGTGGTCAGACCAAGCCTGTCTTCCACAAGAAGGCCAAGACCACCAAGAAGATCGTCCTGCGTCTTGAGTGCACTGCCTGCAAGGCCAAGAAGCAGCTCGCTCTGAAGCGTTGCAAGCACTTCGAGTTGGGGTACGTTGAGTCCGCGAATGTGGCAATACCTGATCCATGCATACTAACGTACGCTTTTTGCTGTTTAGTGGTGACAAGAAGACCAAGGGTGCTGCTCTTGTTTTCTAA |
| S27 | - | BCLY01000004.1 (2439583-2440087) | Met-loss,  Methylation (2) | MVLAVDLLNPTPQAEARKHKLKTLVPAPRSFFMDVKCPGCFTITTVFSHAQTVVVCAGCSTVLCQPTGGKARLTEGCSFRRK | ATGGTAGGTTCTGCTTGCAATTTGGCTTCATCCAACACCTTGGGATATCCCCTAGCCGTGCTGCTGGACGTCTGCACAGACTAGGAGCTTGCTGCCCGTCGTGTGCTCGCCGTCGCCGGACCGGTTCTCATCACACAAAGTCATCAAAGATCATCGGCTAACACAAGTTCTTTCTTTTGCAATAGGTTCTCGCGGTCGACCTCCTCAACCCTACTCCTCAGGCTGAGGCTCGCAAGCACAAGCTTAAGGTATGATACCGAAATACAAGTCCACCAAGGTCTCGGATCCGATGGCTGATGTTGGGAATCGCTTCGACAAATAGACCCTTGTGCCTGCTCCCCGTTCCTTCTTCATGGACGTCAAGTGCCCCGGCTGCTTCACCATCACCACCGTCTTCTCCCACGCCCAGACCGTCGTCGTCTGCGCCGGCTGCTCGACCGTTCTTTGCCAGCCCACCGGTGGCAAGGCCAGACTCACTGAGGGCTGCTCCTTCCGGAGGAAGTAA |
| S28 | - | BCLY01000005.1 (c1114006-1113431) | Acetylation | MDSAKQPVKLVKVTRVLGRTGSRGGVTQVRVEFMDDTSRSIIRNVKGPVKVDDILCLLESEREARRLR | ATGGATTCCGCCAAGCAGCCTGTTAAGCTCGTCAAGGTGACTCGTGTTCTCGGCCGTACCGGTACGTGATCTACAATCTACAATCTACAATGATGGATGTCGCGTTGGATCTGGTATAATAACTGCTTCTCGCTTTAGGCTCCCGTGGTGGTGTCACCCAGGTCCGCGTCGAGTTCATGGATGACACTTCCCGCAGCATCATCCGTAACGTCAAGGGTCCAGGTATGCGAGTTCCGTTGAGCGATTGAGTTTTGGGGGGTTTTCTTTCGGTGGTCCTCGTATCCCGGCCGACAATCCGATTGCGATGATTCAAAACGGAGGAAGAAACTGGTCATCTCGGTGCAAGAGCGGACAAAAGGAATTAACTGCGAATGACTGCAAATGCGAATGCGACTGCCAGCCCGAACCATATTGACTCCGGATACTGGGGACCATCGGAAGGGGAAAACCAGGGTCGAATATTGAAAAAGTGCATGTGCATGGGCTAACTAGGAATCTTTGTCGCAACCTACAGTCAAGGTCGATGACATCCTCTGCCTGCTCGAATCCGAGAGAGAGGCCCGCCGTCTGCGGTAA |
| S29 | - | BCLY01000001.1 (746116-746636) | Met-loss | MTHESVWYSRPRKFGKGSRECRVCAHRAGLIRKYGMDICRQCFREKAQDIGFYKYR | ATGACTCACGAGTCCGTTTGGTACAGCCGGCCCCGCAAGTAAGGATATTCTTCAATTCTGGGATGCATCGGTACGATTTGGCTAATGTGTTATCTCTCTATAGGTTCGGCAAGGGTTCCCGTGAATGGTGTGTGAACGAAATTCCCCAGGCGATACCAGGAGGATAGGATCGAAATATTATGGATTGCGAGCAGGAATGGCTAATCAGATATTTTGCGTCGAAACAGCCGTGTTTGCGCCCACCGCGCTGGTCTGATCCGCAAGTACGGGGTATGGAAATGAGCCAACTATCGAGAATAACCCGCGTTGGATATGCGACTGATAGTGGTTATAGATGGACATCTGCAGACAGTGCTTCCGTGAGAAGGCTCAGGACATCGGTTTCTACAAGGTTGGTTCCCACATATGCCGCAGAGCCAGAGCACCATGGCCAGGGTCAAACCGACGACTCTCTCGGAGAACTGGTTGGCAGAAGAAGAGCGGATCACTAACAGAACTACCTCTTCATACAGTACCGTTAA |
| S30 | - | BCLY01000001.1 (c3678203-3677625) | Met-loss | MGKVHGSLARAGKVKSATPKVEKQEKKKEPKGRALKRLKYTRRFVNVTMTGGKRKMNPNPTS | ATGGGTAAGGTTCACGGATCTCTCGCCCGTGCGGGTAAGGTCAAGTCTGCGACTCCTAAGGTGAGCTCTTCTTGTTCTTCCCTAGATGAAGTTTTGATCTCCGCGTTCCTCCTGGGAATGGTTCATCGTCGCCACCGAAGAGGAGGGATTTTGAGAAGATATTTGCAGCGGCCCGGCATGCAGAAATGGCCAAAATGATATATGCGAAAACGATATGATGAACCGATGGTTTTCCAGCGAGATATTTGCCGAATCAGAGTGCTGATGGGATATGTTCTACTGTACAGGTCGAGAAGCAAGAGAAGAAGAAGGAGCCCAAGGGCCGTGCTCTGAAGCGCCTCAAGTACACCCGCCGTTTCGTCAACGTTACCATGACTGGTGGCAAGCGGAAGGTACGTTGATATCCAGCTCGGTGGAAAGGATGCATATATGCTTTGTCGGGGGAGTTGGGAATTGTTCTCAGGAGAACCCCATTCTCCTCCGGCCAGCATATTTTGCAAGAGGTCTTTGGCATGAAAAAGGCAACAGGCACTGACAGGGCATTCTTTGTTACAGATGAACCCCAACCCCACTTCTTAA |
| S31 | A0A0S7DPQ3 |  | N-terminal scission (Release ubiquitin with 76 amino acids at the N-terminal side) | AKKRKKKVYTTPKKIKHKRKKTKLAVLKYYKVDGDGKIERLRRECPSPECGAGIFMAAMHNRQYCGKCHLTYVFDESK | ATGCAGATCTTCGTCAAGACCCTTACGGGTAAGACTATCACCCTCGAGGTGGAGTCTTCGGACACCATTGACAATGTCAAGTCCAAGATCCAGGGTACGCAACCCCTCCGAGCATCGTGAACCCGAGAAGAACTCTTAACCTGACTTGGTAAAACAGACAAGGAGGGAATCCCCCCCGACCAGCAGCGTCTGATCTTCGCTGGCAAGCAGCTCGAGGACGGCCGTACCCTCTCTGACTACAACATCCAGAAGGAGTCGACCCTGCACCTCGTGCTCCGCCTGCGTGGTGGTGCCAAGAAGCGCAAGAAGAAGGTCTACACCACCCCCAAGAAGATCAAGCACAAGCGCAAGAAGACCAAGCTCGCTGTCCTCAAGTACTACAAGGTTGACGGCGATGGCAAGATCGAGCGTCTCCGCCGCGAGTGCCCCTCCCCCGAGGTATGTTAACTTTCTCTCGTCTGAAAAGTGAGTCTCGAACTAACGAACTTGTAGTGCGGTGCTGGTATCTTCATGGCTGCTATGCACAACCGTCAGTACTGCGGCAAGTGCCACCTCACCTACGTCTTCGACGAGTCCAAGTAA |
| *A. viridinutans* IFM 47045^T^ | | | | | |
| L26 | - | LC213039 | Met-loss | MTVMNNGIASSRRKSRKAHFSAPSSERRVIMSAPLSKELREKYNVRAIPIRKDDEVTVVRGSNKGREGKITTVYRLKWCVHVERVVREKSNGQSVPIPIHPSKVVITKLKLDKDREQILERIGKGREAAKARA | ATGACCGTCATGAACAACGGTATGTTCGACTTATTTTGCTCAATTCACCCTCATGGCAACGTCCTGTTCCTCGAATACGCCTTTGATATTCTATTTTCTCGGAAGACACATTCTCAAAACCGCCTACGCTTAGGAATTGTGTTGGGAGGATACTGACTCTCGGTTTTTTCAATGACAGGAATCGCCTCTTCCCGCCGGAAGTCGCGCAAGGCGCACTTCAGCGCTCCCTCCAGCGAGCGCCGTGTCATCATGAGCGCTCCTCTGAGCAAGGAACTCCGTGAGAAGTACAACGTACGCCTCCCGTTATGCCACCTCTCCTAAACCGATCATCTCCCGTTATCAGATTCTACACTCTTGTTCGGAGGGACTCTTGCAGAAGCATAGCCAGGGATCACTGCAAAGAACTGTGAAGGATCTTCGTTCTGACAAATAACCTATAGGTTCGCGCCATCCCCATCCGCAAGGACGACGAGGTCACTGTTGTGCGCGGCTCCAACAAGGGACGCGAGGGAAAGATCACCACCGTCTACCGTCTCAAGTGGTGCGTTCACGTCGAGCGTGTCGTCCGTGAGAAGTCCAACGGCCAGAGCGTCCCTATCCCCATCCACCCCTCGAAGGTCGTTATCACCAAGCTCAAGCTGGACAAGGACCGCGAACAGATTCTGGAGCGCATCGGAAAGGGCCGTGAGGCTGCTAAGGCTCGTGCTTAA |
| L27 | - | LC213040 | - | MKFMKVGRVAIITRGRYAGKKVVIVQPNDTGSKAHPFPYAIVAGIERYPLKVTRRMGKKMVEKRSRIKPFIKVVNYNHLMPTRYTLELEGLKGAVSQETFKEVSTREDAKKTVKKALEDRYTSGKNRWFFTPLRF | ATGAAGTGTTAGTTGAATGCCGTTTCTTCTCGAGTTCTCAATTATTCGAATATCAGTTCATACTGACCGGATGGTGAAATAGTCATGAAAGTGGGCCGTGTGGCCATCATCACCCGTGGCCGTTACGCCGGTAAGAAGGTACGACAAGCCTTTTCTTTTTCCTACATGTCCTAGGGGGGAAATCTGGATGAATGATCTGGTTCGGAACTTTTCGATGGTCGATTTTGAGAGGGAGGGAGGGAGACGTTCTGGACGAATTGGGGGCGGGAGATCGATTTGGAATGGGATCAAGATTGGACATTGAGAAGGATGTGCTGACAAGGGACCTTTTCGCGAAATCACAGGTCGTCATTGTCCAGCCTAACGACACTGGCTCCAAGGCGCACCCCTTCCCCTACGCCATCGTCGCCGGTATCGAGCGCTACCCTCTCAAGGTCACCCGCCGCATGGGCAAGAAAATGGTCGAGAAGCGCAGCCGCATCAAGCCTTTCATCAAGGTCGTCAACTACAACCACTTGATGCCCACCCGTTACACTCTTGAGCTTGAGGGTCTCAAGGGTGCCGTCAGCCAGGAGACCTTCAAGGAAGTCTCCACACGCGAGGACGCCAAGAAGACCGTCAAGAAGGCCCTCGAGGACAGATACACCAGCGGCAAGAACAGATGGTTCTTCACTCCTCTGCGTACGTGCAATCCTTGCGTATTATCACTTGGAGTGAGATAGTTGTGTGCTGATTATGGTCTCTACACAGGTTTCTAA |
| L29 | - | LC213041 | Met-loss | MAKSKNASQHHRSQKAHRNGIKKPKTHRYPSLKGVDPKFRRNHRHALHGTMKALKERKEGKREVA | ATGGCCAGTACGTTACCTGAACTCCGGCGTTTTGGAATTTTCGAATCGATTTTATACCAGCACAATATCCCCGGATTGTTCGGTAGTCGCTGACCGTTATTTTCACTTGAACAGAGTCCAAGAACGCGTCTCAGCACCACCGCAGCCAGAAGGCTCACCGTAACGGGTTCGTACAGTCCTCAGCGATTTGAATCGAAATATTTTGGTCGGAAGGGAAGGATTGTTGCAGTGATGGATTTGGCATCACCGAATCGGTCCTGAATGACTCGTGACCATCTTGCAACATATCCACCAACCACATTTTGAAGAAGGAAAAGACAGGTCGCTGATATTTGCCGTTTGATTCCAACAGTATCAAGAAGCCTAAGACTCACCGTTACCCTTCCCTCAAGGGTGTTGACCCCAAGTTCCGCCGCAACCACAGACACGCCCTTCACGGCACCATGAAGGCTCTGGTACGTTGACCTTTTGCTGCTGCTGAGTCGGACGCAGCTGGATCCCGGAGGACATGGCTAACTATCCTACAGAAGGAGCGCAAGGAGGGCAAGCGGGAGGTCGCATAA |
| L30 | - | LC213042 | Met-loss | MAPKSKKSGDTISSRLALVMKSGKVTLGYKSTIKTLRSGKAKLVLIAANAPPLRKSELEYYAMLAKTPVHHFSGNNIELGTACGKLFRCSTMAILDAGDSDILSSQ | ATGGCCCCCAAGAGCAAGAAGTCCGGTGACACCATCTCCAGCCGTCTGGCTCTGGTGATGAAGTCTGGAAAGGGTGTGTGACACATTTCAATCAAATTGACGTCGAAGACCATATCACTGATTATTCTCTCCCCTCCAGTCACCCTTGGCTACAAGTCCACCATCAAGACCCTGCGTTCCGGCAAGGCCAAGCTGGTCCTCATTGCTGCCAATGCTCCTCCTCTCCGCAAGAGTGAGCTCGAGTACTACGCCATGCTGGCCAAGACTCCTGTCCACCACTTCTCTGGCAACAACGTAAGTGACACGTGTTTCTGCAATGGTTTTCACCGCTATGCCCCGATGCTGGTACTTCTCCAATGGAGGGAAGGCGACGCGGGTGCAGCCCTGAGAAGAAGCCGTATATATGGGCTTTGGAGGGTAAATGACAATGGCTGACATATATCCGTACTACAGATTGAGCTCGGTACCGCTTGCGGTAAGCTCTTCCGCTGCAGCACCATGGCCATCCTGGATGCTGGTGACTCCGACATCCTCAGCAGCCAGTAG |
| L31 | - | LC213043 | Met-loss, Acetylation | MSSTVKTTGKKTRSAIADVVTREYTINMHKRMHGVTFKKRAPRAIKEIRAFAERAMGTKDVRLDPQLNKKVWEAGVKGVPFRLRVRISRKRNDEEGAKERLYSYVQAVNVKDAKGLHTAVVDE | ATGTCCAGCACCGTCAAGACCACCGGCAAGAAGACTCGTTCGGCCATCGCCGACGTTGTCACCCGCGAGTACACCATCAACATGCACAAGAGAGTACGTTGAAGACGCTGGATCTTTGCTTTTGATTTCCTTTGCACACAATTTCGTGGTCAATGAATCGACCGGCTTTTTGCGACCTTGCGGAACCAAACACCCTGAGGCATGGACGGAGAACAACGTATGGGAACATCTCGAAACAACCTTGTGTGTGAGGAGGAATCCACTACAGAAGGACTTGTGCGAGATTTTTTTTTTTTGTCTTGGGGATCAGAATCCGAAACCCAAGGAAAGAGGAGGAACATACACAATACACGACAACAAAAAAAGAAGAAATAGTTTGATGAAGTCCACCTCTATGGCCGCAAATAGCATCTTCGCAGATTCACTCTACCAGATGGCATTTGGGCGGAAGACTAATTTCTCCTTTTTTTGCTCGATCTATAGATGCACGGTGTTACTTTCAAGAAGCGCGCTCCTCGTGCCATCAAGGAGATCCGCGCCTTTGCTGAGCGGGCTATGGTAAGTTTCTCTCCCATGAAGGTGATGGGATGGGGTCCAGCTCAGCTAACCACTCGCGTTCCGCCAATCAGGGCACCAAGGATGTCCGCCTCGACCCCCAGCTGAACAAGAAGGTCTGGGAAGCCGGTGTCAAGGGCGTTCCTTTCCGCCTCCGTGTCCGCATCTCCCGCAAGCGTAACGACGAGGAGGGCGCCAAGGAGAGACTCTACTCCTACGTCCAGGCCGTCAACGTCAAGGACGCCAAGGGTCTGCACACCGCTGTTGTTGACGAATAA |
| L32 | - | LC213044 | Met-loss | MVLAKKHVPIVKKRTKRFFRHQSDRFKCVPESWRKPKGIDNRVRRRFKGNIPMPSIGYGSNKKTKHMMPSGHKAFLVHNPKDVELLLMHNRTYAAEIASAVSSRKRVDIIAKAKALGVKVTNPKGRVTVEA | ATGGTCCTCGCAAAGAAGCACGTCCCTATCGTCAAGAAGCGTATGTTCTAGCTCCCTCTGCCAGCCTTTCCCTGCCTCAAGCACACGCACAAAATTCGGTTCTGGAGAGATATAAGGAATGGATGTTTTGGTTTGGATGTGATGGAAGAGACGGTTTCAGAGAAGAGATGGATCAGCGACCGGATCAAGACCTTTTGTTGCGAGCGTTGCATGAATTACTTGAAAATTCACACACACGAACACATGGAATATCAGGAGGACTGACACGGTTGTGAACAGGCACCAAGCGCTTCTTCCGCCACCAGTCCGACCGCTTCAAGTGCGTGCCGGAGTCATGGCGCAAGCCCAAGGGTATCGACAACCGTGTCCGCAGACGCTTCAAGGGCAACATCCCCATGCCTTCCGTACGTTCAGTCCCTCCGACCCTAAGCCGCCTCAGATTCGATCATCTCGACGAGAACAAATCTGGAATCGCTGTGGCCTACCTTTGCGAACTGCAATGCTGATATTTCCTGGACTAGATCGGTTACGGTAGCAACAAGAAGACCAAGCACATGATGCCCTCCGGCCACAAGGCTTTCCTCGTCCACAACCCCAAGGACGTCGAGCTGCTGCTCATGCACAACCGCACCTACGCCGCTGAGTACGTTGCATCCGTCTGAACAAAAGCAACACCATACTGATTGATGGAATAGGATCGCCTCCGCCGTCTCTTCCCGCAAGCGCGTCGACATCATCGCCAAGGCCAAGGCGCTCGGCGTCAAGGTCACCAACCCCAAGGGCCGTGTCACCGTTGAGGCGTAA |
| L33 | - | LC213045 | Met-loss | MPSEQGHRLYVKGRHLSYQRSKRAVNPNTSLIKIDGVDNTEAANFYLGKKVAFVYRAKREVRGSNIRVIWGKVTRPHGNSGVVRAQFRHNLPPKSFGATVRVMLYPSNI | ATGCCTTCGGAACAAGGTCACAGACGTGAGTGATCCCTCTCCGTTTAGCTTGATATCGATGTGTGCGCACAGGTTGGAGAAATGGGCTTCGGGGATGGATGCGGTGCGATTATGAAATTAATAAAGGATCATGCTAACGGGATATTTCTACTTGCACAGTCTACGTCAAGTAAGTTACCACGATCTTCTCATCGATTCTCCGTCGGGTCGCGATCATTCGGAATGCGATATATACAAATGTCAAATGTCGCACTGTGGGATCGGGCTAGGGGGCCAAGTCGAAAGGAAATCATACGGACAGATGGGTTAATACGAATTCTTTATGCAGGGGTCGCCACCTGAGCTACCAGCGCTCCAAGCGCGCGGTCAACCCCAACACCAGGTAGCTATCCACTGTGAAGCTGGACGTCAAGGGATATGGGAGGCTGACACCGTGTATAGTCTGATCAAGATCGACGGTGTTGACAACACTGAGGCCGCAAAGTGAGTATACACTGGTCGAAAGGGTCACGACTTAAGAAGAGAACATGCAGCTAAGACGGTTGTTTTACAGCTTCTACCTCGGCAAGAAGGTCGCTTTCGTTTACCGGGCAAAGCGTGAGGTTCGGGGTTCCAACATCCGGGTCATCTGGGGCAAGGTTACACGGCCACACGGTACGTGATTTCAATGGCGATGGATCGGTGATATTGGCATCGCAGCCCTGTTGGACGATGGATGGCTGCTAGGAGGAACAACAACTGACGATATCAACTTTACAGGCAACTCCGGCGTTGTCCGTGCTCAGTTCCGCCACAACCTCCCCCCCAAGTCCTTCGGTGCTACCGTCCGCGTCATGCTCTACCCCTCCAACATCTAA |
| L34 | - | LC213046 | Met-loss | MANNRLQYRRRNPYNTRSNKVRIIKTPGGELRYLHIKKKGTAPKCGDCGIKLPGIPALRPREYSQISRPKKTVSRAYGGSRCAGCVKDRIVRAFLIEEQKIVKKVLKESQEKAAGKR | ATGGCGAACAACAGATTGCAATACGTGAGTTTTTGAACTGGAATATATTTCTTGCTTGGATGAAATACTGATTTTCTTTTTTTTTTTCTACAGCGGAGACGGAACCCGTACGTTTGAAATTCCCTCCCAGAAGTTCACAATACTATGCCCCCTGCGTCGCTTTCATTATCGGCGTTGTTCGCTTCAGTTCAGTGGCATATCGCATGATATTTGGCGCGACGAGACGCATCGGTTCTGAATCTGCCCTTGAAAGCACGATATTGATAGGTTTTTGTTCTACAGGTACAACACGCGGTCCAACAAGGTCCGCATCATCAAGACTCCTGGCGGCGAGCTCCGTTACCTCCACATCAAGAAGAAGGGCACTGCTCCCAAGTGCGGTGACTGTGGCATCAAGCTCCCCGGCGTGAGTGCTCCCGAGTGAAATTCTGGCTAGCAAGTGGTCTTCTGTGCGCTTTTTTGGATGTCTTCGATTGTACTGTCGAGTCGAGAGAGACAAGCTCGAGGAACGGAAAACGCACATTTTGATCACCTCTAGCCAAGTGATAGGAAGATGAGCAGGATCGCTGATTAGAAGGCTTAGATTCCCGCCCTCCGCCCTCGTGAATACTCCCAGATCTCCCGGCCCAAGAAGACCGTCAGCCGTGCCTATGGTGGTTCTCGCTGCGCCGGTTGCGTCAAGGACCGTATTGTCCGTGCTTTCCTGATTGAGGAGCAGAAGATCGTCAAGAAGGTCCTCAAGGAGTCTCAGGAGAAGGCCGCTGGCAAGCGCTAA |
| L36 | - | LC213047 | Met-loss | MAQERSGIVVGLNKGHKTTPLNTPKTRISRTKGQSSRRTAFVRDIAREVVGLAPYERRIIELLRNTQDKRARKLAKKRLGTFSRGKRKVEDMQRVIAESRRVAGH | ATGGCGCAGGAACGTTCCGGAATCGTGGTCGGTCTGAACAAGGGCCACGTACGTTGAACCCCTCTCCCGTTTCCCTAAGAAAAAGAATGTTTCCGGTCGGTGTTTGTCCAGTCGATCTCGAGAGATTTGGATTGAGGATGCGGAGCATAAAATATGGAGGGGATATATTGTCGGATTGTCCAAAGGCTCAGTTCCTTCTTGAGATGGAGGAAATGGAACGACAACGGACGTACCAATCGAACTTATTGAGCCCGACTTGACACAAATCGATCGGAAACATTCGACTTTGGTTATATAGGAATGAGGAACAATCAGTGAAGCTGACTTGGGTTCCCATGATAGAAAACCACCCCTCTCAACACCCCCAAGACCCGGATCAGCCGCACCAAGGGCCAGTCTTCCCGCCGCACTGCCTTCGTCCGTGACATCGCCCGCGAGGTTGTCGGTCTTGCCCCCTATGAGCGTCGTATCATCGAACTTCTGAGAAACACTCAGGACAAGAGAGCTCGTAAGCTCGCCAAGAAGAGGGTATGTCATGAGTTCTGGATATTGGTCTTCATGGGTGTTCCTCTCGCTCGCTCCCCTGGTAACTGCATGATTGCGGTGTCTGCGGTTCGGAGTACAATCTGATGAAGCATACGGCAATTGTCGGTTGGACACTACGCCCGCCACGAATCTGTCCCTTCAATTACTGAGACCACCGACCAACGGCCTGTGGAGCGAGTTGAGGGAACACTTGCTGAGAACTTTAGCAGTCGTTCATGAACAATGCTAACGAATCTTCTGTTATAGCTCGGTACCTTCTCCCGTGGCAAGAGAAAGGTTGAGGACATGCAGAGAGTCATCGCCGAGTCCAGACGTGTGGCTGGTCACTAA |
| L37 | - | LC213048 | Met-loss | MTKGTSSFGKRHNKSHTLCRRCGKRSFHIQKSTCANCGYPSAKVRKYNWSEKAKRRKTTGTGRMSHLKDVHRRFKNGFQVGTPKGARGPEIH | ATGAGTACGTACACATTCGACATTTCACCGAGCGCCGATAGCCTTTCTCGTCGAAAGATTCTAGCAGACTGACAACGATTACAGCGAAGGGTACCTCCAGCTTCGGCAAGCGCCACAACAAGTCTCACACCCTCTGCCGGCGTTGTGGTCAGTATTTCACCTTTCGAGATTGTTCCGGTGGAAGATTCATGCGGATCTCTATATGGGAGACGGTGTTGGGATATTGCCGGAGGAAAACGATAGGATTCAGATGGGCTAACACCTTGTGATTTTCGATTGCAGGCAAACGCTCCTTCCACATCCAGAAGTCGACCTGTGCCAACTGCGGTTACCCCTCCGCTAAGGTTCGCAAGTGTACGGATTCCCCGATTGTCGTTATTTCTTTTGTTTGAACCCGTGCTTGGTCGACAATGGAACCCTTTCAATCACGGCTGTGTGGTTGAGTTTTGGAATGTTGGCTTACAGATTTTCTTTTAACAACAGACAACTGGAGCGAGAAGGCCAAGAGAAGAAAGACCACCGGCACCGGCAGAATGAGCCACCTCAAGGATGTTCACCGTCGCTTCAAGAACGGCTTCCAGGTCGGCACTCCCAAGGGCGCCCGTGGTCCCGAAATCCACTAA |
| L38 | - | LC213049 | Met-loss | MPREVSDIKQFIEICRRKDASSARIKRNRSTQQIKFKVRCHRFIYTLVLKDSDKADKLKQSLPPALKVVDVSKGDKKKAL | ATGCCTCGCGAAGTTTCCGATATCAAGCAGTTCATCGAGATCTGCCGCCGCAAGGATGCCTCCTGTACGTTTTCCGCACGCGATTGCCCCGGTTTTAGAGCGACGTGAATGAGCTCGGATTCAGCTCCGTGGAGGGATTAGCGACGGAGTTGCATGAATGCTAACAATTGGTTTTCTTTCATTACAGCTGCCCGCATCAAGCGCAACCGCTCGACCCAGCAGATCAAGTTCAAGGTCAGATGCCACCGCTTCATCTACACCCTTGTCCTGAAGGACTCCGACAAGGCCGACAAGCTCAAGCAGAGCCTGCCCCCAGGTGCGTTACCAACCTAAGTTTCTATCCGAATTTCCCCAGTCACGGATCACTGCTTTTGGAAATAGTGTCTAGTGGTAGAAGTGATGAGTGGAATGAGAAGAGAAGCCGCGTTTCTGCTCGGGATCTTAAGCTAACGCTACGTCTTCCAACAGCTCTCAAGGTTGTCGATGTGTCCAAGGGTGACAAGAAGAAGGCTCTGTAA |
| L39 | - | LC213050 | Met-loss | MPSHKSFRTKQKLAKAQRQNRPIPQWIRLRTGNTIRYNAKRRHWRKTRLGI | ATGCCGGTTAGCAGTCCTCCCCTACGATTCCGAGTTATTTGAAGATGCTGGAACTTTTATTTATCTTGCTCTCGCATCGATGAACCGAGATATTCGTCTTTGATGTTGAGGTGACAAGGTGACAGTTGGCTAACGTCGATTTTTGTTGAATAGAGCCACAAGAGTTTCCGCACCAAGCAGAAGCTTGCCAAAGCTCAGAGACAGAACCGTCCTATTCCCCAGTGGATTCGTCTCAGGACTGGTAACACCATCAGGTAAATACACTCTTTTTCTTCTACAATCCGGAGCCGTCCATCGTAGGCAATGGTGGTGGGAAATTCCTTGAATCGGACCTCGATCACAACGACAAACTTGTCATCCCGCATCAACAATCGCCCCAGGAAAATCACACGAATGGATTTGGAACATTGCGCGGGGGTTACATGGACATAAAAGGAAGAATGAACCAGCTGACGGGTACTCTTCTCCTCCAGATACAACGCCAAGCGGAGACACTGGCGCAAGACCCGTCTCGGTATCTAA |
| L40 | - | LC213051 | N-terminal scission (Release ubiquitin with 76 amino acids at the N-terminal side) | IIEPSLKALASKYNCEKSICRKCYARLPPRATNCRKKKCGHTNQLRPKKKLK | AGTACGTACAGCATCATATTCTCCTACGCTATATTTTTGGTCTATAGATTACGAGCTAACATGATCTTTTTTCTGCGATTTTACAGTGCAGATGTAGGTCTTCCAGAGCCGTTCGGACCAACCCAGTGCCTATTTGCCCAACCTCATCCCATTCATAGACTCGATCTACGCGCAACATCCGGAAAAGCCATGACTGACAAGTTTCGATCAATAGTTTCGTCAAGACCCTGACGGGTAAGACCATTACCCTCGACGTTGAGTCGAGCGACACCATCGACAACGTCAAGGCCAAGATCCAGGACAAGGAGGGTATCCCCCCTGACCAGCAGCGCCTGATCTTCGCTGGTAAGCAGCTTGAGGATGGCCGCACCCTGAGCGACTACAACATCCAGAAGGTACGCGAATTCTCTATCGGACGATGCTTGGGTTTCGTTTCATTATGCGCATGGGGTTTTGTCGGATGATGCGGGTTGTTCGCTGGATTGCTGGACTTGGTTTCCGGATCCTCTGGCAGGGAACGTCTGCGCTAGGAGATGATTGGATTGTTACGGGGGATATATGGTTATTGAGAAGGATATGTTGGGCTGACCGGACTTTTGCACATATTTAGGAGTCCACTCTCCACCTCGTCCTCCGTCTGCGTGGTGGTATCATTGAGCCCTCCCTCAAGGCTCTCGCTTCCAAGTACAACTGCGAGAAGTCCATCTGCCGCAAGTGCTACGTACGTCCTTGTCCCGATCGCTCGACCCGACCTGTGAACCATTACAGGGGACGCTCGCCTTGAAGTCTTTGCTAACTTGATCCCTGGATAGGCCCGTCTTCCTCCCCGTGCCACCAACTGCCGTAAGAAGAAGTGCGGTCACACCAACCAGCTGCGCCCCAAGAAGAAGCTCAAGTAA |
| L42 | - | LC213052 | Met-loss, Methylation | MVNVPKTRRTYCKSKECHKHTQHKVTQYKAGKASLFAQGKRRYDRKQSGYGGQTKPVFHKKAKTTKKVVLRLECTACKTKKQLALKRCKHFELGGDKKTKGAALVF | ATGGTAAGGACTATTTTGCGTTTTCCTTGCTCGCATGCTGTTTCCATCCCGGAGATTCGGAGAAGTTCTGTCGAATACTCGGTACTTGGCGATGAGACGAGATGTTTGTTCCGGGTCGAGGACGAAGAAGTTGCGGACAACGGTAAAAACGGATGAAAATGCTATCGATCGCAAACCACGATAAATTTCGCTCGGATATGAGGCATTGAACTGTTTTCATGCGTCAGAGTTCAGAGGAGAAATTATGGGAAATTCCTTGAAGCGATGAGACGCAACGAACGCCTGGACGACGAAAGACCAGCGCATCGAATACGAAAAAAAGGACGAATATTCTCGCCAGAAGAACTTGTGACCTAGTCTCTGCCAGGAATCTGAATCCCTTCCCATGACAACCGGTGCTGAGTCTGATGGGGTTCTCCGATGACCGGGACATGGAAACACGCAGCACCAAGGAGTTTTTGGATTGTAGCGGACAGTTGCTGATAGCTCTTGCGCTCCAGGTCAACGTTCCCAAAACCCGCCGGACGTACTGCAAGTCCAAGGAGTGCCACAAGCACACCCAGCACAAGGTCACCCAGTACAAGGCTGGCAAGGTGAATACAACCCACACGCCATGGTAGACGAGTTGGCACAGGCACTGATGATGGATCTATTAGGCCTCCCTGTTCGCCCAGGGTAAGCGTCGTTACGACCGCAAGCAGAGCGGTTACGGTGGTCAGACCAAGCCTGTCTTCCACAAGAAGGCCAAGACCACCAAGAAGGTCGTCCTGCGTCTTGAGTGCACTGCCTGCAAGACCAAGAAGCAGCTCGCTCTGAAGCGTTGCAAGCACTTCGAGTTGGGGTACGTTGAGTCTGCGACTGTGGCGATATCTGGTTTATACATACTAACGTGCGTTTTTCGCTGTTTAGTGGTGACAAGAAGACCAAGGGTGCTGCTCTTGTTTTCTAA |
| L43 | - | LC213053 | Met-loss | MTKRTKKVGITGKYGTRYGASLRKQVKKMEITQHARYVCTFCGKNTVKRQAVGIWECKGCKKTVAGGAYTVSTPAAAATRSTIRRLREIAEV | ATGACGAAGCGCACTAAGAGTGAGTGATTTTGATCGAGATAAACCACAGGCACAGGATGGAAAAAAGGATAGAAATACTGACTGCATACTTTTAGAGGTCGGTATCACGGGTAAATATGGTACCAGGTATGCTTTTCGCTCTCGCACCTCGAAATCGATCTGAAGTCGTCTGCGTTCCGCTCTGCTCAATCGAAACCGGAATCGAAAGCGTGGAATATCGGATTCAGGAGAGCTAGAATGGTTGGTTGACATGGCTTGTTCATTACAGATACGGTGCCTCCCTGCGTAAGCAGGTGAAGAAGATGGAAATTACCCAGCACGCCCGCTACGTCTGCACCTTCTGCGGAAAGAACACCGTCAAGCGCCAGGCTGTTGGTATCTGGGAGTGCAAGGGCTGCAAGAAGACCGTTGCCGGTGGTGCCTACACCGTCTCGTAAGTCGACCACACGTTTTTCTCCCTTGTTCCCAGCCGTCTCGATCGCCACGCCTTGGAACAGGCCAGACTGGTGAAAGAAAAGCCTGGAGTCTTCACGGTGACACGGATACTGACCGATGTGACTGTACTACAGGACACCCGCCGCCGCTGCCACCCGCTCGACCATCCGTCGTCTCAGAGAAATCGCGGAGGTTTAA |
| S16 | - | LC213054 | Met-loss, Acetylation | MASVPSVQCFGKKKTATAVAHCKQGKGLIKVNGQPLGLVQPEILRFKVYEPLLIVGADKFAGVDIRVRVSGGGHTSQIYAIRQAIAKSIVAYYQKYVDEHSKNQLKQAFVQYDRTLLVADNRRTEPKKFGGRGARARYQKSYR | ATGGCTTCCGTCCCGAGTGTGCAATGCTTCGGCAAGAAGAAGACGGGTAAGTTTTTCAGAAGGGACAGAACTCGGTGATGAAGAAAACGTGATCACTGACTGGGAATTATCTGTATAGCCACCGCTGTCGCCCACTGCAAGGTATGAGGAGAAATTTTCCCCATTTCCCTATGATGTCGCCCCATTTGGCAAACTGCGTGATTTATCGAGTTGGAATGTCCGGAACATGAAAAAGAGAAGGGTTGATTTGGAGGATAAAAATGGAGGATCCGCTTTTGCATTCGCATGGAAACAACAGAACAAATGCTGAGAATTGTGTTATCAACAGCAAGGCAAGGGTCTCATCAAGGTCAACGGCCAGCCTCTCGGCCTGGTCCAGCCTGAGATCCTCCGCTTCAAGGTACGACTACAAAACCCATCCGAAAGGGTCGCATTGTTTTGCGAAGGAAAGAGGACAAAAAGGAAAAACAGAATCTGACGACAATGCAAATAGGTCTACGAGCCTCTCCTGATCGTCGGCGCTGACAAGTTCGCCGGTGTCGACATCCGTGTCCGTGTCTCCGGTGGTGGTCACACCTCCCAGATCTACGCCATCCGCCAGGCCATCGCCAAGTCTATCGTCGCCTACTACCAGAAGTACGTCGATGAGCACTCCAAGAACCAGCTGAAGCAGGCTTTTGTTCAGTACGACCGCACACTGCTCGTCGCCGACAACCGTCGTACGGAGCCCAAGAAGTTCGGTGGTCGCGGTGCCCGTGCCAGGTACCAGAAATCCTACCGTTAA |
| S21 | - | LC213055 | Acetylation | MENEKGEIVDLYVPRKCSATNRIIKANDHASVQISIGKVDENGRYTGENQSYALCGFIRARGESDDSLNRLCQRDGYVRNVWAASRQR | ATGGAGAACGAAAAGGGAGAGATCGTCGATCTGTACGTGAATGCTAATTTCCTTCGCTGTTTGCGCTTGAACTGGTTTTACGGTGCTCCGGGAGCCGAGAATGACTTTTGCGACTTGGGACAACACACAAGAGGACAAGAGGACAAGAGGACAATCTGGAGCGAGCTTTCAGCCTTCAACAGGCGCTGTTGCAGGATAGTCCCTTTTCGCAGAGTTCTTCCCGGCTTCCGGAAATCAGCAGGAGCAGGGAGATAATCGTGAACCAAAAGCATTGAATATTGCAAAACGCACATCACTAACTTCTGTTACTTTTCCTACAGCTACGTCCCCCGCAAGTGCAGCGCCACCAACCGCATCATCAAGGCCAACGACCACGCCTCCGTCCAGATCTCCATCGGCAAGGTCGACGAGAACGGTCGCTACACCGGCGAGAACCAGAGCTACGCTCTCTGCGGTTTCATCCGTGCCCGTGGCGAGAGCGATGACTCCCTGAACCGCCTCTGCCAGCGTGATGGCTACGTTCGCAACGTCTGGGCCGCCAGCCGCCAGCGGTAA |
| S23 | - | LC213056 | Met-loss, Hydroxylation (2) | MGKGKPRGLNAARKLANHRREQRWADLHYKKRLLGTAFKSSPFGGASHAKGIVLEKVGVEAKQPNSAIRKCVKVQLIKNGKKVTAFVPNDGCLNFIDENDEVLLAGFGRKGKAKGDIPGVRFKVVKVSGVGLLALWKEKKEKPRS | ATGGGTAAGGGAAAGCCCCGTGGTTTGAACGCCGCGCGCAAGCTCGCCAACCACCGCCGTGAGCAGCGCTGGGCCGACTTGCACTACAAGAAGCGCCTTCTCGGTACCGCTTTCAAGTCCTCTCCCTTCGGTGGTGCTTCCCACGCCAAGGGTATCGTCCTCGAAAAGGTCGGAGTTGAGGCCAAGCAGCCCAACTCTGCCATTCGGAAGTGTGTCAAGGTCCAGCTGATCAAGAACGGCAAGAAGGTCACCGCTTTCGGTGGGTAACCCTTTTCTATCAATTATGCACATTCGATAAGCTTCTGGGGATGGAGAAGAGACGGAATATTTGATGGATGCCACGACAGATTGGGAAAGTCCAGAATCGGCTTTGGATGGGGAGATGTGGAAGAGAAGCAGAGAAATTATGGTGGCAAATGCTCAAATGCTGACTTGACGATCTCTACAGTCCCCAACGACGGTTGCTTGAACTTCATCGACGAGAACGACGAGGTTCTCCTTGCTGGTTTCGGTCGTAAGGGCAAGGCCAAGGGTGATATTCCCGGTGTTCGTTTCAAGGTCGTCAAGGTCTCTGGTGTCGGTCTGCTCGCTCTGTGGAAGGAGAAGAAGGAGAAGCCCCGTTCGTAA |
| S24 | - | LC213057 | Met-loss, Acetylation | MADTPVTLRTRKFIRNPLLARKQMVVDVLHPNRANVSKDELREKLAELYKANKDQVSVFGFRTQYGGGKSTGFALVYDSHEALKKFEPHYRLVRVGAATKIEKASRQQRKQRKNRSKKFRGTAKTKGPKKNKD | ATGGCCGACACTCCCGTTACCCTGCGGACTCGCAAGTTCATCCGCAACCCCCTGCTCGCCAGAAAGCAGATGGTCGTGTAAGGAGCCCTTTTCTTCCTATCACGCGCTTGAGAATTTCCAGGGAGAGGGTCCCTTCTGGTCAAGATTTTTTTTTTTTTGAAAGTGATTTTGAGTGTGGAACACGCGGAACGGCAAAAGATGGAAGGCAACAAATGGCGATGACAATGGAGATAATCAGTTCATTTATCACCTTGGAATACTATCATGGAGGAAGGACCTGGAAATTCTCAAAGCACAGATGCAAAGGACATTCATTGTCGTGATGGTGATGATCAGTTTGATGATGATTCTAACATCGACTTCTTGCAGGGACGTTCTCCACCCCAACCGTGCTAATGTCTCCAAGGACGAGCTCCGCGAGAAGCTCGCCGAGCTGTACAAGGCCAACAAGGACCAGGTCTCCGTCTTTGGTTTCCGCACTCAGTACGGTGGTGGCAAGAGCACTGGCTTCGCCCTCGTCTACGACTCCCACGAGGCCCTGAAGAAGTTCGAGCCTCACTACCGCCTGGTCCGCGTCGGTGCCGCTACTAAGATCGAGAAGGCCAGCAGACAGCAGCGTACGTTTGAGCCACCGCCCACTCGCACCCTCGAGATTGAATCAGACATGCATACATGCCTTTCATATCTCCGGGGGTTACTCAGTCTAGCATCGCTAATCTCTCTTCCCACTTTATAGGCAAGCAACGGAAGAACCGTTCCAAGAAGTTCCGCGGTACCGCCAAGACCAAGGGACCCAAGAAGAACAAGGACTAA |
| S26 | - | LC213058 | Met-loss | MVKKRANNGRNKNGRGHVKPVRCSNCARCTPKDKAIKRFTIRNMVESAAIRDISDASVFTDYAVPKMYLKLQYCVSCAIHGKIVRVRSREGRRNRAPPPRIRYNKDGKKLAPPQAAKAM | ATGGTCAAGAAGAGGGCGAACAAGTAAGAGCGAATTTCCCTTTTCCTGTGTTCAACCCGGTTGTCAAATTTGAAGATCCATCACCGCCGGAAGAAGCGCCCTGATTTCCCGCCTGTCGAGGATGTCGGCTGTGTATGCGGTGGTTTTTATGATGGAATTGAAAGAGGAGGGGAAAATTTGGACTTCAAATTCATGAGTGCATCGATCCATACTGACATTTATTGTTCTTTCTCTACAGCGGTCGCAACAAGAATGGCCGCGGCCACGTCAAGCCCGTGCGCTGCTCCAACTGCGCTCGTTGCACTCCTAAGGACAAGGCGATCAAGAGATTCACCATCCGCAACATGGTTGAGTCCGCTGCCATCCGTACGTTCGAACCCTTGCCAAACCCATACCATTGAAGCCACGTCCGTAGTGCGCCGTTGAGATGATTCGGAATTGGATTGGACGGATGGAATCTCCTGGACGGAGCTGCCACAACTGGACGCATGAGCTGGGCGTGGTGGAATATGAAAGCTGATGGACAACGCCGCTGACCCGATACCTTGAACAGGTGATATCTCTGACGCCTCCGTTTTCACCGACTATGCCGTCCCTAAGATGTACCTGAAGCTGCAGTACTGCGTCTCCTGCGCTATCCACGGCAAGATCGTTCGGTACGCACCGTCCCGTTTTTGAATACAGCTTGGAATCTACTTGGTTTTGCAGCATGAAGGAAAGAATCAGGCCTTACTAACAATTCTCCACCACAGTGTCCGCTCCCGGGAAGGTCGTCGTAACCGTGCTCCTCCCCCGCGTATCAGGTACAACAAGGACGGCAAGAAGCTGGCTCCCCCTCAGGCCGCCAAGGCTATGTAA |
| S27 |  | LC213059 | Met-loss | MVLAVDLLNPTPQAEARKHKLKTLVPAPRSFFMDVKCPGCFTITTVFSHAQTVVVCAGCSTVLCQPTGGKARLTEGCSFRRK | ATGGTAGGTTCTACTTGCATTTTAGCTTCATGCAACACCTTGGGATATCCCCTAGCCGTTCTGCTTGTCGTCTGCACAGGCTAAGGAGCTTGCTGCCCGTCGTGTGCACGCCTGTCGCCGGACCGTTTCTCATCTTCATCGCAAAAAGACATCAAAGTATCAATGACTAACACAATTTTTCTCTTTCACAATAGGTTCTCGCGGTCGACCTCCTCAACCCTACTCCTCAGGCTGAGGCTCGCAAGCACAAGCTTAAGGTATGATACCGAAATACATGTCCACCAAGGTCCCGGATCCGATGGCTGATGTTGGGAATCGCTTCGACAAATAGACCCTTGTGCCTGCTCCCCGTTCCTTCTTCATGGACGTCAAGTGCCCCGGCTGCTTCACCATCACCACCGTCTTCTCCCACGCCCAGACCGTCGTCGTCTGCGCCGGCTGCTCGACCGTCCTTTGCCAGCCCACCGGTGGCAAGGCCAGACTTACTGAGGGCTGCTCCTTCCGGAGGAAGTAA |
| S28 | - | LC213060 | Acetylation | MDSAKQPVKLVKVTRVLGRTGSRGGVTQVRVEFMDDTSRSIIRNVKGPVKVDDILCLLESEREARRLR | ATGGATTCCGCCAAGCAGCCTGTTAAGCTCGTCAAGGTGACTCGTGTTCTCGGCCGTACCGGTACGTGATCTACAATCTACAAACAATGTTGGAAGTCGCGTGGAGTTTGGTATAATAACTGCTCGCTTTAGGCTCCCGTGGTGGTGTCACCCAGGTCCGCGTCGAGTTCATGGATGACACCTCCCGCAGCATCATCCGTAACGTCAAGGGTCCAGGTATGCGATTTCCGTTCAGCGATTGAGTTTTGGAGGTTTTTCTTTCGGTGGTCCTCGTATCCCGGCCGACAATCCGATTGCGACCATTCGAGACGGAGGAAGAAACTGGTCATCTCGGTGCAAGAGCGAACGAAAAAGGAATGACTGCAAATACGGATGCGACTGCAAGCCCGAACCATATTGAATCCGGATACCGGGGACCATCGGAAGGGGAAGACCAGGGCCGAATATTCAAAAAGTGCATGGGCTAACTAGGAATCTTTGTCGCAACCTACAGTCAAGGTCGATGACATCCTCTGCCTGCTCGAATCCGAGAGAGAGGCCCGCCGTCTGCGGTAA |
| S29 | - | LC213061 | Met-loss | MTHESVWYSRPRKFGKGSRECRVCAHRAGLIRKYGMDICRQCFREKAQDIGFYKYR | ATGACTCACGAGTCCGTTTGGTACAGCCGGCCCCGCAAGTAAGGATATTCTTCTATTCTAGGATGCGTCGATACGATTTGGCTAATTACGCTTTTCCCTCGATAGGTTCGGCAAGGGTTCCCGTGAATGGTGTGTGAACGAAAATCCCCAGGCGATACCAGGAGGATAGGTCGAAATTTCATGGATTGCGAGCAGGAATGGCTAATCAGATATTTGCGTCGAAACAGCCGTGTTTGCGCCCACCGCGCTGGTCTCATCCGCAAGTACGGGGTATGGAAATGAGCCAACTATCGAGAATAACCAGCATTGGATATGCGACTGATTGTGGTTATAGATGGACATCTGTAGACAGTGCTTCCGTGAGAAGGCTCAGGACATCGGTTTCTACAAGGTTGGTTCCCACATACGCCGCAGAGTCGGAACACCATGGCCAGGTTCAACCCGACGACACTCTCGGAGAACTGGTTGGCAGAAGAAGAGCGGATCACTAACAGATCACCTCTACCATCCAGTACCGTTAA |
| S30 | - | LC213062 | Met-loss | MGKVHGSLARAGKVKSATPKVEKQEKKKEPKGRALKRVKYTRRFVNVTMTGGKRKMNPNPTS | ATGGGTAAGGTTCACGGCTCTCTCGCCCGTGCGGGTAAGGTCAAGTCTGCGACTCCTAAGGTGAGTTCTTCTTGTTCTTCCTTAAATGAAGTTTTGATCTCCGCGGTCCTGCTGGGAAGGGTTCATCGTCGCCACCGGAAGAGGAGGGATTTTGAGGGAGGTTTTTGCAGCGGTTCGACATGCAGAAATGGCAGAGAGGATATATGCGAAGACGATTTGATGAACCGATGGCTTTCCAGGGAGATATTTGCTGAGTCAGAGTGCTGATGGGTTATGTTCTACTTTACAGGTCGAGAAGCAAGAGAAGAAGAAGGAGCCCAAGGGCCGTGCTCTGAAGCGCGTCAAGTACACCCGCCGTTTCGTCAACGTTACCATGACCGGTGGCAAGCGGAAGGTACGTTGATATCCAGCTCGATGGAAAGGATGCATATATGCTCTTGGCGGGGAAGTTGGTGGTTTTTTTTTTCGAGAGAGAACCCCCTTTCTCCGTCTGGCCGGCAGATTTTGCAGGAGGTCCATGGCATCGAAAAGGCATCAGACACTGACAAGGACATTCGTTACAGATGAACCCCAACCCCACTTCCTAA |
| S31 | - | LC213063 | N-terminal scission (Release ubiquitin with 76 amino acids at the N-terminal side) | AKKRKKKVYTTPKKIKHKRKKTKLAVLKYYKVDGDGKIERLRRECPSPECGAGIFMAAMHNRQYCGKCHLTYVFDESK | ATGCAGATCTTCGTCAAGACCCTTACGGGTAAGACTATCACCCTCGAGGTGGAGTCTTCGGACACCATTGACAATGTCAAGTCCAAGATCCAGGGTACGCAACCCCTCCGAGCACAATGACTCGAGAGACTCTTAATCTGACCTGGTAACAGACAAGGAGGGAATCCCCCCCGACCAGCAGCGTCTGATCTTCGCTGGCAAGCAGCTCGAGGACGGCCGTACCCTCTCTGACTACAACATCCAGAAGGAGTCGACCCTGCACCTCGTGCTCCGCCTGCGTGGTGGTGCCAAGAAGCGCAAGAAGAAGGTCTACACCACCCCCAAGAAGATCAAGCACAAGCGCAAGAAGACCAAGCTCGCTGTCCTCAAGTACTACAAGGTTGACGGCGATGGCAAGATCGAGCGTCTCCGCCGCGAGTGCCCCTCCCCCGAGGTATGTCAACTTACTTTCTGAAAAGCAACAGCTCGCAGCTAACGAATTTGTAGTGCGGTGCTGGTATCTTCATGGCTGCTATGCACAACCGTCAGTACTGCGGCAAGTGCCACCTCACCTACGTCTTTGACGAGTCCAAGTAA |
| *A. udagawae* IFM 46973^T^ | | | | | |
| L26 | A0A0K8LIR5 |  | Met-loss | MTVMNNGIASSRRKSRKAHFSAPSSERRVIMSAPLSKELREKYNVRAIPIRKDDEVTVVRGSNKGREGKITTVYRLKWCVHVERVAREKSNGQSVPIPIHPSKVVITKLKLDKDREQILERIGKGREAAKARA | ATGACCGTCATGAACAACGGTATGTTCGACTTAATTTGTTCAATAAACCCTCATGGCAACTTCCTGTTCCTCGAATATGCCTTTTGATATTCTACTTTCTCGGAAGACACATTCTCAAACCGATTACACTCAGGAATTGTATTGGGAGGATACTGACTCTCGACTTCTCAATGATAGGAATCGCCTCTTCCCGCCGGAAGTCGCGCAAGGCGCACTTCAGCGCTCCCTCCAGCGAGCGCCGTGTTATCATGAGCGCTCCTCTGAGCAAGGAACTCCGTGAGAAGTACAACGTACGCCTCCCGTTATGCCACCTCTCCTAAACCGATCACCTCCCGTTGTCAGATTCTACACTCTTTGCTCGGAGGGACTTTCACAGGAGGATAGCCAGGGATCACTGCAAAGAACTGTGAAGGATGTTCGTTTCTGACAGATACCCTATAGGTTCGCGCCATCCCCATCCGCAAGGACGACGAGGTCACCGTTGTGCGCGGCTCCAACAAGGGACGCGAGGGAAAGATCACCACCGTCTACCGTCTCAAGTGGTGCGTTCACGTCGAGCGTGTCGCCCGTGAGAAGTCCAACGGCCAGAGCGTCCCTATCCCCATCCACCCCTCGAAGGTCGTTATCACCAAGCTTAAGCTGGACAAGGACCGCGAACAGATTCTGGAGCGCATCGGAAAGGGCCGTGAGGCTGCTAAGGCTCGTGCTTAA |
| L27 | A0A0K8LPP4 |  | - | MKFMKVGRVAIITRGRYAGKKVVIVQPNDTGSKAHPFPYAIVAGIERYPLKVTRRMGKKMVEKRSRIKPFIKVVNYNHLMPTRYTLELEGLKGAVSQETFKEVSTREDAKKNVKKALEDRYTSGKNRWFFTPLRF | ATGAAGTGTTAGTTGAATGCCGTCTCTTCTCGAGTTATCAATTATTCGAATATGAGTTCATACTGACAGGATGGTGAAATAGTCATGAAAGTGGGCCGTGTGGCCATCATCACCCGTGGCCGTTACGCCGGTAAGAAGGTACGACAAGCCTTTTCTTTTCCTACATGTTGTAGCGGGGAATTTGGATGAAGGATCTGGTTCGGATTTTTCGATGGTCGATAGTGAAAGGAGAGGGAGACGTTCCGGACGAATTGGGGGCGAGAGATCGATTGGAACAGGATCAAGATTGGACATTGAGAAGGATGTGCTGACAAGGGACCTTTTCGCGAAATCACAGGTCGTCATTGTCCAGCCTAACGACACTGGCTCCAAGGCGCACCCCTTCCCCTACGCCATCGTCGCCGGTATCGAGCGCTACCCTCTCAAGGTCACCCGCCGCATGGGCAAGAAGATGGTCGAGAAGCGCAGCCGCATCAAGCCTTTCATCAAGGTCGTCAACTACAACCACTTGATGCCCACCCGTTACACTCTTGAGCTTGAGGGTCTCAAGGGTGCCGTCAGCCAGGAGACCTTCAAGGAAGTCTCCACGCGCGAGGACGCCAAGAAGAACGTCAAGAAGGCCCTCGAGGACAGATACACCAGCGGCAAGAACAGATGGTTCTTCACTCCTCTGCGTACGTGCAATCTTCGCTTATTGTCACTTGCAGTGAGATAGTTGTGTGCTGATTATGGTCTCTACACAGGTTTCTAA |
| L29 | - | BBXM01000162.1 (c25607-25042) | Met-loss | MAKSKNASQHHRSQKAHRNGIKKPKTHRYPSLKGVDPKFRRNHRHALHGTMKALKERKEGKREIA | ATGGCCAGTACGTTACCTGAACCCCGGCATTTTGGAATCCTCGAATCGATTCAATACCAGCACAATATCCCCGGTGTTTTCGGAAGTCGCTGACGGTCATTTTTCACTTTGAACAGAGTCCAAGAACGCGTCTCAGCACCACCGCAGCCAGAAGGCTCACCGTAACGGGTTCGTACAGTCCTCAACATTTTGAAATCGAAATATTTTGGTCGGATGGGAAGGATTGTTGCAGTGATAGATTTGACATCACGGAATCGGTCATGAATGACTCGCGACCGTCTTGCAACATATCCACCAACCACATTTTGAAGAAGGAGAAGACAGGTCGCTGATATTTGCCGTTTGATTCCAACAGTATCAAGAAGCCTAAGACTCACCGTTACCCTTCCCTCAAGGGTGTTGACCCCAAGTTCCGCCGCAACCACAGACACGCCCTTCACGGCACCATGAAGGCTCTGGTACGTTGACCTTTTGCTGCTGCTGAGTCGGAAGCAGCTGGGTGCCGGAGGACATGGCTAACTATCCTGCAGAAGGAGCGCAAGGAGGGCAAGCGGGAGATCGCATAA |
| L32 | A0A0K8LH39 |  | Met-loss | MVLAKKHVPIVKKRTKRFFRHQSDRFKCVPESWRKPKGIDNRVRRRFKSNIPMPSIGYGSNKKTKHMMPSGHKAFLVHNPKDVELLLMHNRTYAAEIASAVSSRKRVDIIAKAKALGVKVTNPRGRVTVEA | ATGGTCCTCGCAAAGAAGCACGTCCCCATCGTCAAGAAGCGTATGTTCCAGCTCCCTCTGCCAGCCTTTCCCTGCCTCAATCACACGCACAAAATTCGATTCTGGAAGAGATACAAGGGAAAAGATGATTTGGTTTGGATGTGATGGAAGAGACCGATCGAAGAGTTGGAATCAGCGATCGAATAAACACCTCGTTGCGAGCGTTGCATGATTACTTGAAAATTTACACACACACGAGAACATGGAATATCAGGAGGACTGACAACGGATGTGAACAGGCACCAAGCGCTTCTTCCGCCACCAGTCCGACCGCTTCAAGTGCGTGCCGGAGTCATGGCGCAAGCCCAAGGGTATCGACAACCGTGTCCGCAGACGCTTCAAGAGCAACATCCCCATGCCTTCCGTACGTTCAATTCCTCCGACCCTCAGCCGCAATCAGATTCGAATCATCTCGACGAGACAAATCTGGAATCGCTGTGGCCTACCTTTTGCGAACTCCAATGCTGATATTTCATGGATTAGATCGGTTACGGTAGCAACAAGAAGACCAAGCACATGATGCCCTCCGGCCACAAGGCTTTCCTCGTCCACAACCCCAAGGACGTTGAGCTGCTGCTCATGCACAACCGCACCTATGCCGCTGAGTACGTTACGTCTGTCTGAACAAAAAGTAACCCCATACTGATGAATTGGAATAGGATCGCCTCCGCTGTCTCTTCCCGCAAGCGTGTCGACATCATTGCCAAGGCCAAGGCGCTCGGCGTCAAGGTCACCAACCCCAGGGGCCGTGTCACCGTTGAGGCGTAA |
| L35 | A0A0K8LKJ2 |  | Met-loss, Acetylation | MSTSKVKAGQLWGKSKEDLSKQLEELKTELSQLRVQKITAGASSKTQRIHDVRKSIARVLTVINANQRAQLRLFYKNKKYTPLDLRPRLTRALRRRLTKHEATLKTEKQRKKEIHFPQRKYAVKA | ATGGTATGTTGAATGCGCAATTTTTGAATTCGGGCTTTTTTGGAAGTCGTTGGGGGTTCGTTATGCTGATGAATGTTAGTCGACATCCAAGGTCAAGGCTGGTCAGCTCTGGGGAAAGAGCAAGGAAGACCTTTCCAAGCAGCTCGAGGAGTTGAAGACCGAGCTCTCCCAGCTCCGTGTCCAGAAGATCACTGCCGGTGCCTCGTCGAAGACTCAGAGAATGTGCGTTTGAACTCGAAACCCTTCCTTTTGACATCTGCAATTCTACCTCGAAAAAATAAAAAAAAAAGTCCCCCAAATGTGGCATGGGAATTTATCGGAGGCACAACGGAAACGACAGACTGGGAAGAATCGGGGCCTACCAGAATCGCAGATTGCGCAATATACATGAGATGTTTTGGGGACAGGAATACTGATTTGCTGCGGTTCTACAGCCACGACGTTCGCAAGTCGATCGCTCGCGTTCTCACCGTCATCAACGCCAACCAGCGCGCCCAGCTTCGTCTGTTCTACAAGAACAAGAAGTACACTCCTCTTGACCTCAGACCCCGCCTCACCCGTGCCCTCCGCCGCAGACTCACCAAGCACGAGGCCACCCTCAAGACGGAGAAGCAGCGCAAGAAGGAGATCCACTTCCCCCAGCGGAAGTACGCCGTCAAGGTATGACCATCGCCATGTGCTCCCGCCTCATATCCATGCGGCCAGAAACCCTCGCAATCATGCGGTATATTCAGTTCAAGCCAGCGGAGTGACTAACGAGGAATTTCCCACAGGCCTAA |
| L36 | A0A0K8L642 |  | Met-loss | MAQERSGIVVGLNKGHKTTPLNTPKTRISRTKGQSSRRTAFVRDIAREVVGLAPYERRIIELLRNTQDKRARKLAKKRLGTFTRGKRKVEDMQRVIAESRRVAGH | ATGGCGCAGGAACGTTCCGGAATCGTGGTCGGTCTGAACAAGGGCCACGTACGTTGAATCCCTCTCCCGTTTCCCTAAGAAAATGAGTGTTTCCGGTCGGTATTTGTCCAGTCGACCTCGAAAGATTTGGATTGGGGATAGGAGCATAAAATATGGAGGGGACATATTGTCGGATTGTTAAGGCACAGTTCCTTCTTGAGATGGAGGAAATGGGACGACAACGGATGTACCAATCGAACTTACTGGGCCCGACTTGACAAAAATCGATCGGAAGCATTCGAGTTTGGTTTTAGAAATGAGGAACAATCAGTGAAGCTGACCTGGGTTCCTATCATAGAAAACCACCCCTCTCAACACCCCCAAGACCCGGATCAGCCGCACCAAGGGCCAGTCTTCCCGCCGCACTGCCTTCGTCCGTGACATCGCCCGCGAGGTTGTCGGTCTTGCCCCCTATGAGCGTCGTATCATCGAACTTCTGAGAAACACTCAGGACAAGAGAGCTCGTAAGCTCGCCAAGAAGAGGGTATGTCAAGAGTTCTGGATATTGGTCTTCATGGGTGTTCCTTTCGCTCGCTTCCCTGGGAATTGCATGATTGCAGTGTCTGCGGTTCGGAGTACAATCTGATGAAGCATACGGCAATTGTCGGTTGGAGACTACGCCCGCCACGAATCAGTCCGTTCAATTACGGAGATCACCGACCAGTGGCTTGTGGAGCGAGCTGGGGAACACTTGCTGAGAACCTTAGCAATCGTTCATGAACAATGCTAACGAATCATCTGTTATAGCTCGGTACCTTCACCCGTGGCAAGAGAAAGGTTGAGGACATGCAGAGAGTCATCGCCGAGTCCAGACGTGTGGCTGGTCACTAA |
| L38 | A0A0K8L6R6 |  | Met-loss | MPREVSDIKQFIEICRRKDASSARIKRNRSTQQIKFKVRCHRFIYTLVLKDSDKADKLKQSLPPALKVVDVSKGDKKKAL | ATGCCTCGCGAAGTTTCCGATATCAAGCAGTTCATCGAGATCTGCCGCCGCAAGGATGCCTCCTGTACGTTTTCCGCACACGATTTTCCCGGTTTTAGAGCAACATGAATAGGCTCAGATTCAGCTCCGTGGAAGGATTAGCGATAGAGTTGCATGAATGCTAACAATTGGTTTTCTCTCATTACAGCTGCCCGCATCAAGCGCAACCGCTCGACCCAGCAGATCAAGTTCAAGGTCAGATGCCACCGCTTCATCTACACCCTTGTCCTGAAGGACTCCGACAAGGCCGACAAGCTCAAGCAGAGCCTGCCCCCAGGTGCGTTGCCAACCTAAGTTTCTATTCGACTTTGCCCTTTCACGAATCACTATTCTTTGAAATAGTGTCTGGTGGTGGAAGTGAATAGTGGAATGAAAAGAGAAGCCGCGTTTCTGCTAGGATCTTAAGCTAACGCTACGTCTTCCAACAGCTCTCAAGGTTGTCGATGTGTCCAAGGGTGACAAGAAGAAGGCTCTGTAA |
| L39 | - | BBXM01000023.1 (c187659-187150) | Met-loss | MPSHKSFRTKQKLAKAQRQNRPIPQWIRLRTGNTIRYNAKRRHWRKTRLGI | ATGCCGGTTAGCAGTCCTCCCCTACGATTCCGAGTTATGTGAAGATTTTATTTGTCTTGCTCTCGCATCGAGGAACTGAGATATTCGTCTTTGATGTTGAGGTGACAAGGTGACAGTTGGCTAACGTCGATTTTTGTTGAATAGAGCCACAAGAGTTTCCGCACCAAGCAGAAGCTTGCCAAAGCTCAGAGACAGAACCGTCCTATTCCCCAGTGGATTCGTCTCAGGACCGGTAACACCATCAGGTAAATACACTCTTTTTCTTCTACAATCCGGAGCCGTCCATCGTGTGCAATGGTGGGGTGGGAAAATCCTTGAATCGGACCTCGATCACAACGACAGCCTTGTCATCCCGCATCAACAATCGCCCCAGAAAAATCACACGAACGGATTTGGAACCTTGCGCGGGTTACATGGACAGAAAAGGAAGAATGAACCAGCTGACGGGTATTCTTCTCCAGATACAACGCCAAGCGGAGACACTGGCGCAAGACCCGTCTCGGTATCTAA |
| L40 | A0A0K8LP90 |  | N-terminal scission (Release ubiquitin with 76 amino acids at the N-terminal side) | IIEPSLKALASKYNCEKSICRKCYARLPPRATNCRKKKCGHTNQLRPKKKLK | AGTACGTACAGCATCATGCTCTCCTACGTTATGTATTTGATCTATGGATTACGAGCTAACATGATCTTTTTTCTGCGATTTTACAGTGCAGATGTAGGTCTTCCTGAGCCGTTCGGACCAACCCAGTGCCCATTTGCCCAACCTCATCCCATTCATCGACTCGATCTACGCGGAACATCCGGAAAAGCCATGACTGACAAGTTTCGATCAATAGTTTCGTCAAGACCCTGACGGGTAAGACCATTACCCTCGACGTTGAGTCGAGCGACACCATCGACAATGTCAAGGCCAAGATCCAGGACAAGGAGGGTATCCCCCCTGACCAGCAGCGCCTGATCTTCGCTGGTAAGCAGCTTGAGGATGGCCGCACCCTGAGCGACTACAACATCCAGAAGGTACGCGAATTCTCTATCGGACGACGCTTGGGTTTCGTTTCATTATGCGCATGGGGTATTCTCGGATGATGCGGGTTGTTCGCTGGATTGCTGGACTTGGTTTCCGGATCCTCCGGCAGGGAACGTCTGCGCTAGGGGATGATTGGATTGTTACGGGGGATATATGACTATTGAGAAGGATATGTTGGGCTGACCGGACACTTGCACACATTTAGGAGTCCACTCTCCACCTCGTCCTCCGTCTGCGTGGTGGTATCATTGAGCCCTCCCTCAAGGCTCTTGCTTCCAAGTACAACTGCGAGAAGTCCATCTGCCGCAAGTGCTACGTACGTCTTTGTCCCGATCGCTCGACCCGACCTGTGAACCATTACAGGGGACGCTCGACTTGGAAGTCTTTTGCTAACCTGATCCCTGGATAGGCCCGTCTTCCTCCCCGTGCCACCAACTGCCGTAAGAAGAAGTGCGGTCACACCAACCAGCTGCGCCCCAAGAAGAAGCTCAAGTAA |
| L42 | A0A0K8LEN5 |  | Met-loss, Methylation | MVNVPKTRRTYCKSKECHKHTQHKVTQYKAGKASLFAQGKRRYDRKQSGYGGQTKPVFHKKAKTTKKVVLRLECTACKAKKQLALKRCKHFELGGDKKTKGAALVF | ATGGTAAGACTTATTTGCGTTTTCTTGCTCACCTGCTGTTTCCATCCTGGAGATTCGGAGGATTTCTGTCGAAAACTCGGTACTTGGTGATGCAACGAGATGTTCGTTCGGGGTTGAGGACGAAGAAGTTGCGGACAACGGAAAAATTGATGAAAATGCTATCGATCGCAAACCACGATAATTTTTGCTCGGATATGAGACATTGAACTATTCTCATTCGTCAGAGTTCAGGATAATATGGGATATTCCTCGAAGCGATCAGGACGCAACGAACGCCCGGACAACGAAAGACTCGGCGCACCAAACACGAATATGACGACGAATATTCTCTCACCAGAAGAACTTGTGATCTAGTTTCTGCGAGAAAGCTGAATGTCTTCCTACGATAACCGGTACCGAGTTTGATTGGATTCTCCGATGAACGGGACATGGAAACGCGCAGCACCAAGAAGTTGTTGGATTGTAGCGGACAGTTGCTGATAGCTCTTGCGTTCCAGGTCAACGTTCCCAAAACCCGCCGGACGTACTGCAAGTCCAAGGAGTGCCACAAGCACACCCAGCACAAGGTCACCCAGTACAAGGCTGGCAAGGTGAATACAACCCACACGCCATGGTAGACGAATTGGCACAGGCACTGATGATGGATCTATTAGGCCTCCCTGTTCGCCCAGGGTAAGCGTCGTTACGACCGGAAGCAGAGCGGTTACGGTGGTCAGACCAAGCCTGTCTTCCACAAGAAGGCCAAGACCACCAAGAAGGTCGTCCTGCGTCTTGAGTGCACTGCCTGCAAGGCCAAGAAGCAGCTCGCTCTGAAGCGTTGCAAGCACTTCGAGTTGGGGTACGTTGAGTCTGCGACTGTGGCGATATCTGATCCATATATACTAACGTGGGCTTTTCGCTGTGTAGTGGTGACAAGAAGACCAAGGGTGCTGCTCTTGTTTTCTAA |
| S24 | A0A0K8LDS5 |  | Met-loss, Acetylation | MADTPVTLRTRKFIRNPLLARKQMVVDVLHPNRANVSKDELREKLAELYKANKDQVSVFGFRTQYGGGKSTGFALVYDSHEALKKFEPHYRLVRIGAATKIEKASRQQRKQRKNRSKKFRGTAKTKGPKKNKD | ATGGCCGATACTCCCGTTACCCTGCGGACTCGCAAGTTCATCCGCAACCCCCTGCTCGCCAGAAAGCAGATGGTCGTGTAAGGAGCCCTTTTCTTCCTCCCTATCACGCGCTTGAGAATTTCCAGGGAGAGGGTCCCTTCTGGTCAATTTTTTTTTTGAAAAGTGATTTTGTGTGCTGGGAAGAAAAGATTTTGAGTGTGGAATATGCGGAACGACAAAGAGATACCAAGGCAACAAATGGCACTGATGATGGAGATTATCGGATTTTTATATCACCTTGGAATACTATCATGAAGGAAGGACCTGGAAATTCTCAAAGCACAGATGCAAAGGGCATTCTTTGTCGTGATGGTGATGATGAGTTTGACGATGATTCTAACATCGACTTCTTACAGGGACGTCCTCCACCCCAACCGGGCTAATGTCTCCAAGGACGAGCTCCGCGAGAAGCTCGCCGAGCTGTACAAGGCCAACAAGGACCAGGTCTCCGTCTTCGGTTTCCGCACTCAGTACGGTGGTGGCAAGAGCACTGGCTTCGCCCTCGTCTACGACTCCCACGAGGCCCTGAAGAAGTTCGAGCCTCACTACCGCCTGGTCCGCATCGGTGCCGCTACTAAGATCGAGAAGGCCAGCAGACAGCAGCGTACGTTTGAGCCACCGCCCACTCACACCCTCGAGATTGAACCAGACATGCATACATGCCTTTCATATCTCCGGGGGTTCCTCAATCTAGCATTGCTAATCTCTCTTCCCGATTTATAGGCAAGCAACGGAAGAACCGTTCCAAGAAGTTCCGCGGTACCGCCAAGACCAAGGGACCCAAGAAGAACAAGGACTAA |
| S27 | A0A0K8LBA7 |  | Met-loss, Methylation (2) | MVLAVDLLNPTPQAEARKHKLKTLVPAPRSFFMDVKCPGCFTITTVFSHAQTVVVCAGCSTVLCQPTGGKARLTEGCSFRRK | ATGGTAGGTTCTACTTGCATTTTAGCCTCATGCAACACCTTGGGATATCTCCTAGCCGTTCTGCTTGTCGTCTGCACAGGCTAAGGAGCTTGCTGCCCGTCGTGTGCCCGCCGTCGCCGGACCGTTTCATCACAGAAAGACATCAAAGTATCATCGACTAACACGATTTTTCTCTTTCACAATAGGTTCTCGCGGTCGACCTCCTCAACCCTACTCCTCAGGCTGAGGCTCGCAAGCACAAGCTTAAGGTATGATACCGAAATACATGTCCACCAAGGTCCTGGGTCCGATGGCTGATGTTGGGATCCGTTTCGACAAATAGACCCTTGTGCCTGCTCCCCGTTCCTTCTTCATGGACGTCAAGTGCCCCGGCTGCTTCACCATCACCACCGTCTTCTCCCACGCCCAGACTGTCGTCGTCTGCGCCGGCTGCTCGACCGTCCTTTGCCAGCCCACCGGTGGCAAGGCCAGACTCACTGAGGGCTGCTCCTTCCGGAGGAAGTAA |
| S29 | - | BBXM01000129.1 (c205483-204959) | Met-loss | MTHESVWYSRPRKFGKGSRECRVCAHRAGLIRKYGMDICRQCFREKAQDIGFYKYR | ATGACTCACGAGTCCGTTTGGTACAGCCGGCCCCGCAAGTAAGGATATTCTTCAATTCTGGGATGCGTCGATACGATTTGGCTAATTTATTACCCTTTTCCCTCGATAGGTTCGGCAAGGGTTCCCGTGAATGGTGTGTGAACGAAATTCCCCAGGCGATACCAGGAGGATAGGTCGAGATTTTATGAATTGCGAGCAGCAATGGCTAATCAGATATTTGCGTCGAAACAGCCGTGTTTGCGCCCACCGCGCTGGTCTCATCCGCAAGTACGGGGTATGGAAATGAGTCAACTATCGAGAATAACCCGCGTTGGATATGCGACTGATAGTGGTTATAGATGGACATCTGCAGACAGTGCTTCCGTGAGAAGGCTCAGGACATCGGTTTCTACAAGGTTGGTTCCCACATACGCTGCAGAGTCGGAACACCATGGCCAGGTTCAACCCGACGACATTCTCGGAGAACTGGTTGGCAGAAGAAGAGCGGATCACTAACAGATCACCTCTACCATCCAGTACCGTTAA |
| S30 | - | BBXM01000045.1 (c171685-171100) | Met-loss | MGKVHGSLARAGKVKSATPKVEKQEKKKEPKGRALKRLKYTRRFVNVTMTGGKRKMNPNPTS | ATGGGTAAGGTTCACGGCTCTCTCGCCCGTGCGGGTAAGGTCAAGTCTGCGACTCCTAAGGTGAGTTCTTCTTGTTCTTCCTTAAATGAAGTTTTGATCTCCGTGGTCCTGCTGGGAAGGGTTTATCGTCGCCACCGGAAGAGGAGGGATTTTGAGGAAGATTCTTGCAGCGGTTCGACATGCAGAAATGGCAGAGACGATATATGCGAAGACGATTTAATGAACCGATTGTTTTCCCGGGAGATATTCGCTGAATCAGAGTGCTGATGGGTTATGTTCTACTGTACAGGTCGAGAAGCAAGAGAAGAAGAAGGAGCCCAAGGGCCGTGCTCTGAAGCGCCTCAAGTACACCCGCCGTTTCGTCAACGTTACCATGACCGGTGGCAAGCGGAAGGTATGTTGATATCCAGCTCGATGGAAAGGATGCATATATGCTCTTGGCGGGGAAGTTGGGAGTTTCTAGAGAGAGAGAGAACCCCCATTCTCCGTCTGTCCGTCAGATTTTGCAGGAGGTCCTTGGCATCGAAAAGGCATCAGACACTGACAAGGACATTCGTTACAGATGAACCCCAACCCCACTTCCTAA |
| S31 | A0A0K8LB72 |  | N-terminal scission (Release ubiquitin with 76 amino acids at the N-terminal side) | GKKRKKKVYTTPKKIKHKRKKTKLAVLKYYKVDGDGKIERLRRECPSPECGAGIFMAAMHNRQYCGKCHLTYVFDESK | ATGCAGATCTTCGTCAAGACCCTTACGGGTAAGACTATCACCCTCGAGGTGGAGTCTTCGGACACCATTGACAATGTCAAGTCCAAGATCCAGGGTATGCATCCCGTCCGAGCAATCTTTGACCCGAGAAGACTCTTAATCTGACTTGGTAACAGACAAGGAGGGAATTCCCCCCGACCAGCAGCGTCTGATCTTCGCTGGCAAGCAGCTCGAGGACGGCCGTACCCTTTCTGACTACAACATCCAGAAGGAGTCGACCCTGCACCTCGTGCTCCGCCTGCGTGGTGGTGGCAAGAAGCGCAAGAAGAAGGTCTACACCACCCCCAAGAAGATCAAGCACAAGCGCAAGAAGACCAAACTCGCTGTCCTCAAGTACTACAAGGTTGACGGCGATGGCAAGATCGAGCGTCTCCGCCGCGAGTGCCCCTCCCCCGAGGTATGTCAAGTTCCTCTTCTGAAAAGCAAAAGCTCGCAGCTAACATATCTGTAGTGCGGTGCTGGTATCTTCATGGCTGCTATGCACAACCGTCAGTACTGCGGCAAGTGCCACCTCACCTACGTCTTCGACGAGTCCAAGTAA |
| *A. clavatus* NRRL 1 | | | | | |
| L27 | A1CR44 |  | - | MKFMKVGRVAIITRGRYAGKKVVIVQPNDTGSKAHPFPYAIVAGIERYPLKVTRRMGKKMVEKRSRIKPFIKVVNYNHLMPTRYTLELEGLKGAVSPETFKEVSTREDAKKNVKKALEDRYTSGKNRWFFTPLRF | ATGAAGTGTTAGTTGATCGCCGCTACGTCTCTAGTTTTTCCAGTTATTCGGTATCAGAATATGCTGACAGGATGGTGAAATAGTCATGAAAGTGGGCCGTGTGGCCATCATCACCCGTGGCCGCTACGCCGGTAAGAAGGTACGACAGAGACTTTTTCTACGTTGTTTTTTTCGCGATATGGGACGGCAAGACGATTGGGAACGAGACGATTCGGTGGAGGGTCAAGAGAGAGAAAAAGATGGGATGGAATCGTTCTGAATACATGGGACATTTGGGACGACTTGGGATTGGATTTTGAAGATTGGGAGCTATTTGAAGGGACGTGCTGACCAGGGCCGTTTTTGTGCGAATCTACAGGTCGTCATTGTCCAGCCTAACGACACTGGCTCCAAGGCGCACCCCTTCCCCTACGCCATCGTCGCCGGTATCGAGCGCTACCCCCTCAAGGTCACCCGCCGCATGGGTAAGAAGATGGTCGAGAAGCGCAGCCGCATCAAGCCCTTCATCAAGGTTGTCAACTACAACCACTTGATGCCCACCCGTTACACTCTCGAGCTTGAGGGTCTCAAGGGTGCCGTCAGCCCCGAGACCTTCAAGGAGGTCTCCACACGCGAGGACGCCAAGAAGAACGTCAAGAAGGCTCTCGAGGACAGATACACCAGCGGCAAGAACAGATGGTTCTTCACTCCTCTGCGTATGTTGCAACATCCCGGGTTCGATCAGCAGTACGAGGGTTTTCGCTGACAGACTCTATACAGGTTTCTAA |
| L34 | - | AAKD03000002.1 (c601659-600890) | Met-loss | MANNRLQYRRRNPYNTRSNKVRIIKTPGGELRYLHIKKKGTAPKCGDCGIKLPGIPALRPREYSQISRPKKTVSRAYGGSRCAGCVKDRIVRAFLIEEQKIVKKVLKESQEKAAGKR | ATGGCGAACAACAGATTGCAATACGTGAGTTACTATCATCATCTATATCAATGCGATGAAATACTGACCTTTGACTTTTCTTCGACAGCGGAGACGGAACCCGTACGTTTGAAAATCTCCTGCCCACATTTCTACCCAGAACTGCCCTTTTTTCGCTCGCTCTATCGACATTGCTCGCTCCCGACTCAGCGGCATTGTCCTCATGACGACGATAAATTTCAAGACTTGATCCGACATTGGAAACACGATATTGATGGTTTTTTGCTCTACAGGTACAACACGCGGTCCAACAAGGTCCGCATCATCAAGACCCCCGGCGGCGAGCTCCGGTACCTCCACATCAAGAAGAAGGGCACTGCTCCCAAGTGCGGTGACTGTGGCATCAAGCTCCCAGGTGTGAGTGCTCCCGAATCAAATTCTGGCTAGCGAGTGGTTATCTGTGCGGATCTCTTTGGGTGTCCCCGATTTGTTTCGACTGTGAGGAGAGACGAAGTGGAGGGACCGAAAAGCGCAGATTTACAACCTCCAGCCAGTCGCTTGGGGTGAAGAACCAAAAAAAGCTGATTTGGAATGCCTAGATCCCTGCCCTCCGCCCCCGCGAATACTCCCAGATCTCCCGCCCCAAGAAGACCGTCAGCCGTGCCTATGGTGGTTCCCGCTGCGCCGGCTGCGTCAAGGACCGCATTGTCCGTGCTTTCCTGATCGAGGAGCAGAAGATCGTCAAGAAGGTCCTCAAGGAGTCTCAGGAGAAGGCCGCTGGCAAGCGCTAA |
| L35 | A1CQ64 |  | Met-loss, Acetylation | MSTSKVKAGQLWGKSKEDLSKQLEELKTELSQLRVQKITGGASSKTLRIHDVRKSIARVLTVINANQRSQLRLFYKNKKYLPLDLRPRLTRALRRRLTKHEATIKTDKQRKKEIHFPQRKFAVKA | ATGGTATGTGCAATGCGCATTTTTGATGCTCAGATGGAATCTTTTTGATTTGAGCGTTTTGTGTCTCGGTATGCTGACGGATTTTTCTTTCTTAAAAGTCGACATCCAAGGTCAAGGCTGGTCAGCTCTGGGGAAAGAGCAAGGAAGACCTTTCCAAGCAGCTGGAGGAGTTGAAGACCGAGCTTAGCCAGCTCCGTGTCCAGAAGATCACCGGCGGTGCCTCGTCCAAGACCCTGAGAATGTGCGTTTGAATCTCGAAAATCCCTTGTGCCTGTGTGTTTTGTGGATCAATCTGGATACCTACCTCGAATTTGCCAATCTACTCGGACGAAAGAAGAGAATCAAGCGAAACCGGCATGCAACGGAGATTGGAACAAATGCGAAAAGGGAATACAGATTGCGCCAAAATACAAAAACGAACACGGACACAAGAAACCAAATTCAATCCCTTGACAGAATACTAACGTTTCGTCTTTTCCGCTCAACAGCCACGACGTTCGCAAGTCGATCGCTCGCGTTCTCACCGTCATCAACGCCAACCAGCGCTCCCAGCTTCGTCTGTTCTACAAGAACAAGAAGTACCTTCCTCTCGACCTCAGACCCCGCCTCACCCGTGCTCTCCGCCGCAGACTCACCAAGCACGAGGCCACCATCAAGACCGACAAGCAGAGAAAGAAGGAGATCCACTTCCCCCAGCGGAAGTTCGCCGTCAAGGTATGACCGCGTCCAAGGCACCCTTTTCACTTGACCTACACGGAATTATCCCAATTCCGGCTCAGCTCAGTCTGCGACACAAGAACAAAAAAAGATAATTTCACCCAACAATCACTAACGGAGCATTCTTTCGTGCAGGCTTAA |
| L36 | A1CH55 |  | Met-loss | MAQERSGIVVGLNKGHKTTALDTPKTRISRTKGQSSRRTAFVRDIAREVVGLAPYERRIIELLRNTQDKRARKLAKKRLGTFTRGKRKVEDMQRVIAESRRVAGH | ATGGCGCAAGAACGTTCCGGAATCGTGGTCGGTCTGAACAAGGGCCACGTACGTTGATTCTCCCTTCCCGTTTTTCTCCAGGACGGAAAGAAGAATGTTTCCGGTCGGTCTAAGTCGAGTCGAGCTTGATACAAATGTGTTGAAGCGAGGGATTGATAATTGGCGTGGAGGGGACATATTGCCGGACTTTGGAAAGGCAATATGAATAAATTTTCGACGTCAGGAAAGAGAAGGATCAAGACATGGAAAAGGGAACGCATCAATTGACCACATTGGGTTCGACTCGGCTACAGACCCCATTGGAAACATTCGACTTTCAATACAAATTCTCTTCGATGGCTTGAGTTGTTATGGAAACTGACATGTGGATTTTTGCATGTTACAGAAGACCACCGCTCTCGATACCCCCAAGACCCGGATCAGCCGCACCAAGGGCCAGTCTTCCCGCCGCACTGCCTTTGTCCGCGACATCGCCCGTGAGGTCGTCGGTCTTGCCCCCTATGAGCGCCGCATCATCGAACTCCTGAGAAACACTCAGGACAAGAGAGCCCGCAAGCTCGCCAAGAAGAGGGTACGTTTTGAACTTTGGGACGCTGGTCTTTTAGGTGTTGCCTCATATGCTCGCTTCGTTGGGAACTCGGATAATACGATCTCGATCCGGGTTACGCTTTCGTCGAACTCAAGGACCATCACCACTTGTTTATGTTTCTTCCTATCGACTGCTTAACGATCTCATTTTCGCCAATCCTTGCCGAGTCTCATCAACGGTTGACCAACGAAGCGAGCTTAGATAGCAACCCTGAAAGACTTTGTGTCTTGTTGCATCATACTCGATCATGCTAACAGATATCTTCCCACAGCTCGGTACCTTCACCCGCGGCAAGAGAAAGGTTGAGGACATGCAGAGAGTCATTGCCGAGTCCAGACGTGTGGCTGGTCACTAA |
| L39 | - | AAKD03000023.1 (c186396-185837) | Met-loss | MPSHKSFRTKQKLAKAQRQNRPIPQWIRLRTGNTIRYNAKRRHWRKTRLGI | ATGCCGGTTAGCAGTCCTCCCCTATGTTTTCGAAAATATCCTCGATGTCAGGATATCTTTTTTCAACGCTTTCGCCCTCGATGACGAAAGGAAATAAAGTGTTCCTTGATGGAGTGACAGCCCGCTAACGTGCCTGTTCGTTGTGATTAGAGCCACAAGAGTTTCCGCACCAAGCAGAAGCTTGCCAAAGCCCAGAGACAGAACCGTCCTATTCCCCAGTGGATTCGTCTCAGGACCGGTAACACCATCAGGTAAATACCCTCTTTTTCTCCTCTCTACATGGCATTTCGAAGAAAAAAAAATCCTTCGAACTCGGGATGGGAAAAAAAAAAGGGAATGGAACGGGTCGACGCCTCGCCTACACCACTCCTCTTGCGATACCTACCTCGTTATTATGCGTTCAACAATCAAACGAGGAAGGGAGCGAAGGATTTGGAAACTACTGTCTTGTTCAAGGACTGCGAAAGAACAACCCCCCAGCTGACGGTTTCTCTTTTCTCTTTTCTACCAGATACAACGCCAAGCGGAGACACTGGCGCAAGACCCGTCTCGGAATCTAA |
| L40 | A1CRQ4 |  | N-terminal scission (Release ubiquitin with 76 amino acids at the N-terminal side) | IIEPSLKALASKYNCEKSICRKCYARLPPRATNCRKKKCGHTNQLRPKKKLK | AGTTCATCAGCTAACATGCATTTTTTTCTGCGATTTTACAGTGCAGATGTAGGTCTTTTAAACCCTTGAACCGCACCATCCGGAGACTCCTTTGCCAGGCCCCCGAATCTACCGACGCGATACACGCGAGAGAATACGAACACCCATGCAGGAGAGAAAATGGCCATGACTGACAAGTTTCGGTTAATAGTTTCGTCAAGACCCTGACGGGTAAGACCATTACCCTCGACGTCGAGTCGAGCGACACCATCGACAACGTCAAGGCCAAGATCCAGGACAAGGAGGGCATCCCCCCTGACCAGCAGCGCCTGATCTTCGCCGGCAAGCAGCTTGAGGATGGCCGCACCCTGAGCGACTACAACATCCAGAAGGTACGCGAATTCCTTATCGGACGACATTTGGATTGAGATTCGGTGATGCGGAAAGTGAGAACTTGCCGCATGAAGCGTGCGGTTTCCTCGTTGCTGGAGTTGATGGGAATTATCCGGATCCTCCGGCACGAGAGCTATCGCGAAGAGGAGGATCGTCCATGAAAGGATTGGCATGGTTGTCGCATGGGATATGTTTTGCTGATGGGTCGCTTTTCACTCTTACAGGAGTCCACCCTCCACCTCGTCCTCCGTCTCCGTGGTGGTATCATCGAGCCCTCGCTCAAGGCTCTCGCCTCCAAGTACAACTGCGAGAAGTCCATCTGCCGCAAGTGCTACGTACGTCTAGCCTCTCCATCCGAATCCCGCATCGACATATTTGTGAAGACGGCATTTATGAACAGTGTTGGGTCACGGTCGCTGGATACGCTGCTAACTGGATGTCTTGAATAGGCCCGTCTTCCTCCTCGTGCCACCAACTGCCGTAAGAAGAAGTGCGGTCACACCAACCAGCTCCGCCCCAAGAAGAAGCTCAAGTAA |
| L42 | A1CSM9 |  | Met-loss, Methylation | MVNIPKTRRTYCKSKACHKHTQHKVTQYKAGKASLFAQGKRRYDRKQSGYGGQTKPVFHKKAKTTKKVVLRLECTACKAKKQLALKRCKHFELGGDKKTKGAALVF | ATGGTAAGGACTCATTTGCGTTTTCACTGCTCACATACTGCTTTCCTCCTGGAGATTCGGAGAATTTCTGTCGAAAACTCGATACTTGGCGATAAAAACGAGATATTGCGGTCGGGACTACGAACGGAGAACATGCGGGTAAAGGGGACAATTTTTTTTATGGGAAATGTCGTCGATCGCACATCGAGATAAAATATCCTCGAAGCAAGAAGGAACAAAAGAAAGAAATATTGTTGAGCAAAGAGATGGACTGATTCGACATGAGCCGAAAATAACACCAACCTTCAAGAAGCGAAAAGGCCGAATGCGCCAGAAGAGCTCGACGTTATCCACAAATCTCGTTTGGTCAGGAAGGACTTGATATGTCCCTCGACACGATCCAGTATCCAGTTCTGCGACAGAAATCTCTCCGATGACCAGGAAGGGGAAAATGCAGCATAGCTTTGTCGAGTCTTAGCAACAGTTGCTGATATATCTGCGCCCATCAGGTCAACATTCCCAAAACCCGCCGGACGTACTGCAAGTCCAAGGCGTGCCACAAGCACACCCAGCACAAGGTCACCCAGTACAAGGCTGGCAAGGTGAATACCACCCAGGAACACTGAGAGTTGGGTTGGCGCAGGCACTGATGTGGATATAATTCATAGGCCTCCCTGTTCGCCCAGGGTAAGCGTCGTTATGATCGCAAGCAGAGCGGTTATGGTGGTCAGACCAAGCCTGTCTTCCACAAGAAGGCTAAGACCACCAAGAAGGTCGTCCTGCGTCTGGAGTGCACTGCCTGCAAGGCCAAGAAGCAGCTCGCTCTGAAGCGCTGCAAGCACTTCGAGCTTGGGTACGTTCCTCCCGCGGCGTTGTCACTACCAGTGTATATCGGCCTACTAACATTCGACTTCTTCCGTATAGTGGTGACAAGAAGACCAAGGGTGCTGCTCTTGTTTTCTAA |
| S29 | A1CUB3 |  | Met-loss | MTHESVWYSRPRKFGKGSRECRVCAHRAGLIRKYGMNICRQCFREKAQDIGFYKFR | ATGACTCACGAATCCGTGTGGTACAGCCGTCCCCGCAAGTAAGGAATTTCTCGACTCCAGGACGTGGTGGTATAACATGGCTAATTGTGTGTTCTTCTCGACAGGTTCGGCAAGGGCTCCCGTGAATGGTGTGTGAACGAAATTCCCGTGACGATACATTGAGAAAAGCTCTCGAAATCTCTGGATTGCGATCAGAATTGGCTAATCAGATATCTGCGTCGAAACAGCCGTGTTTGCGCCCACCGCGCCGGTCTCATCCGCAAGTACGGGGTATGGAAAACAAATCGCAACGGGACAACGATATGACCAGGGAACATGGGCTGACGGGAGATACAGATGAACATCTGCAGACAGTGCTTCCGTGAGAAGGCTCAGGACATCGGTTTCTACAAGGTTGGTTCCCACATGCGCCGCGGATTCGGAAACACATTGCCAGCAACACAGATACGACAGATCAGAAGCATTGGCTGGCAAATGAGGAGCAGTTTACTAACAATCTATTTCCCAACCGACAGTTCCGTTAA |
| S31 | A1CKM6 |  | N-terminal scission (Release ubiquitin with 76 amino acids at the N-terminal side) | GKKRKKKVYTTPKKIKHKRKKTKLAVLKYYKVDGDGKIERLRRECPSPECGAGIFMAAMHNRQYCGKCHLTYVFDESK | ATGCAGATCTTCGTCAAGACCCTTACGGGTAAGACTATCACCCTCGAGGTGGAGTCTTCGGATACCATTGACAATGTCAAGTCCAAGATCCAGGGTACGTTCGCCCATCACTCGCTCAGAACTCTGCAGCCACGAAAAAAAAAAAAAAAAAAAGTGACCTCATTACTTACAAATCCGCACAGACAAGGAGGGAATTCCCCCAGACCAGCAGCGCCTGATCTTCGCCGGCAAGCAGCTCGAGGACGGCCGCACTCTCTCTGACTACAACATCCAGAAGGAGTCGACCCTGCACCTCGTGCTCCGCCTGCGTGGTGGTGGCAAGAAGCGCAAGAAGAAGGTCTACACCACCCCCAAGAAGATCAAGCACAAGCGCAAGAAGACCAAGCTCGCCGTCCTCAAGTACTACAAGGTTGACGGCGATGGCAAGATCGAGCGTCTCCGCCGCGAGTGCCCCTCCCCCGAGGTATGTGATTTTGTCTGTTTGCAGATGTGGTGACGACAGACACTGATTCGGAATGTTTAGTGCGGTGCTGGTATCTTCATGGCTGCTATGCACAACCGCCAGTACTGCGGCAAGTGCCACCTCACCTACGTCTTCGACGAGTCCAAGTAA |
| *A. niger* CBS 513.88 | | | | | |
| L39 | - | LC215002 | Met-loss | MPSHKSFRTKQKLAKAQRQNRPIPQWIRLRTGNTIRYNAKRRHWRKSRLGV | ATGCCGGTTAGCAGCCCTCCCCGTTATTCGAACGACCATGAATACCTCGTTTTTGCTGGAAATAAAAATAATGAATACGGCGACACGGAATAAGGACCAGTGGTCGGGATGATGAAGTGAACAGCAGGCTAACGCTCACCTTTTTTCAATATAGAGCCACAAGAGTTTCCGCACCAAGCAGAAGCTTGCCAAAGCTCAGAGACAGAACCGTCCTATCCCCCAGTGGATTCGTCTCAGGACCGGTAACACCATCAGGTAAATAAGAAACCGTCACCGTTCCATTTGCACGCACCCACACAACACCCCCGCGAAGTCCTCGATGAGTCCCAGCGAAGAACACAAAGAACATGAAAGGGAAGGGGATACCAGCGAAGGAAGAAACAAGAGAAGGATGCATAAGATGGTAGCACCAGATGACTCGCCATCACGCCAAATCATCAATCAACTAGGCAAACCGCTGGGAACAACATCGAAGGACACAGCACACTCCACACACTACTCTCATACACACAAACATTTTCACAGTCGAACACAATGAATACTAACATCTCCTTTTCTCAACCCAGATACAACGCCAAGCGTAGGCACTGGCGCAAGTCCCGTCTCGGAGTCTAA |
| L40 | A2Q831 |  | N-terminal scission (Release ubiquitin with 76 amino acids at the N-terminal side) | IIEPSLKALASKYNCEKSICRKCYARLPPRATNCRKKKCGHTNQLRPKKKLK | AGTACGTATACCCTCTTCATTCGTGAATGCGAAATGCGTGTTCCCTGAAGTCGCAAACTAACATGTCGTTTTTTCCGCGTCTCTGCAGTGCAGATGTAGGTCATTCCAAGTCCCCTGGCAACCACCCGTCGAATTACGGTCGAATCCCGAAAGATGGCACGAATATATCATCGAAAATGAAATAAGGAAACTGACCCAGGCTCTTCTCCGATTAATAGTTTCGTCAAGACCCTCACGGGTAAGACCATTACCCTCGACGTCGAGTCGAGCGACACCATCGACAACGTCAAGACCAAGATCCAGGACAAGGAGGGTATCCCCCCGGATCAGCAGCGTCTTATCTTCGCTGGTAAGCAGCTTGAGGATGGCCGCACCCTGAGCGACTACAACATCCAGAAGGTATGAGTGAAACGGTTCCGCGAATCATCTATACGTCGATTTCGTCTGCTCGGATAGTCGGTTACGAACATGATGCGATACGGGTCGGTTGGGGCGCCACAACATACTACTTGGGTTTGAGGGGAGGATCAATCGGCACGGTCGCATTCATGAACGTTTCACGACTATCGACATTCGATGCGACTTGACTATTGGGGATATAGTTTGACTGACGGGTTCTTTTTCTCGACTATAGGAGTCCACCCTCCACCTGGTCCTCCGTCTCCGTGGTGGTATCATCGAGCCCTCCCTTAAGGCTCTCGCCTCCAAGTACAACTGCGAGAAGTCCATCTGCCGCAAGTGCTACGTACGTCCATCCATCTATTCTACTTTTCTATTCTTCCGACTCGATCCCGCCCTCTTTTGAGTCCCGCCTAGCGAACCATTTCCCCCACTAACATTGATCCTCCGATTAGGCCCGTCTTCCCCCCCGTGCCACCAACTGCCGCAAGAAGAAGTGTGGTCACACCAACCAGCTCCGCCCCAAGAAGAAGCTCAAATAG |
| S21 | - | LC215003 | Acetylation | MENEKGEIVDLYVPRKCSATNRIIKANDHASVQISIAKVDENGRYTGENHTYALCGFIRARGESDDSLNRLAQRDGYVRNVWTAARQR | ATGGAGAACGAGAAGGGAGAGATCGTCGATCTGTATGTATCCATTCCTTCCAAAACCAAATAATGTTGAGTTTGTGTGTGGTGCGAGATGGAGATATGGATGCGATTCGGTGCTCGATATACGATGATATGATATGGCTTGGAGAGATATATCGCGGATATGTGCTACGGCGGGGAACATTATTGTCGAATCGACGAGGACATGGTTTTTAAATCAGGATCTACGATATGGAAGAGACGGAATTGAAGAAATATACGGAGAAAGGACAGGAGAAGGAAAGAGGAGGAATACCGAACACCGATCGCATCGCATATCTCTATCATCATCATCATCCAACGTCTCAATACATGCGCAATCGCCAAAAGGACAAGCCAAGGCAAAATGCTAATATTAAATTTAACAGCTACGTCCCCCGCAAGTGCAGCGCCACCAACCGCATCATCAAGGCCAACGACCACGCCTCCGTCCAGATCTCCATCGCCAAGGTTGACGAGAACGGCCGCTACACCGGTGAGAACCACACCTACGCTCTGTGCGGCTTCATCCGTGCCCGTGGTGAGAGCGATGACTCCCTGAACCGCCTTGCCCAGCGTGACGGCTACGTCCGCAACGTCTGGACCGCTGCCCGCCAGCGCTAA |
| S31 | A5AA93 |  | N-terminal scission (Release ubiquitin with 76 amino acids at the N-terminal side) | GKKRKKKVYTTPKKIKHKHKKTKLAVLKYYKVDGDGKIERLRRECPSPECGAGIFMAAMHNRQYCGKCHLTYVFDESK | ATGCAGATCTTCGTCAAGACCCTTACGGGTAAGACTATCACCCTCGAGGTGGAGTCCTCGGACACCATTGACAATGTCAAGTCCAAGATCCAGGGTAAGCATACACACACACACATACCTACAATCACCGGTATTTCTATCAGGCAACATTTACTAATGATTATTTACAGACAAGGAGGGTATCCCCCCGGACCAGCAGCGTCTGATCTTCGCTGGTAAGCAGCTCGAGGACGGCCGTACTCTTTCGGACTACAACATCCAGAAGGAGTCCACCCTCCACCTGGTGCTCCGCCTGCGTGGTGGTGGTAAGAAGCGCAAGAAGAAGGTCTACACCACCCCCAAGAAGATCAAGCACAAGCACAAGAAGACCAAGCTTGCCGTCCTCAAGTACTACAAGGTCGACGGTGATGGCAAGATTGAGCGTCTTCGCCGCGAGTGCCCCTCCCCCGAGGTATGTTTAATTCCGCCTTTGGTTGATCCCTGCAGTTGGCGCAGTTTACTGACTTCGTCTCTTAGTGCGGTGCTGGTATCTTCATGGCCGCTATGCACAACCGCCAGTACTGCGGAAAGTGCCACCTCACCTACGTCTTCGACGAGTCCAAATAA |
| *A. kawachii* IFO 4308 | | | | | |
| L27 | G7XSR1 |  | - | MKFMKVGRVAIITRGRYAGKKVVIVQPNDTGSKAHPFPYAIVAGIERYPLKVTRRMGKKTVEKRSRIKPFIKVVNYNHLMPTRYTLELEGLKGAVSTETFKEVSTREDAKKNVKKALEDRYTSGKNRWFFTPLRF | ATGAAGTGTTAGTGAAATGCTGCATCACCTCGACTCTTGATTTTTCTCGTCTTCGAATCCAAACTGACAGGATGTTACGATAGTCATGAAAGTGGGCCGTGTGGCCATCATCACCCGTGGCCGTTACGCCGGTAAGAAGGTACGACAACCGATTTCTCCATTTTCTGGCGAGGACATGCATATATATATACCCCGAGGTGGAATGGACTGGAAGGAATGATTTTTGCCGACTTGGAAGAAGAAATGATGAGTCTTCGATTTGCATGGGAATGAAATCATCGAGCGGAAATGCGATTCACGGGCGAACTACCATGGGGAATATAACGAACGGAATGTGCAAGGATAGATGTGCTGACGAGGGTTTCTTTCGCGAATGACAGGTCGTCATTGTCCAGCCTAACGACACTGGCTCCAAGGCGCACCCCTTCCCTTACGCCATCGTCGCCGGTATCGAGCGCTACCCCCTCAAGGTCACCCGTCGCATGGGTAAGAAGACCGTCGAGAAGCGCAGCCGCATCAAGCCTTTCATCAAGGTCGTCAACTACAACCACTTGATGCCCACTCGCTACACTCTCGAGCTCGAGGGTCTTAAGGGTGCCGTCTCCACCGAGACCTTCAAGGAGGTTTCCACCCGCGAGGACGCCAAGAAGAACGTCAAGAAGGCCCTTGAGGACCGCTACACCAGCGGCAAGAACCGTTGGTTCTTCACTCCTCTGCGTACGTCTTACTCTTATCACTTTTTGCAGCTACGTTGGCTGGGTCAGGTTCGCAATGCTGACGGCTCTTACAGGTTTCTAA |
| L29 | - | BACL01000046.1 (c35871-35136) | Met-loss | MAKSKNASQHHNSQKAHRNGIKKPKTHRYPSLKGVDPKFRRNHRHALHGTAKALKERKEGKREIA | ATGGCGAGTACGTGACACCCCTTTTGCTCCAAAATATTCAAACCCCCGTTATTTTTGCATTTCCCGTCCCTCTCGGACGAAGAATGCGATTATCGACCATCTTGACATCGAAGAATGATGTATCGCTGACATGAATCTCTACAGAGTCCAAGAACGCGTCTCAGCACCACAACAGCCAGAAGGCTCACCGTAACGGGTGCGTATAATATGCCGATTTTCCTCCTACTTTCTTATCTGTTCCATACACGAATCGCCCGAGGACGAATATCACGATGTTGGATAGCGGACGGTATCTTCGACAAATTGAGGTTCAGCGAACGGGATAAGGAGGAAGAGGATAGATACTCTCGCCCTGATGATGGAGGATATCGACCAATATCCAACATTCGACATATTCGTCCTCGGGAATCAACACACAACAACTACAACGAAACCGCTTGAGAGAGATCGAAACACTGACCATCGCCTTCTTTTTCCAACTCAACAGTATCAAGAAGCCCAAGACCCACCGTTACCCCTCCCTCAAGGGTGTCGACCCCAAGTTCCGCCGCAACCACAGACACGCCCTTCACGGAACCGCCAAGGCTCTGGTACGTTTCTCTCTAAGATCGATCCAACAAGAAGTGTCTCTTCTGTTGGGCGAAGCCGAAATGAAGAATCGACACTAACACCCCGAACAGAAGGAGCGCAAGGAGGGCAAGCGCGAGATCGCATAA |
| L32 | G7XX33 |  | Met-loss | MVLAKKHVPIVKKRTKRFWRHQSDRFKCVPESWRKPKGIDNRVRRRFRGNIPMPSIGYGSNKKTKHMMPSGHKAFLVHNPKDVELLLMHNRTYAAEIASAVSSRKRVDIIAKAKALGVKVTNPKGRVTTEA | ATGGTCCTCGCAAAGAAGCACGTCCCCATCGTCAAGAAGCGTATGTCCTTTCTTTTCTCCCTGCTAGTCCCAGCCCAGTCCAGTTCCCAGCCAGAGTACGATCCGTCGGTTGTCAGTCGTCCAGCAAGCATGAGGAATATATACGGTGCGATGGAATGAGAAATCAGACGATCGGAGCGAACGAATCAATTTTTCTATATTTCTTATTGCGAAAGAACGATGGCGAACGACGAACTTGATCGGCGAAGAAATTATCACAAGCGACCGAAATTTCGATTCGATCTGGACGAAGATATTTACACACCAACAACATAAATTGACAAATGCTTGCTGGACCACGGACATACAGCCGGATTGAACTAAATCACACATACACACGTCGTCAGGACGAACGACCCGACTAACATCAAACTCCTTCCAGGCACCAAGCGTTTCTGGCGCCACCAGTCCGACCGCTTCAAGTGCGTGCCGGAGTCATGGCGCAAGCCCAAGGGTATCGACAACCGCGTCCGTAGACGCTTCCGTGGCAACATCCCCATGCCCTCCATCGGCTACGGATCCAACAAGAAGACCAAGCACATGATGCCCTCCGGCCACAAGGCTTTCCTCGTCCACAACCCCAAGGACGTTGAGCTCCTGTTGATGCACAACCGCACCTACGCCGCTGAGTACGTCCATCCACTGCCTAAATCCACTTTCCACCACAAGTCCAAAATACTAATTACTTGAATAGGATCGCCAGCGCCGTCTCCTCCCGCAAGCGCGTCGACATCATCGCCAAGGCCAAGGCTCTCGGCGTCAAGGTCACCAACCCCAAGGGCCGCGTCACCACCGAGGCTTAA |
| L33 | - | BACL01000072.1 (118887-119762) | Met-loss | MPSEQGHRLYVKGRHVSYQRSKRTVNPNTSLIKIDGVESTEAANFYLGKKVAFVYRAKREVRGSNIRVIWGKVTRPHGNSGVVRAQFRHNLPPKTFGATVRVMLYPSNI | ATGCCTTCGGAACAAGGTCACAGACGTGAGTGCCATCATTTTCCGGATATCAGATATGTGTGCGCGATGGTTGGAATATGGGGATGGAGAAGGATAGATGGGGATATGCGGGCGCATATTGGGCGAAATGCTGAATGAAATGCTAACTGAATATTTTTGCGAATTATAGTCTATGTCAAGTAAGTTCACCACAAACCGTCCGCCGTCGAATTTGCGCACATTCCGGATATGAATATCAAAAATATCAAGATCCATGTCGGAACATCAGAGATACGGGCCAAGCGATGGAATGCAACGAGGACAGACCGGTTAATACGAATTCTTTCTACAGGGGTCGCCACGTGAGCTACCAGCGCTCCAAGCGTACCGTCAACCCCAACACCAGGTACTTGCGATCCGCACCGATATCGATATAGAGGAACAAGGGAATCTGACACATGGGTTATCTAGTCTGATCAAGATCGACGGTGTTGAGAGCACTGAGGCCGCAAAGTGAGTAGCGAATGGGTCCGGGATCGAAATATCAGAACACACAGCTAAGACGGGATTTTTTACAGCTTCTACCTGGGCAAGAAGGTTGCTTTCGTCTACCGGGCCAAGCGTGAGGTCCGGGGCTCCAACATCCGGGTCATCTGGGGCAAGGTTACCCGTCCCCACGGTATGTGATGCACAGCTCCATTGCGCGACGATTGCCACGGAATATATATCTATCTATATGTTATCGGTGATTGCTGGAACCGGGAACGTTGGAAACTAACAATACTTCCCTCATGAACAGGTAACTCCGGTGTTGTCCGTGCTCAGTTCCGCCACAACCTCCCCCCCAAGACCTTCGGTGCTACCGTCCGTGTTATGCTGTACCCCTCCAACATCTAA |
| L36 | G7X6Q0 |  | Met-loss | MAQERSGIVVGLNKGHKTTPLNTPKTRISRTKGQSSRRTAFVREIAREVVGLAPYERRIIELLRNTQDKRARKLAKKRLGTFGRGKRKVEDMQRVIAEARRTGAH | ATGGCGCAAGAACGTTCCGGAATCGTGGTCGGTCTGAACAAGGGCCACGTACGTTGTCCCCTGTCCTGAAGCTTGTTTTTTGAGTGTCCCGTTTTTGTCTTTGCGCGTGGAAAGATTTGGTCCGGTCGAGTCGACGAGATTTCAAGCGTGTGAGCGGTTTGCGGAAAGGTACAATTTGGATGGGAATGGAAAAGGAAAAGGCCGAGAAATTCGGGAGGATCTAAGAATTATATGTCCGGAGAAGGGTCTTTTTTTGTCTGCAATGCGGCGGAAGGATTCCTTTACAACGGCCCGACAAGTTCTTGACCTTTACATGCGACATGGGAAAGATTGGGAGCTGGGAATTGAAATCATCCGACTCGGCTGGATCATATCGGACTACTGCTGCTTTGATATCTCCAATTATGGGGAATTTCGCGGTTGAAATGCACAAGAAAGATCAAGGACAATCGGACTAACATGTCATTGATATCTTGCAGAAAACCACCCCCCTCAACACCCCCAAGACCCGGATCAGCCGTACCAAGGGCCAGTCTTCCCGCCGCACGGCCTTCGTTCGTGAGATCGCCCGCGAGGTTGTCGGTCTTGCCCCCTATGAGCGTCGTATCATCGAACTCCTGAGAAACACTCAGGACAAGCGCGCTCGTAAGCTCGCCAAGAAGAGAGTACGTTTGCCAATGCCTTGCCTTTGTTATCTGGAGGGATGGTATAGTTGGGAGGTTCGATTGTTTGCTTGTCCGCTTGCTCGCTCGATTGTCTCTTCGGATTTGGTCCGTTGTCTATGTGCATCGTGATTTTCGACTTCTCCACGGTTACGCTTGATCGGCATGTACGGACCCATCATTATTACCGGATATTACCTTGGAGAACAAACCGCCGTCAACAACCCATCCATCACCAACCGACCATGAATGCCAGCCTGAAACAACGAGCCAAACCGACGAAGCAAGCAACACGCCAACCACGGAACCATCGAGCAAAACGAGCAAGCAAGCAAGCAATCTGAACCACCAACCTATCCACTTCCTCCAACTCTTCTATACCAAAACCACCTGCTAACCTACAATTTCCTACAGCTCGGTACCTTCGGCCGTGGCAAGAGAAAGGTCGAGGACATGCAGCGTGTCATCGCCGAGGCCCGCCGCACTGGCGCTCACTAA |
| L37 | - | BACL01000120.1 (c154480-153794) | Met-loss | MTKGTSSFGKRHNKTHTLCRRCGRRSFHVQKSTCANCGYPAAKTRKFNWSEKAKRRKTTGSGRMRHLKEVHRRFHNGFQVGTPKGARGPENH | ATGAGTACGTGCCAACGCGATATACCACCGCCCAGTCCGATCTATCGACGAGAACAACATCCAGACTGACATACCGATTCTGTTTACAGCGAAGGGTACCTCCAGCTTCGGAAAGCGCCACAACAAGACTCACACTCTTTGCCGGCGTTGTGGTCAGTATTTACACTACTCGGAAGATTTATATATGTCCCTCCGGGCGGGAATTGTTATACGGGATATCTCTACACAACCAACGGAATGGATGAGAGAAGGAACGGAATAATAGATAACTGCCGAGAGGAACATATATATGAAGTTATGGGGAAAACGACCAGATGGGCTAATACTCTTCGGATTGAATTCAGGCCGCCGCTCCTTCCACGTCCAGAAGTCGACTTGTGCCAACTGCGGTTACCCTGCCGCTAAGACTCGCAAGTGTACGAATCCTTTTCCCCAAACCCCCACCATCGAAGAACAACCGCTGGTTACTTAAAGAAAACCCTGGATATGGATTTTATGAAAAAGTGCTTGGATGAACAAATTGGCTTACGAAGTTCGTTTTATACAGTCAACTGGAGCGAGAAGGCCAAGCGCAGAAAGACCACCGGCTCCGGCAGAATGCGTCACCTCAAGGAGGTCCACCGCCGCTTCCACAACGGCTTCCAGGTCGGCACCCCCAAGGGCGCTCGTGGTCCCGAGAACCACTAG |
| L39 | - | BACL01000247.1 (88045-88684) | Met-loss | MPSHKSFRTKQKLAKAQRQNRPIPQWIRLRTGNTIRYNAKRRHWRKSRLGV | ATGCCGGTTAGCAGCCCTCCCTGTCATTCGAACGACCGTGAACCACTCCATTTTGTGCTGGAAAATAAAACAAATATGAATACGGCTACACGGAATAAGGAAAAGTGGTCGGGATGATGAAGTTGAACAGCAGGCTAACGATCAACTCTCTTCTTTCAATATAGAGCCACAAGAGTTTCCGCACCAAGCAGAAGCTTGCCAAAGCTCAGAGACAGAACCGTCCTATCCCCCAGTGGATTCGTCTCAGGACCGGTAACACCATCAGGTAAATAAGAAACCGTCACCTGTTCCCAATTTGCACGCAACCCATACAACACACCCCGCGAAGTCCTCGATGAGTCCCAGCGAAGAAATACAGGGAACATGAGAAGGAAAATGGAGTGATATCAGCGAACGAATAAACAAAGAGAAGGAATATTCAAGACGCAGCACCAGATGATTCGCCATCACACCAAAACCCCCAATCAACTAGTCGACCGCTGGGAACAACATCAAAGGACACAGCACATCCCAAATTCTACATCCTCACACACAAACCTACCATGAACCAAAAAAAAACACCGAATACTAACATATCTCTTCCCAATCCAGATACAACGCCAAGCGTAGGCACTGGCGCAAGTCCCGTCTCGGAGTCTAA |
| L40 | - | BACL01000210.1 (67489-68444) | N-terminal scission (Release ubiquitin with 76 amino acids at the N-terminal side) | IIEPSLKALASKYNCEKSICRKCYARLPPRATNCRKKKCGHTNQLRPKKKLK | AGTACGTATACCCTTTTCGTTCGTGAATGCGAAATGCGTGTTCTCTGAAGTCGCAAGCTAACATGTCGTTTTTTCCGCGTCTCTGCAGTGCAGATGTAGGTCATTCCAAGATTCCCTGGCAACCACCCGTCGAATCCCAGCCCGAAATCCGAAAGATGGCACGAATATATCATCGAGAAATGAAACAAGGAAACTGACCCAGGCTTCTTCCCGATCAATAGTTTCGTCAAGACCCTCACGGGTAAGACCATTACCCTCGACGTCGAGTCGAGCGACACCATCGACAACGTCAAGACCAAGATCCAGGACAAGGAGGGTATCCCCCCGGATCAGCAGCGTCTTATCTTCGCTGGTAAGCAGCTTGAGGATGGCCGCACCCTGAGCGACTACAACATCCAGAAGGTATGAAATGAACCGGTTCCGCGAAATATCTTATACGTCGATTTCGTCTGCTCGGATAGTCGGTTACGAACATGATGCGATTCGGGTCGGTGGGGGGCGACACAACACGCAACTAGGTTTTGAGGAGGGGGAATTATTCCATCGGCACGGTCGCATTCATGGACGTTTCACGACTATCGGCATCCGACGCGACATCAATCCACCGGGATATAGTTTGACTGACGGGTTATCTTCTCGACTATAGGAGTCCACCCTTCACCTGGTCCTCCGTCTGCGTGGTGGTATCATCGAGCCCTCCCTTAAGGCTCTTGCCTCCAAGTACAACTGCGAGAAGTCCATCTGCCGCAAGTGCTACGTACGTCCATCCATTCATCCATACTACTTCCTTACCCCTCGTCTCGATCCCGCCCCTCTCTTGAGTCCCGGCTAGCGAAAAATCCCCCCACTAACATTGATAACCCGATTAGGCCCGCCTTCCCCCCCGTGCCACCAACTGCCGCAAGAAGAAGTGTGGTCACACCAACCAGCTCCGCCCCAAGAAGAAGCTCAAATAG |
| S29 | - | BACL01000189.1 (24917-25669) | Met-loss | MTHESVWYSRPRKFGKGSRSCRVCSHRAGLIRKYGMNICRQCFREKSSDIGFHKYR | ATGACTCACGAGTCCGTTTGGTACAGCCGGCCCCGCAAGTAAGTTGAAATCCGTGCCGATGAAATTTGATATCTATATCTGTGTGTGGTTGCTAATTGATCTGTGATGGATAGGTTCGGCAAGGGTTCCCGTTCTTGGTGCGTATTCTGCGATTTTCCCCCGAAGAGATGATGTGTGGGATTGGGAGGGAGTTTCGGAGAGGAGAGAGAAATGAACCGAATGGAGGACATGAAAACGAAAACGTGAAAGGAAGTTATGTGCTGATTTCGTGTGTTGGAATAATACAGCCGTGTCTGCTCCCACCGCGCCGGTCTGATCCGCAAGTACGGGGTATGGATATCCACCACTGAAATCTACTGCGATGCGAAAAATATCGGGGGTGCTGGAATAATGGCTAACTGGTCTTCTTGCGTACAGATGAACATCTGCCGTCAGTGCTTCCGTGAGAAGTCCTCTGACATCGGTTTCCACAAGGTTGGTTTTTCACCATCACCCACATGCATATTTCCAGCACCAGTACCACAAATCACACATTATATCCGAAATGGCAATTGGCATGGCGAGGAGCGATATCGAAAGAGAAGAAAGAGAAGGGGCTTTGCGACAAACGAAGAATTGAAACATCGGAATGGAATGAAACGAAATGCCACTACGACAACGACAGCCAAAAACTACAATCTACGCCACACACAAAACCAGACCAAGAAAAGAATCCTAACTAACATCCCATTTCACCTCAAACAGTACCGTTAA |
| S30 | - | BACL01000062.1  (c185506-185029) | Met-loss | MGKVHGSLARAGKVKAATPKVEKQEKPKSPKGRARKRLVYTRRFVNVTLTGGKRKMNANPTQ | ATGGGTAAGGTATGTTCAACTGAGATAATCCCCTCTGCTGAAAATGATGATTTTGATGAAATGATGGGAAAATAAACTAATATGCTTTTGATTCCAGGTTCACGGATCGGTAAGCCTCGCCTCCTCGTCCATCGACGATATCCCAACACCACAAGACATCATCCATCTAACATAACTTCGCACATCCAATAGCTCGCTCGTGCCGGTAAGGTCAAGGCCGCCACCCCCAAGGTCGAGAAGCAGGAGAAGCCCAAGAGCCCCAAGGGCCGCGCCCGCAAGAGACTCGTCTACACTCGCCGTTTCGTCAACGTTACCCTGACCGGTGGCAAGCGCAAGGTATGAATGAACCAAACCGACCTGACAGATTCAAAATTCGACAAAGCGCGAAGAAGAGAGTCTGGAATAGAGGTTGAGTACAGTACGGCTTACACATATCTTTTTCTACTATCTACAGATGAACGCCAACCCCACCCAGTAA |
| S31 | G7XPI1 |  | N-terminal scission  (Release ubiquitin with 76 amino acids at the N-terminal side) | GKKRKKKVYTTPKKIKHKHKKTKLAVLKYYKVDGDGKIERLRRECPSPECGAGIFMAAMHNRQYCGKCHLTYVFDESK | ATGCAGATCTTCGTCAAGACCCTCACGGGTAAGACTATCACCCTCGAGGTGGAGTCCTCGGACACCATTGACAATGTCAAGTCCAAGATCCAGGGTAAGCATACACACACGGCCTACATAATCCAGTTAGAACATATTCTAATGATGATGATGTTCAGACAAGGAGGGTATCCCCCCCGACCAGCAGCGTCTGATCTTCGCTGGTAAGCAGCTCGAGGACGGCCGTACTCTTTCGGACTACAACATCCAGAAGGAGTCCACCCTCCACCTGGTGCTCCGCCTGCGTGGTGGTGGTAAGAAGCGCAAGAAGAAGGTCTACACCACCCCCAAGAAGATCAAGCACAAGCACAAGAAGACCAAGCTTGCCGTCCTCAAGTACTACAAGGTCGACGGTGATGGCAAGATCGAGCGTCTCCGCCGCGAGTGCCCCTCCCCCGAGGTATGTCTAATATTCCATTTTATGGCTGAATCATGCTGTTGGTGCATCTTGCTAACTTTGGATCTCCTAGTGCGGTGCTGGTATCTTCATGGCCGCTATGCACAACCGCCAGTACTGCGGAAAGTGCCACCTCACCTACGTCTTCGACGAGTCCAAATAA |
| *A. flavus* NRRL 3357 | | | | | |
| L29 | B8MY86 |  | Met-loss | MAKSKNASQHHNSQKAHRNGIKKPKTHRYPSLKGVDPKFRRNHRHALHGTMKALKERKEGKREVA | ATGGCCAGTACGTGCTGATCCCCCGGTTCTCCTGGATCAATATGGAGATTTTGATCCTGGGATTATTTCCCAAAATGACGGGATCTTTGGACTTCGTCGTTCGGAATCGCTGACGTCTTCCCTTTTGACAGAGTCTAAGAACGCGTCTCAGCACCACAACAGCCAGAAGGCTCACCGTAACGGGTGCGTACCATTCGATTTTTATCCTCCATGATACACGACTTCTCTCGACCGGAGTGGGATTGCCGGGATATGTTGTATTGTTGGGTGTGGTGGGCGGTTTATTTCGACAGCGAACTCGGGATGATCGAGATCAGATCCAGCAATGCAACATGTTCAGCCAATACCCCTCCGATATGGACACTACGCAATGAAACGATCAGGAGCAGGCCGCTGACATACTTGACTCGCAACAGTATCAAGAAGCCTAAGACTCACCGTTACCCCTCCCTCAAGGGTGTTGACCCCAAGTTCCGCCGCAACCACCGTCACGCTCTTCACGGTACCATGAAGGCTCTGGTACGTTGACAATCTCCCCTCCCAGCAATATCGACCTTGGACGTTTCGCCATACGATGATGAAGAAGATTGGCTAACAACTGCTACAGAAGGAGCGCAAGGAGGGCAAGCGCGAGGTCGCATAA |
| L39 | - | AAIH02000170 (c59201-58729) | Met-loss | MPSHKSFRTKQKLAKAQRQNRPIPQWIRLRTGNTIRYNAKRRHWRKTRLGI | ATGCCGGTTAGCAACCCCTGAAGCAAACCTAGAGTCTGTACTCGGGTGATATTTTTCCACATCATTTGGTCAAAATGGATGGGGGGCTTATGTGATCGCTGCTAACGCTCAATTTCTTCGATTAGAGCCACAAGAGTTTCCGCACCAAGCAGAAGCTTGCCAAAGCTCAGAGACAGAACCGTCCTATCCCCCAGTGGATTCGTCTCAGGACCGGTAACACCATCAGGTAATTAACACCTATTCCTTTCAGGACCCTCGATCGGCTGGAACAGTGGCAACGAGCTTTGAAACGTGTATGGAGTTGACATGCTCCCACTCGGAATCCCGTCGCTTTCTTTGCTACTTGGAGCGGAGGAAATATCTACTTGGATTTTGAGCTGGACACGTCGGAAGGACATGAACGTGCTGACAACCGAAATCCTAGATACAACGCCAAGCGGAGGCACTGGCGCAAGACCCGTCTCGGTATTTAA |
| L40 | B8N3G7 |  | N-terminal scission (Release ubiquitin with 76 amino acids at the N-terminal side) | IIEPSLKALASKYNCEKNICRKCYARLPPRATNCRKKKCGHTNQLRPKKKLK | AGTGGTGTCGTTATTTTGATATGGGGTGATGAGCTAATTTGTTCTTTTTTTCGCGCGTCTACAGTGCAGATGTAGGTTTCCCTCGTACTCCGCACCCATATTGAACCTTTAAACCCGCCGATGCGACCCAGAACCGGCTGCGAAAGATATGAAATGAACGATGACGGGGGAAACGTTGCTGACAAGATTCCGATGATAGTTTCGTCAAGACCCTCACGGGTAAGACCATTACCCTCGACGTTGAGTCGAGCGACACCATCGACAACGTCAAGGCCAAGATCCAGGACAAGGAGGGTATCCCCCCCGACCAGCAGCGTCTGATCTTCGCCGGTAAGCAGCTTGAGGATGGCCGCACCCTGAGCGACTACAACATCCAGAAGGTATGAAGGATCCCAATCTCGAGGTGTTTGATTCGGATGGCCTTTCGTGATCGGATATGAGAAACGTGCGATGCGGTTTGTTCGCGATGGAGATGTAGCAGATCCGATCGCACGGGCAACATATTGGGGACGAATTTGAGGCTCAGAATCTGGATATATACATTGTTGAATGAAAGGAATGGTCGGCTGATGGGCATCAACCTTATAGGAGTCCACTCTCCACCTCGTCCTCCGTCTCCGTGGTGGTATCATCGAGCCTTCCCTTAAGGCCCTCGCCTCCAAGTACAACTGCGAGAAGAACATCTGCCGCAAGTGCTACGCCCGCCTTCCCCCTCGTGCCACCAACTGCCGTAAGAAGAAGTGCGGTCACACCAACCAGCTCCGCCCCAAGAAGAAGCTCAAATAA |
| S26 | B8NNG9 |  | Met-loss | MVKKRANNGRNKNGRGHTKPVRCSNCARCVPKDKAIKRFTIRNMVESAAIRDISDASVFTDYAVPKMYLKLQYCVSCAIHGKIVRVRSREGRRNRAPPPRIRYNKDGKKLNPPQAAKAM | ATGGTCAAGAAGAGGGCGAACAAGTAAGGACCGACCCTATCTCTTATACCCGTCCGATTCCATTCCATGCGTACCTTTTGATATGACGCCTACGGTTCCGGTATATTCTACATGGCCGTGGGAGCCCGGTTACCGACGAGTGTTTCTTTGTTAAAAGAACGGGAAACGGAAAGGGGCAATTTCTGGAATCAGCGAAACTGACAATTTTACAGCGGTCGTAACAAGAACGGCCGCGGCCACACCAAGCCCGTGCGCTGCTCCAACTGCGCTCGCTGCGTCCCCAAGGACAAGGCCATCAAGAGATTCACTATCCGCAACATGGTTGAGTCCGCTGCCATCCGTATGTTCGAACCTGATACCCGCAATCAAACCCCATATTCGCCGCGCACTGTTGTTCGAAAGAGCTGGAATCGAAATATGGGATCGGAGAAGGAAATGTGAAAGAATAGGACAACAGCACGCACGAATTGATGGGATACGATTTCAACCGCAACTGGGGCTGACTCGACAATTCTTACTGTATAGGTGATATCTCTGACGCCTCCGTCTTCACCGACTATGCCGTCCCCAAGATGTACCTCAAGCTGCAGTACTGCGTCTCCTGCGCTATCCACGGCAAGATTGTTCGGTACGCATACACCCCCGACATATGATATACTTTGGCGACTTTTGTGGACCACTAGGAAGATTGAACCAAGCTGACGGATATCCCTATTACAGTGTCCGTTCCCGGGAAGGTCGTCGCAACCGTGCCCCTCCTCCTCGCATCAGGTACAACAAGGACGGCAAGAAGCTGAACCCCCCTCAGGCCGCTAAGGCTATGTAA |
| S27 | - | AAIH02000002 (c314919-314418) | Met-loss, Methylation (2) | MVLAVDLLNPTPQAEARKHKLKQLVPAPRSFFMDVKCPGCFTITTVFSHAQTVVVCAGCSTVLCQPTGGKARLTEGCSFRRK | ATGGTAGGTTCTGCCCGTTTTGTCCACCCATTACCTCGCGGATATCCTTCGACATAAGCGAAGATAAGGAGTGCGACTCTTTCATCTCGTGCTCGCCGTCGCCGTCGCCTCCCGGAGCCCGCTTCCCATCGAAATCATCCAAGTCCACGCTGCTAACAAGAATTTTTTCTCTCCCGACAGGTTCTCGCGGTCGACCTTCTCAACCCTACGCCTCAGGCCGAGGCCCGCAAGCACAAGCTCAAGGTATGATAAGACACCAATTCTGGGAACAGACAGAGTTGGTGGGTTGGATGGCTGACTGGTACCCTCGACATTATAGCAACTTGTGCCTGCTCCCCGCTCCTTCTTCATGGACGTCAAGTGCCCCGGCTGCTTCACCATCACCACCGTTTTCTCCCACGCCCAGACCGTCGTCGTCTGCGCCGGTTGCTCGACCGTCCTCTGCCAGCCCACCGGTGGCAAGGCCCGTCTCACTGAGGGCTGCTCTTTCCGCAGAAAGTAG |
| S29 | B8N234 |  | Met-loss | MTHESVWYSRPRTYGKGSRGCRVCTHRAGLIRKYGMNICRQCFREKSQDIGFHKYR | ATGACTCACGAGTCCGTGTGGTACAGCCGCCCTCGCACGTAAGGACTCCCCGTTTTCTCCCCGTGAATTTGATGATAAAGACGTTTTGGCTAATGGTGGTTCTTTCTGCGTATAGTTACGGCAAGGGCTCCCGTGGATGGTATGTGAAGGCTTGAAACCGCTCCCTTCCCTCTGGACGACGATGGGAACCTCGGGTTCTGCAGTATTGGTTCGAAATGGTAATGGGATATATGGGACTGATTGTGGTTTTTGCGATTTAGCCGCGTTTGCACCCACCGCGCTGGTCTTATCCGCAAGTACGGTGTATGTGGAATATATTGAGCTCCGAAAGAAATATGCTGGGATTGTGTGAAGCTGATATGGGGTGATTTATAGATGAACATCTGCCGTCAGTGCTTCCGTGAGAAGTCTCAGGACATCGGTTTCCACAAGGTTCGTGACATGGCCTGCGCGATTGAAGATTACCACCCACAACCCAGCAACCACGACGAAAGAAAAGATATTTGGAAAAATAAACAACGGAACACGTTGGGCTGGGGAGTGCAGATGGAGAAAGAAAGAACCCTATCACTAACGCACATGTTTTACCAAACTACAGTACCGTTAA |
| S31 | B8N3Q5 |  | N-terminal scission (Release ubiquitin with 76 amino acids at the N-terminal side) | GKKRKKKVYTTPKKIKHKRKKTKLAVLKYYKVDGDGKIERLRRECPAPECGAGVFMAAMHNRQYCGKCHLTYVFDESK | ATGCAGATCTTCGTCAAGACCCTTACGGGTAAGACTATCACCCTCGAGGTGGAGTCTTCGGACACCATTGACAATGTCAAGTCCAAGATCCAGGGTAAGCATTCTTCGCTCGCTACTCCGCACACGAAATGTAAAGTCACTGTACTAACGTGAACATGTAGACAAGGAGGGAATCCCCCCTGACCAGCAGCGTCTGATCTTCGCTGGCAAGCAGCTCGAGGACGGCCGTACTCTTTCTGACTACAACATCCAGAAGGAGTCCACCCTTCACCTCGTGCTCCGCCTGCGTGGTGGTGGTAAGAAGCGTAAGAAGAAGGTCTACACCACCCCCAAGAAGATCAAGCACAAGCGCAAGAAGACCAAGCTTGCCGTCCTCAAGTACTACAAGGTCGACGGTGATGGCAAGATCGAGCGTCTCCGCCGCGAGTGCCCCGCCCCCGAGGTACGTTTTCTCTGGATACCCCTCTGTGACTTGTGTGGCGAAATGACTAATTACTCTACAGTGTGGTGCCGGTGTCTTCATGGCTGCTATGCACAACCGTCAGTACTGTGGCAAGTGCCACCTCACCTACGTCTTCGACGAGTCCAAATAA |
| *A. oryzae* RIB 40 | | | | | |
| L35 | XP_003190676.1 ^a)^ |  | Met-loss,  Acetylation | MSSKVKAGQLWGKNKDDLTKQLEELKQELSQLRVQKITGGASSKTQRIHDVRKSIARVHTVINANQRAQLRLFYKNKKYLPLDLRPKLTRDLRRRLTKHEASLKTERQKKREIHFPQRKFAVKA | ATGGTTTCGTCGATAGTCTTCCAAGGTCAAGGCTGGTCAGCTCTGGGGAAAGAACAAGGATGATCTCACCAAGCAGCTGGAGGAGCTGAAGCAGGAGCTCAGCCAGCTCCGCGTCCAGAAGATCACTGGCGGTGCCTCGTCGAAGACCCAGAGAATGTGCGTTTGAAAATTGCTTTTTCTTTTTCGAAATCCCTATTTGCACCTGGTTACGCCAATACCCAAAAGAATATGTGCGCTAGATAGAAGTGGAGGGAAAGGAGGGAAAGATTTTACATCTACTGCGAAAAGAGGAGGGACTGCAACGACAGCGCAAAATTCTTTTTGGACAACCACAACGGGAAAACGGAAGCAGACCAGAAAATGCTGATTGTGAATTTGTTACAGCCACGACGTCCGCAAGTCGATTGCTCGTGTCCACACCGTCATCAACGCCAACCAGCGCGCTCAGCTCCGCTTGTTCTACAAGAACAAGAAGTACCTCCCTCTCGACCTTCGCCCCAAGCTTACCCGTGATCTCCGTCGCCGCCTTACCAAGCACGAGGCCTCCCTCAAGACCGAGAGACAGAAGAAGCGCGAGATCCACTTCCCCCAGCGCAAGTTCGCTGTTAAGGTATGAGGCAGTCATCTTCCCGACCACGCTCGCATCTGGGAAGACCCTTCTCTACGGAAAAGTGAACCAGTACTGATCGGTTGTTCGAACAGGCTTAA |
| L40 | XP_001820063.2 ^a)^ |  | N-terminal scission (Release ubiquitin with 76 amino acids at the N-terminal side) | IIEPSLKALASKYNCEKNICRKCYARLPPRATNCRKKKCGHTNQLRPKKKLK | AGTGGTGTCGTTATTTTGATATGGGGTGATGAGCTAATTTGTTCTTTTTTTCGCGCGTCTACAGTGCAGATGTAGGTTTCCCTCGTACTCCGCACCCATATTGAACCTTTAAACCCGCCGATGCGACCCAGAACCGGCTGCGAAAGATATGAAATGAACGATGACGGGGGAAACGTTGCTGACAAGATTCCGATGATAGTTTCGTCAAGACCCTCACGGGTAAGACCATTACCCTCGACGTTGAGTCGAGCGACACCATCGACAACGTCAAGGCCAAGATCCAGGACAAGGAGGGTATCCCCCCCGACCAGCAGCGTCTGATCTTCGCCGGTAAGCAGCTTGAGGATGGCCGCACCCTGAGCGACTACAACATCCAGAAGGTATGAAGGATCCCAATCTCGAGGTGTTTGATTCGGATGGCCTTTCGTGATCGGATATGAGAAACGTGCGATGCGGTTTGTTCGCGATGGAGATGTAGCAGATCCGATCGCACGGGCAACATATTGGGGACGAATTTGAGGCTCAGAATCTGGATATATACATTGTTGAATGAAAGGAATGGTCGGCTGATGGGCATCAACCTTATAGGAGTCCACTCTCCACCTCGTCCTCCGTCTCCGTGGTGGTATCATCGAGCCTTCCCTTAAGGCCCTCGCCTCCAAGTACAACTGCGAGAAGAACATCTGCCGCAAGTGCTACGCCCGCCTTCCCCCTCGTGCCACCAACTGCCGTAAGAAGAAGTGCGGTCACACCAACCAGCTCCGCCCCAAGAAGAAGCTCAAATAA |
| S31 | Q2UKT8 |  | N-terminal scission (Release ubiquitin with 76 amino acids at the N-terminal side) | GKKRKKKVYTTPKKIKHKRKKTKLAVLKYYKVDGDGKIERLRRECPAPECGAGVFMAAMHNRQYCGKCHLTYVFDESK | ATGCAGATCTTCGTCAAGACCCTTACGGGTAAGACTATCACCCTCGAGGTGGAGTCTTCGGACACCATTGACAATGTCAAGTCCAAGATCCAGGGTAAGCATTCTTCGCTCGCTACTCCGCACACGAAATGTAAAGTCACTGTACTAACGTGAACATGTAGACAAGGAGGGAATCCCCCCTGACCAGCAGCGTCTGATCTTCGCTGGCAAGCAGCTCGAGGACGGCCGTACTCTTTCTGACTACAACATCCAGAAGGAGTCCACCCTTCACCTCGTGCTCCGCCTGCGTGGTGGTGGTAAGAAGCGTAAGAAGAAGGTCTACACCACCCCCAAGAAGATCAAGCACAAGCGCAAGAAGACCAAGCTTGCCGTCCTCAAGTACTACAAGGTCGACGGTGATGGCAAGATCGAGCGTCTCCGCCGCGAGTGCCCCGCCCCCGAGGTACGTTTTCTCTGGATACCCCTCTGTGACTTGTGTGGCGAAATGACTAATGACTCTACAGTGTGGTGCCGGTGTCTTCATGGCTGCTATGCACAACCGTCAGTACTGTGGCAAGTGCCACCTCACCTACGTCTTCGACGAGTCCAAATAA |
| *A. nidulans* FGSC A4 | | | | | |
| L36 | Q5B4S8 |  | Met-loss | MAQERSGIAVGLNKGHKTTPLNTPKTRISRSKGKASRRTAFVRDIAREVVGLAPYERRVIELLRNAQDKRARKLAKKRLGTFTRGKRKVEDMQRVIAEARRVGAH | ATGGCTCAAGAACGTTCCGGTATTGCGGTTGGTCTCAACAAGGGCCACGTATGTATACTGATTTCCTTTATCTGATATCCTCCAAATGGTGAATGGATTTTCGATGGATGAATGGACGGATGGGATGGAGCTTCCGATGTCGAGTCGAATCGAATTGAGCGAGACGAGCAGAAAGCGATCGGTTTTACGTACGGTTCGGTTCGGATTATGTGGAAGTACACTCGGATTGAGTTGAAATGAAATTAGAGGTTGCGGGCAACGAAGAACACGAGACGAAATTTTACGCCCGTGTACGGCCGTCAAGAAGGGACTAGCAGGGAGATTATAGCGTCAAGAATGTTATCGGAAGTCACCGTATTTGCGTCAGCCTATGCCTACGTTGTCTATTCAGTTCGGCTATCTGCTCGGCTTATTTGATTCGACACGGAAACTCCGGACCATACAACTATCATCCATCCAGCAATCCATCCATCCTATTCTTCCAATCTGCGGTATTCCGAGGCCCCTGAACTAACGGGTCGTATCTGCTTTAGAAAACCACCCCCCTCAACACCCCCAAGACCCGCATCAGCCGCTCCAAGGGCAAGGCCTCCCGCCGCACCGCTTTCGTTCGCGACATCGCCCGTGAGGTCGTCGGTCTCGCTCCTTATGAGCGCCGTGTCATTGAACTTCTGAGGAACGCTCAGGACAAGCGCGCCAGGAAGCTCGCTAAGAAGAGGGTATGTCTGCGCCTTTTGCTGGTTATCCATGGCAACGAATGGGTTTTGCTCAGCAGAGCTTGATCACTCAACTTGGTTTATTTTGATGAATCAACTGAGTTCCCAATCTCGCTCGTTCAATGAATCCAACGCGCCCATAACTCTATCTTATTAAGAGAGCAAGCTAACCAATATCTTCCCAACAGCTCGGTACCTTCACCCGCGGCAAGAGAAAGGTCGAGGACATGCAGCGCGTCATCGCCGAGGCCCGCCGTGTCGGTGCTCACTAA |
| L40 | - | AACD01000065.1 (166585-167455) | N-terminal scission (Release ubiquitin with 76 amino acids at the N-terminal side) | IIEPSLKALASKYNCEKNICRKCYARLPPRATNCRKRKCGHSNQLRPKKKLK | AGTGATGCTACTCTCTTGGAACTGCCAGCTAACATATCTTTTTTCCGCGCCTTTCTAGTGCAGATGTAGGTCTTCCGAACATGCCATTAACCCCGTTGCCATCCACAACTCTCGTCTATCGTCAACCGAATTTACGCAATAAAAGAATGCCGGAAAATCGAGGGTGAAAGAAGAGGGCTATGAATATGTGCTGACTGTTCTTCTCGAATAGTTTCGTCAAGACCCTCACGGGTAAGACCATTACCCTTGACGTCGAGTCCAGCGACACCATCGACAACGTCAAGACCAAGATCCAGGACAAGGAGGGTATCCCCCCGGATCAGCAGCGTCTCATCTTCGCTGGAAAGCAGCTTGAGGATGGCCGTACCCTGAGCGATTACAACATCCAGAAGGTATGAGGCCTGGTCTACCGATTGAGTTTTGCGCGTGGTATAGATTTGCGGTTGCGGAGGCGGAAGGACGGTTATCTTGTGTCATAGTTTTGACGCCAGTTAGCCGCTCCTAGTTAGAAGGAGAAGGGGTGTGTCGCAAGGAGAATTTGCGGAAGAATTTTGATTGGGATGATGACGGTTGGCTGACTATGCCTCTCTCAATTTTATAGGAGTCCACTCTCCACCTCGTCCTCCGTCTTCGTGGTGGTATCATCGAGCCGTCGCTCAAGGCCCTCGCTTCCAAGTACAACTGCGAGAAGAACATCTGCCGCAAGTGCTACGTACGTCTACCGGATTACCACTCGACTCAGTTCCATCTTCGACGAAGGATTTGAGAACTAATACTAGAATAGGCTCGTCTCCCTCCCCGTGCTACCAACTGCCGTAAGAGGAAGTGCGGTCACTCCAACCAGTTGCGCCCCAAGAAGAAGCTCAAATAA |
| L43 | - | AACD01000127.1 (25054-25641) | Met-loss | MTKRTKKVGITGKYGTRYGASLRKQVKKMEVSQHARYVCTFCGKNTVKRQAVGIWECKGCKKTIAGGAYTVSTPAAAATRSTIRRLREIAEV | ATGACCAAGCGCACTAAGAGTGAGTATAGCCACGGAATTTTTGTTGACCTTTTGAACAGAAAAATTGAAAACAGAAGCTGACTTGCTTCGCAGAGGTCGGTATCACCGGTAAATATGGTACCAGGTATGCTTTAGCCAGAAACCCTCCCACGACTCCGGATTGAATCGAGCACGACTTTTTCGTTCACATCACGGCGAAATTTGGCGCTTCGATAATCAAACAACAAGCGATTGGACATTTGATTGACTATGGTTCGACAATAGATACGGTGCCTCCCTGCGTAAGCAGGTGAAGAAGATGGAAGTGTCCCAGCACGCCCGCTACGTCTGCACCTTCTGCGGAAAGAACACCGTCAAGCGCCAGGCTGTTGGCATCTGGGAGTGCAAGGGCTGCAAGAAGACCATTGCCGGTGGTGCCTACACCGTCTCGTAAGTCGACACTTTCACGCCCTCAGTCCGACAACTCATCTCGACGACAACGGCGTTTCGCTCGATGGATATACTAACATGGGACTTCAACTCAGTACCCCCGCCGCCGCTGCTACCCGCTCCACCATCCGTCGTCTCAGGGAAATCGCGGAGGTCTAA |
| S31 | G5EB17 |  | N-terminal scission (Release ubiquitin with 76 amino acids at the N-terminal side) | GKKRKKKVYTTPKKIKHKRKKTKLAVLKYYKVDGDGKIERLRRECPSPECGAGIFMAAMQNRQYCGKCHLTYVFDESK | ATGCAGATCTTCGTCAAAACCCTCACGGGTAAGACTATCACCCTTGAGGTGGAGTCTTCCGACACCATCGACAATGTCAAGACCAAGATCCAGGGTGTGCCTCCCTCCTAATCACCACGATTATCCACAAAACTGACTCTAAGCTCTACAGACAAGGAGGGTATCCCCCCTGACCAGCAGCGTCTGATCTTCGCGGGCAAGCAGCTCGAGGACGGTCGCACTCTTTCGGACTACAACATCCAGAAGGAGAGCACCCTTCACCTTGTCCTGCGTCTGCGTGGTGGTGGTAAGAAGCGCAAGAAGAAGGTCTACACCACCCCCAAGAAGATCAAGCACAAGCGCAAGAAGACCAAGCTCGCTGTGCTCAAGTACTACAAGGTTGATGGCGATGGCAAGATCGAGCGTCTCCGCCGTGAATGCCCCTCTCCCGAGGTATGCAATCTGTCTACAGTACATCCGAACTATAGCTAACTAGTTGCCAGTGTGGTGCTGGTATCTTCATGGCCGCCATGCAGAACCGTCAGTACTGCGGAAAGTGCCACCTTACCTACGTCTTCGACGAGTCCAAATAA |

a) NCBI reference sequence accession numbers.
