# Supplementary material for: Ribosomal subunit protein typing using matrix-assisted laser desorption ionization time-of-flight mass spectrometry (MALDI-TOF MS) for the identification and discrimination of Aspergillus species
Source: BMC Microbiol. 2017 Apr 26;17:100. doi: 10.1186/s12866-017-1009-3 (PMC5405522; doi:10.1186/s12866-017-1009-3)
Supplement: Supplementary file 3 — Mass spectra of genome sequenced sample strains used in this study. Figure SI-1. Mass spectra of RSPs of N. fischeri NRRL 181T. Figure SI-2. Mass spectra of RSPs of A. lentulus IFM 54703T. Figure SI-3. Mass spectra of RSPs of A. viridinutans IFM 47045T. Figure SI-4. Mass spectra of RSPs of A. udagawae IFM 46973T. Figure SI-5. Mass spectra of RSPs of A. clavatus NRRL 1NT. Figure SI-6. Mass spectra of RSPs of A. niger CBS 513.88. Figure SI-7. Mass spectra of RSPs of A. kawachii IFO 4308. Figure SI-8. Mass spectra of RSPs of A. flavus NRRL 3357. Figure SI-9. Mass spectra of RSPs of A. oryzae RIB 40. Figure SI-10. Mass spectra of RSPs of A. nidulans FGSC A4. (PPTX 472 kb) [file 12866_2017_1009_MOESM3_ESM.pptx]

## Slide 1
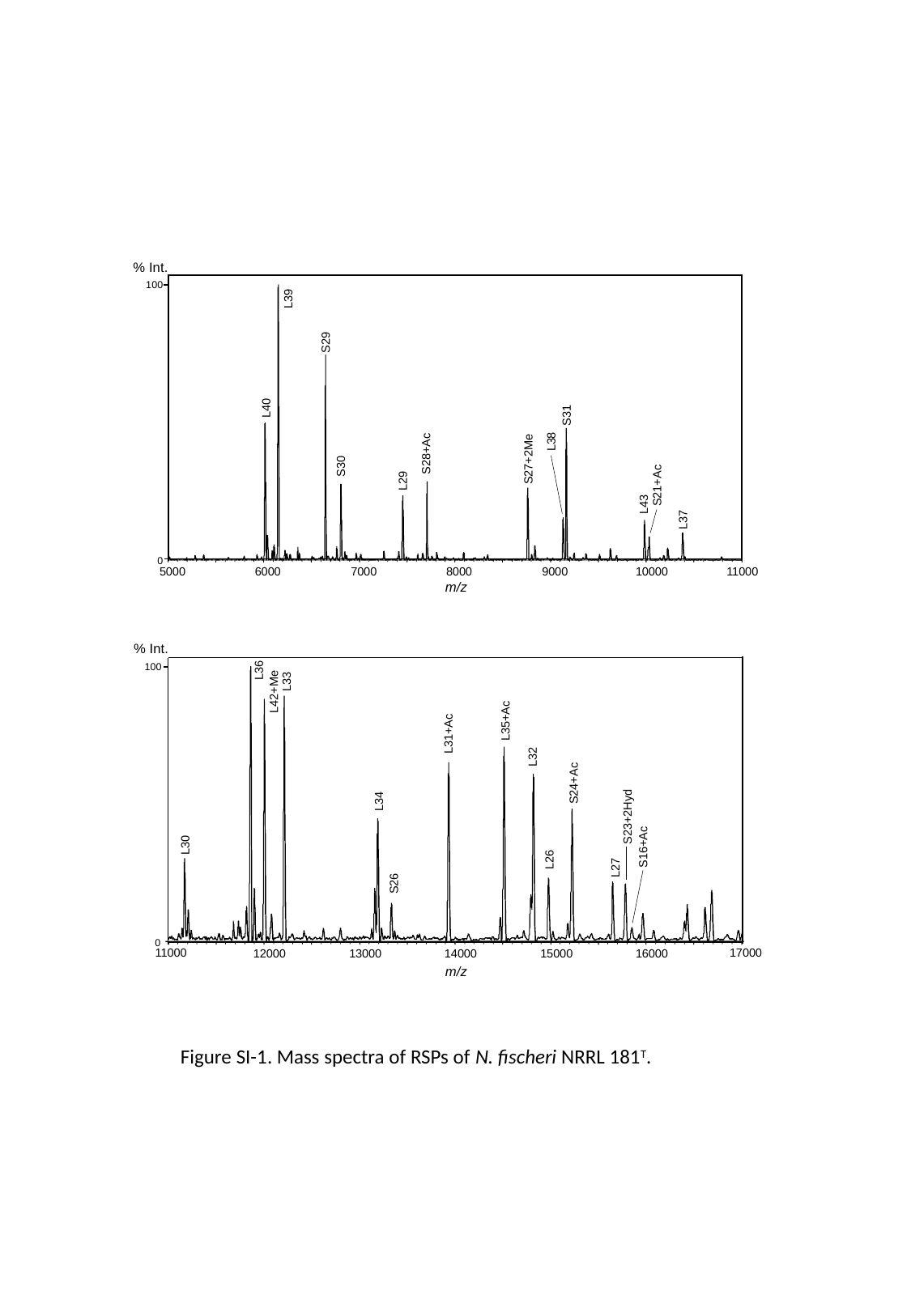

% Int.
100
L39
S29
L40
S31
L38
S28+Ac
S27+2Me
S30
L29
S21+Ac
L43
L37
0
5000
6000
7000
8000
9000
10000
11000
m/z
% Int.
100
L36
L33
L42+Me
L35+Ac
L31+Ac
L32
S24+Ac
L34
S23+2Hyd
L30
S16+Ac
L26
L27
S26
0
17000
11000
12000
13000
14000
15000
16000
m/z
Figure SI-1. Mass spectra of RSPs of N. fischeri NRRL 181T.

## Slide 2
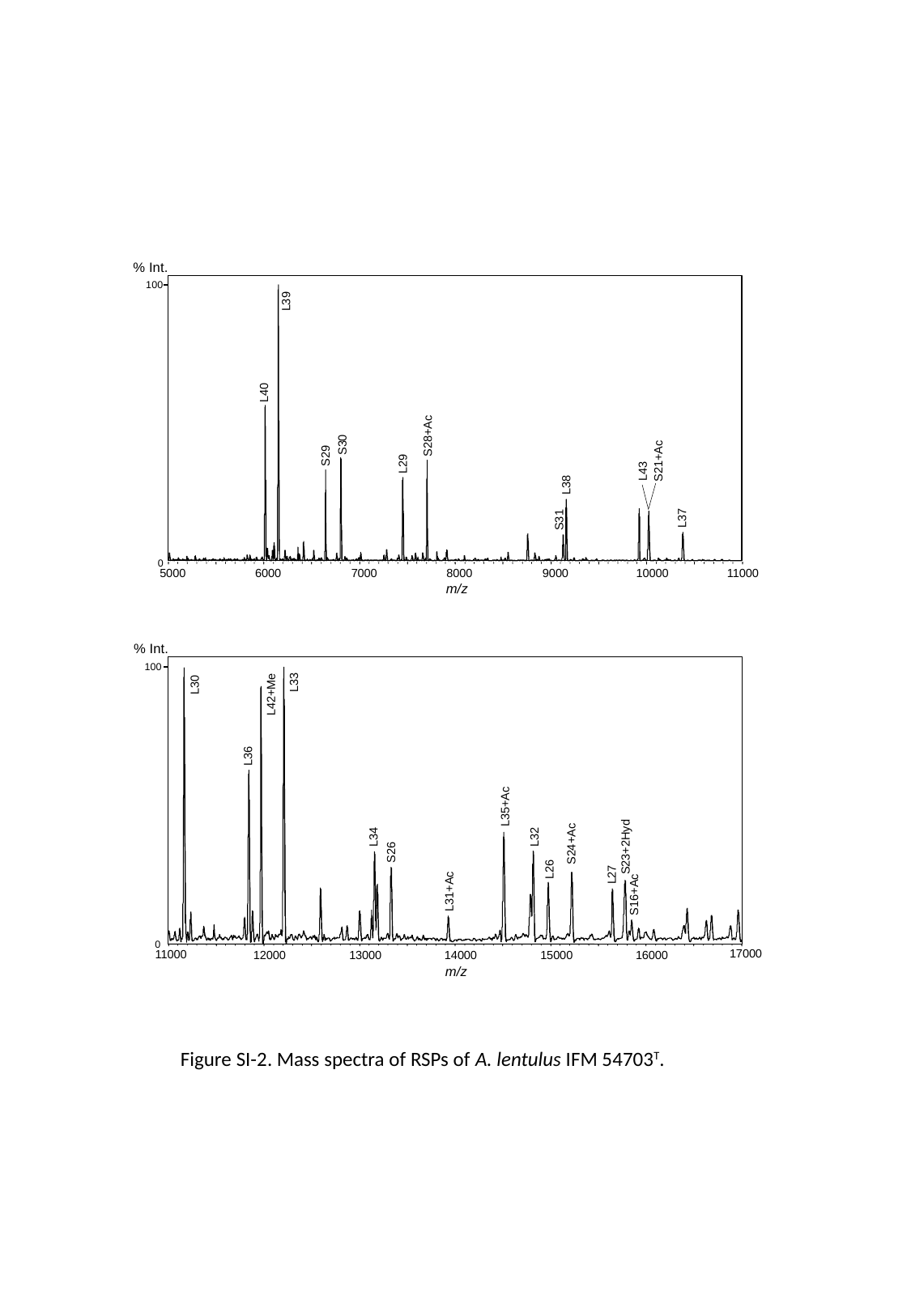

% Int.
100
L39
L40
S28+Ac
S30
S29
S21+Ac
L29
L43
L38
L37
S31
0
5000
6000
7000
8000
9000
10000
11000
m/z
% Int.
100
L33
L30
L42+Me
L36
L35+Ac
L34
L32
S24+Ac
S23+2Hyd
S26
L26
L27
L31+Ac
S16+Ac
0
17000
11000
12000
13000
14000
15000
16000
m/z
Figure SI-2. Mass spectra of RSPs of A. lentulus IFM 54703T.

## Slide 3
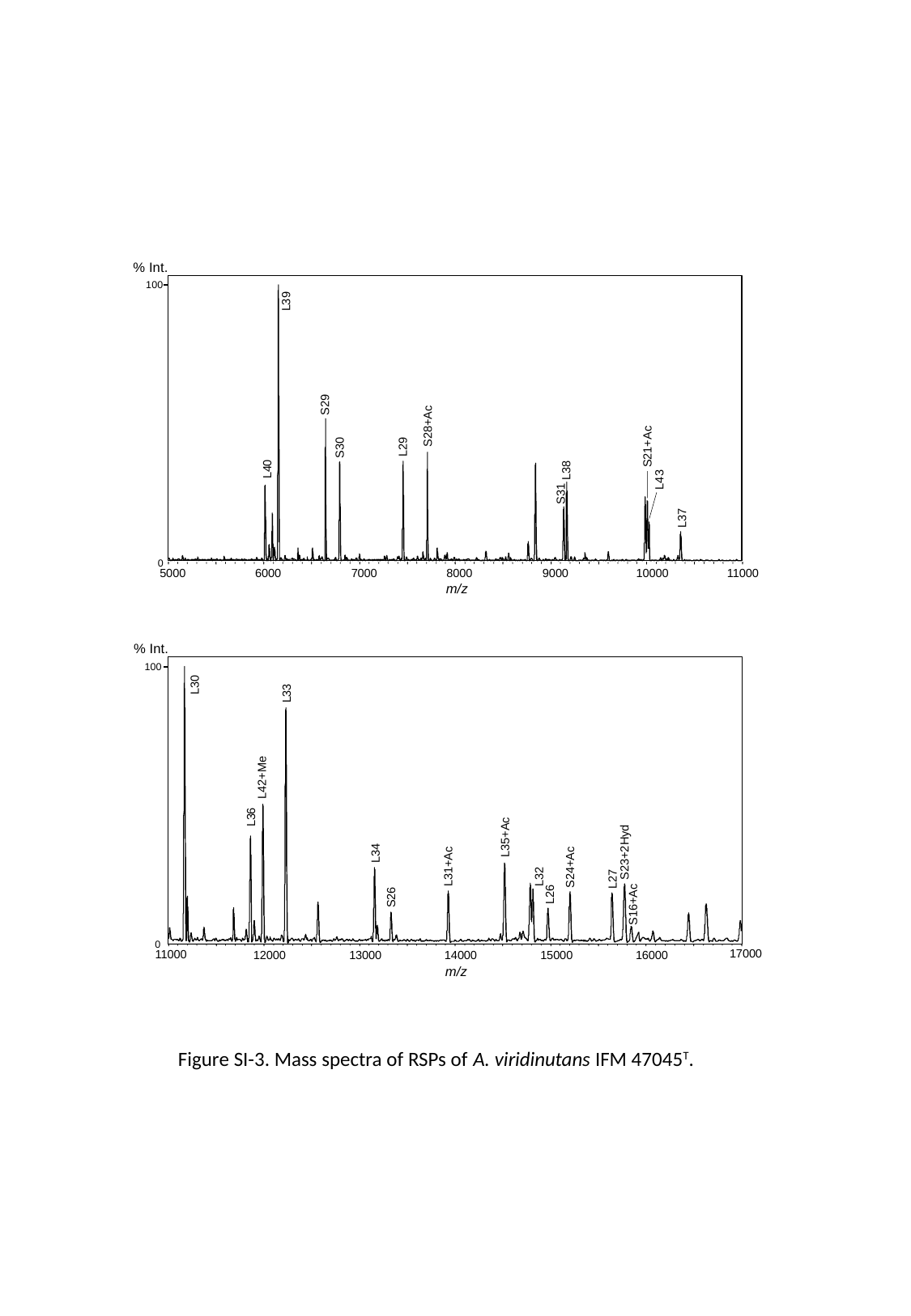

% Int.
100
L39
S29
S28+Ac
S30
L29
S21+Ac
L40
L38
L43
S31
L37
0
5000
6000
7000
8000
9000
10000
11000
m/z
% Int.
100
L30
L33
L42+Me
L36
L35+Ac
S23+2Hyd
L34
L31+Ac
S24+Ac
L32
L27
L26
S26
S16+Ac
0
17000
11000
12000
13000
14000
15000
16000
m/z
Figure SI-3. Mass spectra of RSPs of A. viridinutans IFM 47045T.

## Slide 4
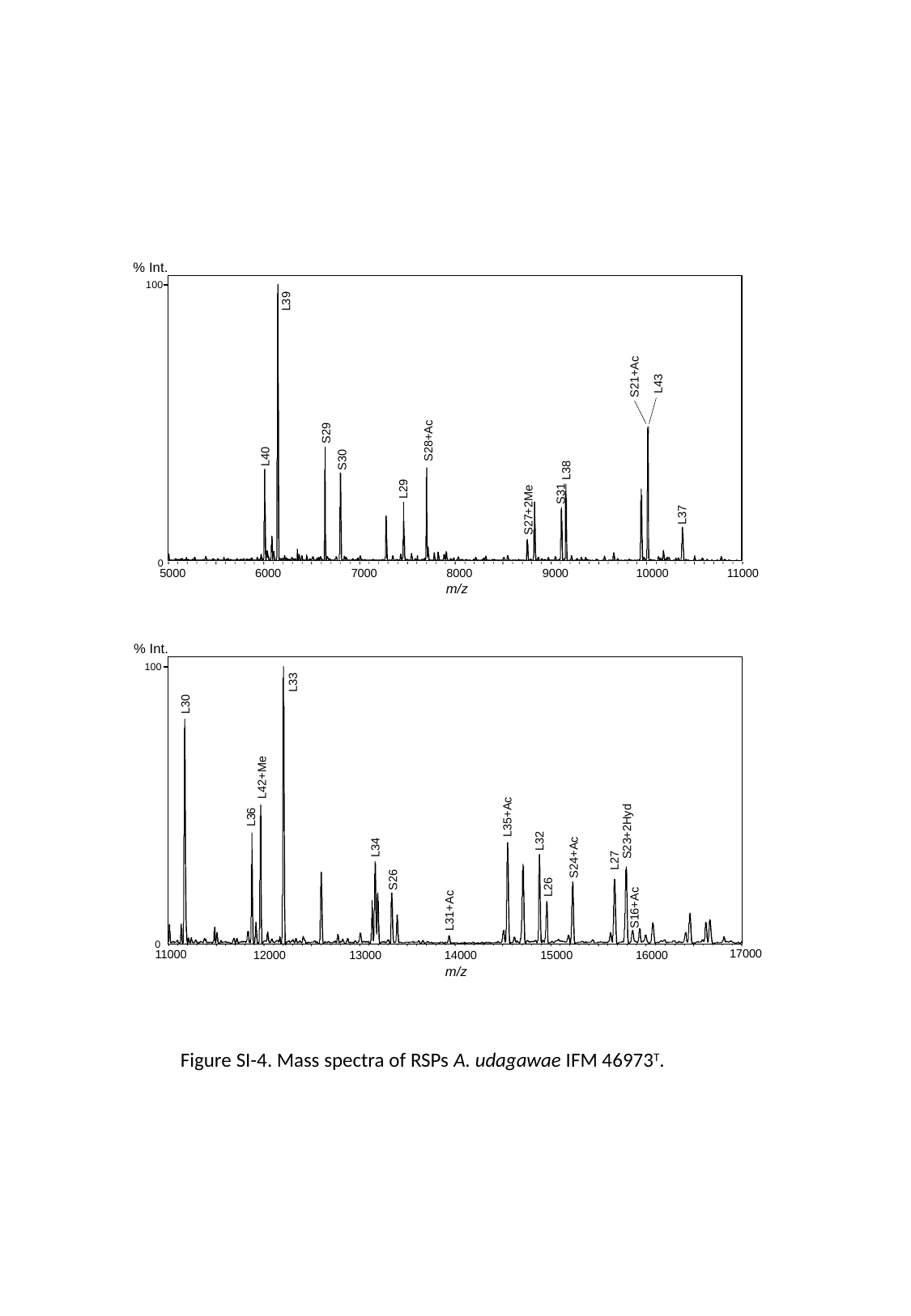

% Int.
100
L39
S21+Ac
L43
S29
S28+Ac
L40
S30
L38
L29
S31
S27+2Me
L37
0
5000
6000
7000
8000
9000
10000
11000
m/z
% Int.
100
L33
L30
L42+Me
L35+Ac
L36
S23+2Hyd
L32
L34
S24+Ac
L27
S26
L26
S16+Ac
L31+Ac
0
17000
11000
12000
13000
14000
15000
16000
m/z
Figure SI-4. Mass spectra of RSPs A. udagawae IFM 46973T.

## Slide 5
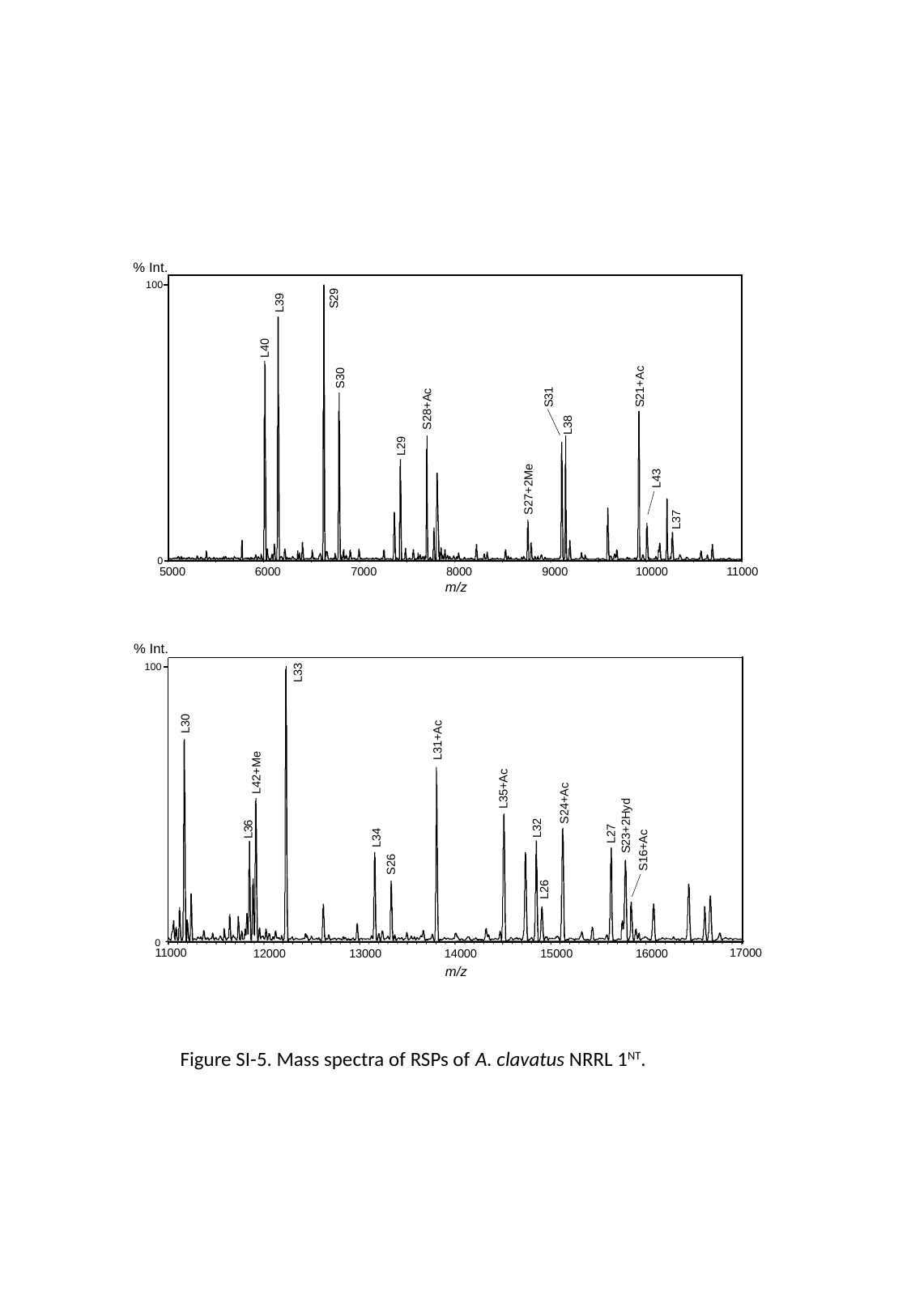

% Int.
100
S29
L39
L40
S30
S21+Ac
S31
S28+Ac
L38
L29
L43
S27+2Me
L37
0
5000
6000
7000
8000
9000
10000
11000
m/z
% Int.
100
L33
L30
L31+Ac
L42+Me
L35+Ac
S24+Ac
S23+2Hyd
L32
L36
L27
L34
S16+Ac
S26
L26
0
17000
11000
12000
13000
14000
15000
16000
m/z
Figure SI-5. Mass spectra of RSPs of A. clavatus NRRL 1NT.

## Slide 6
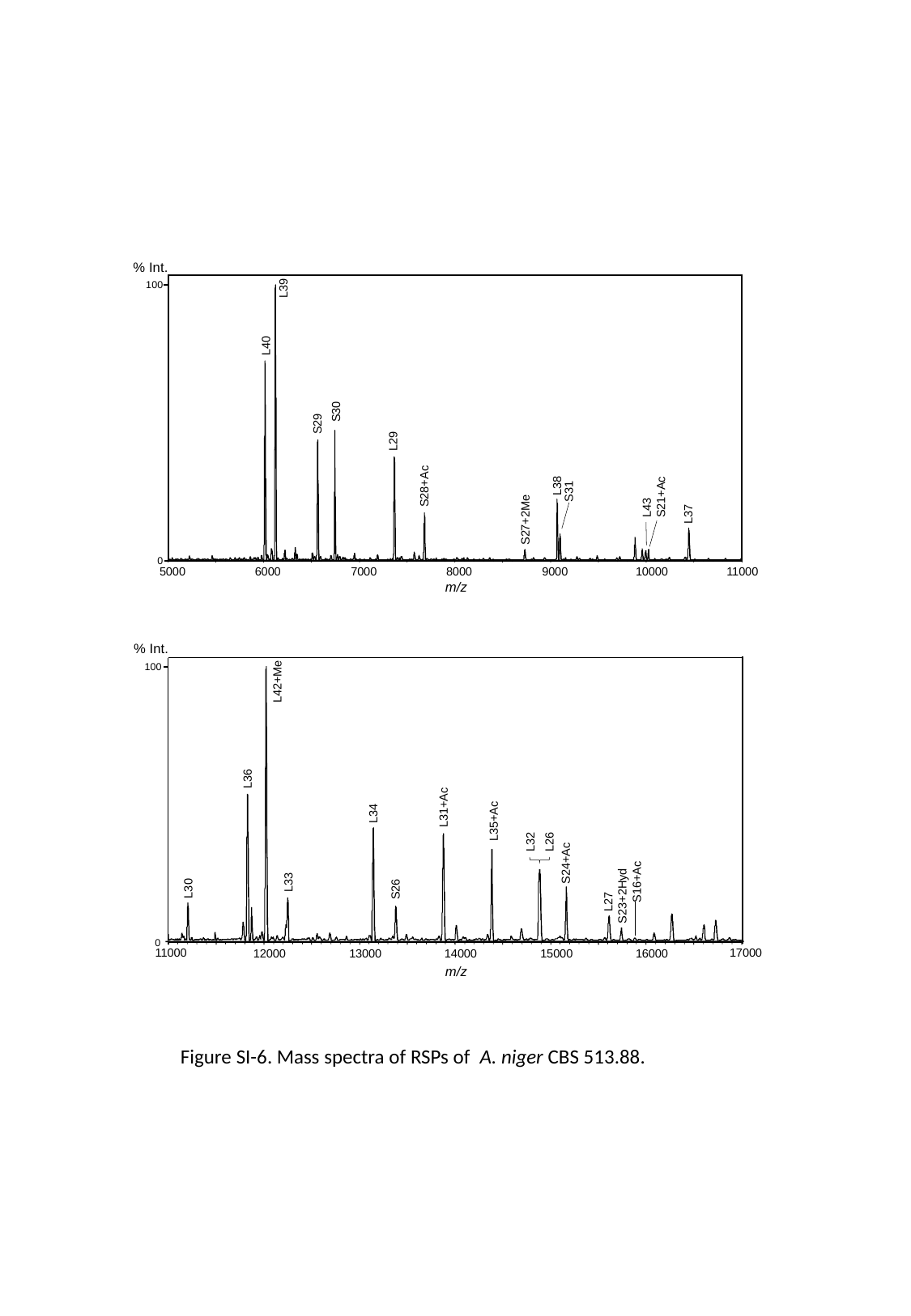

% Int.
100
L39
L40
S30
S29
L29
S28+Ac
L38
S31
S21+Ac
L43
L37
S27+2Me
0
5000
6000
7000
8000
9000
10000
11000
m/z
% Int.
100
L42+Me
L36
L31+Ac
L34
L35+Ac
L26
L32
S24+Ac
S16+Ac
L33
L30
S26
S23+2Hyd
L27
0
17000
11000
12000
13000
14000
15000
16000
m/z
Figure SI-6. Mass spectra of RSPs of A. niger CBS 513.88.

## Slide 7
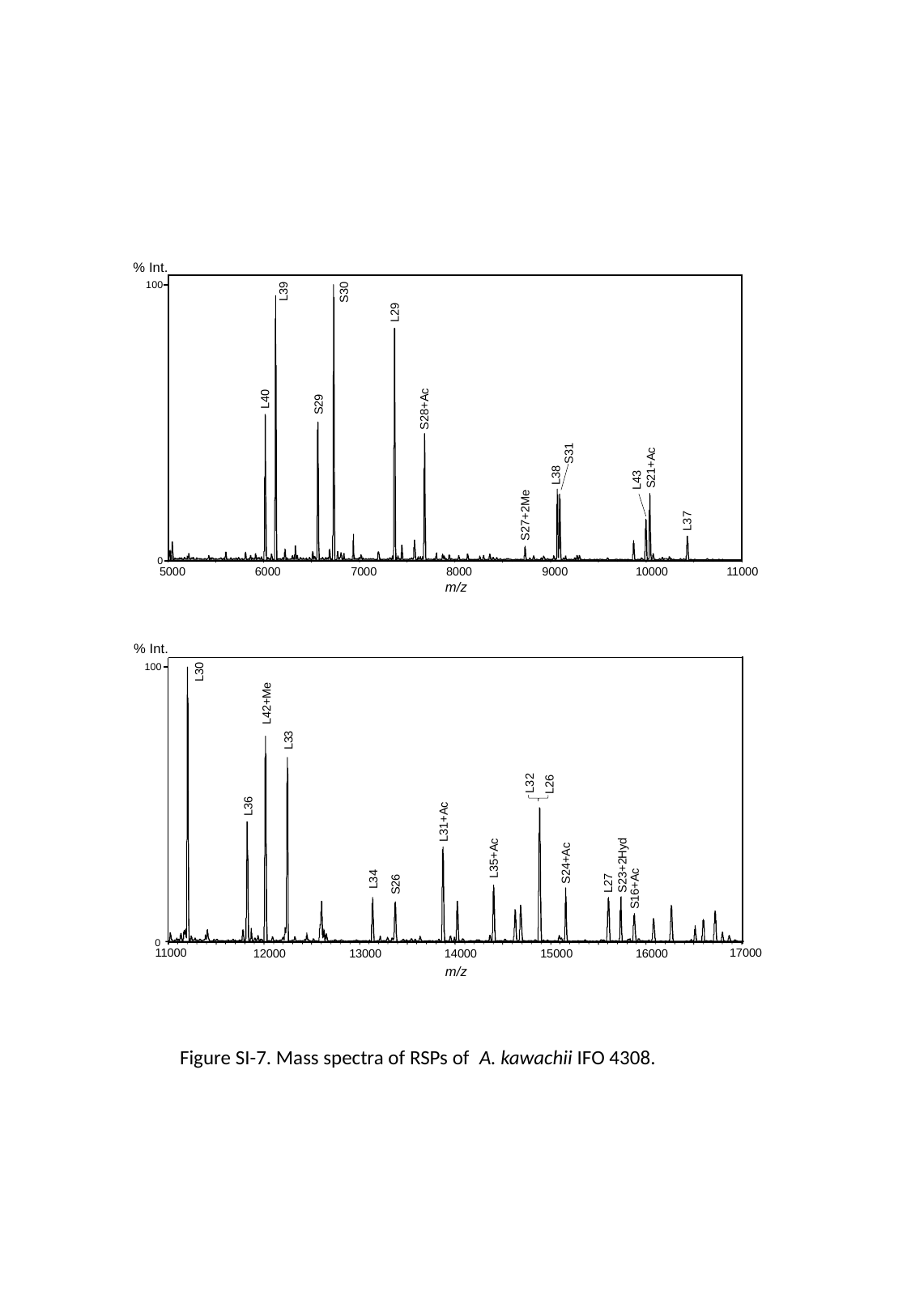

% Int.
100
L39
S30
L29
L40
S29
S28+Ac
S31
S21+Ac
L38
L43
S27+2Me
L37
0
5000
6000
7000
8000
9000
10000
11000
m/z
% Int.
100
L30
L42+Me
L33
L32
L26
L36
L31+Ac
L35+Ac
S24+Ac
S23+2Hyd
L34
L27
S26
S16+Ac
0
17000
11000
12000
13000
14000
15000
16000
m/z
Figure SI-7. Mass spectra of RSPs of A. kawachii IFO 4308.

## Slide 8
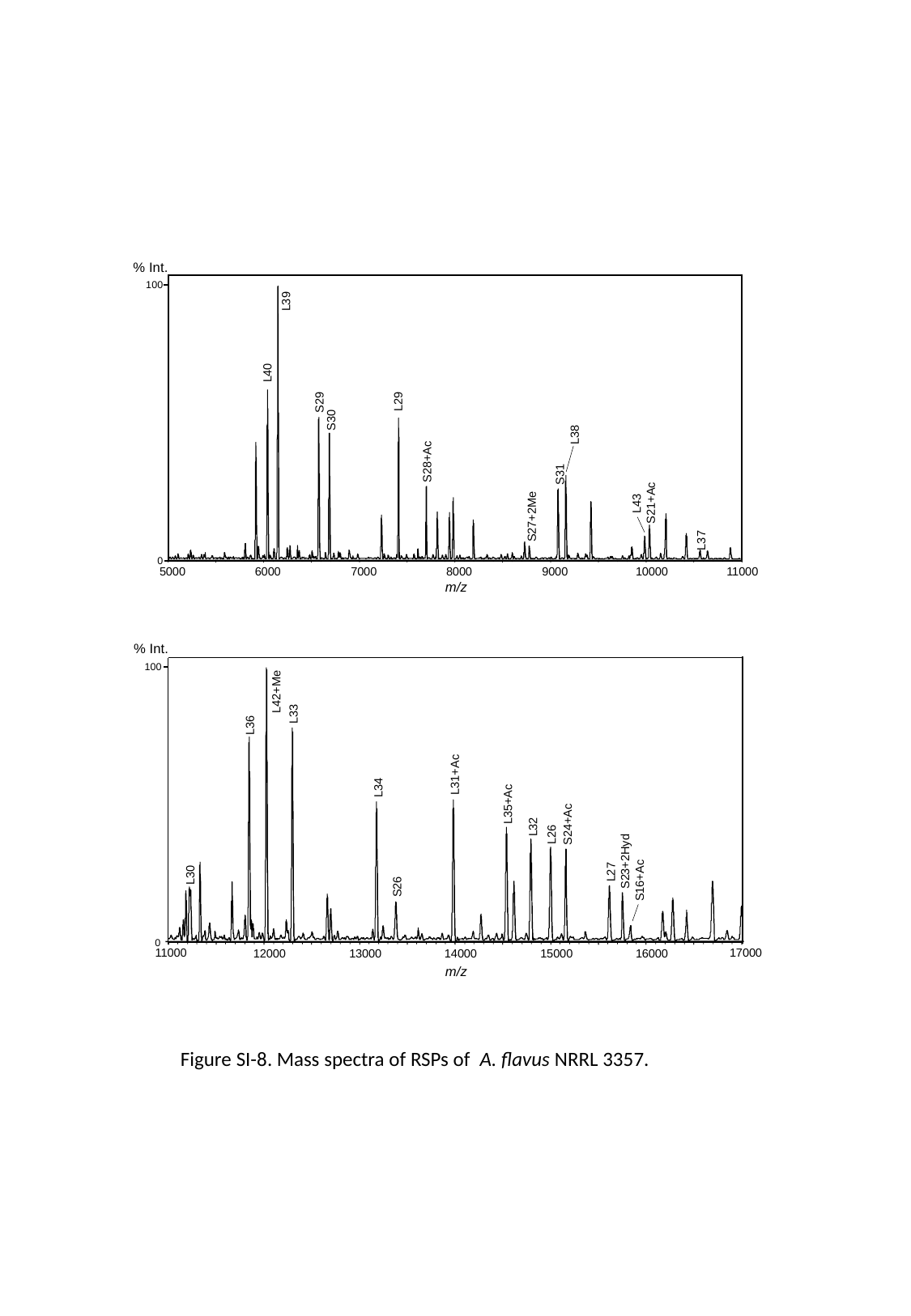

% Int.
100
L39
L40
L29
S29
S30
L38
S28+Ac
S31
L43
S21+Ac
S27+2Me
L37
0
5000
6000
7000
8000
9000
10000
11000
m/z
% Int.
100
L42+Me
L33
L36
L31+Ac
L34
L35+Ac
S24+Ac
L32
L26
S23+2Hyd
L27
L30
S16+Ac
S26
0
17000
11000
12000
13000
14000
15000
16000
m/z
Figure SI-8. Mass spectra of RSPs of A. flavus NRRL 3357.

## Slide 9
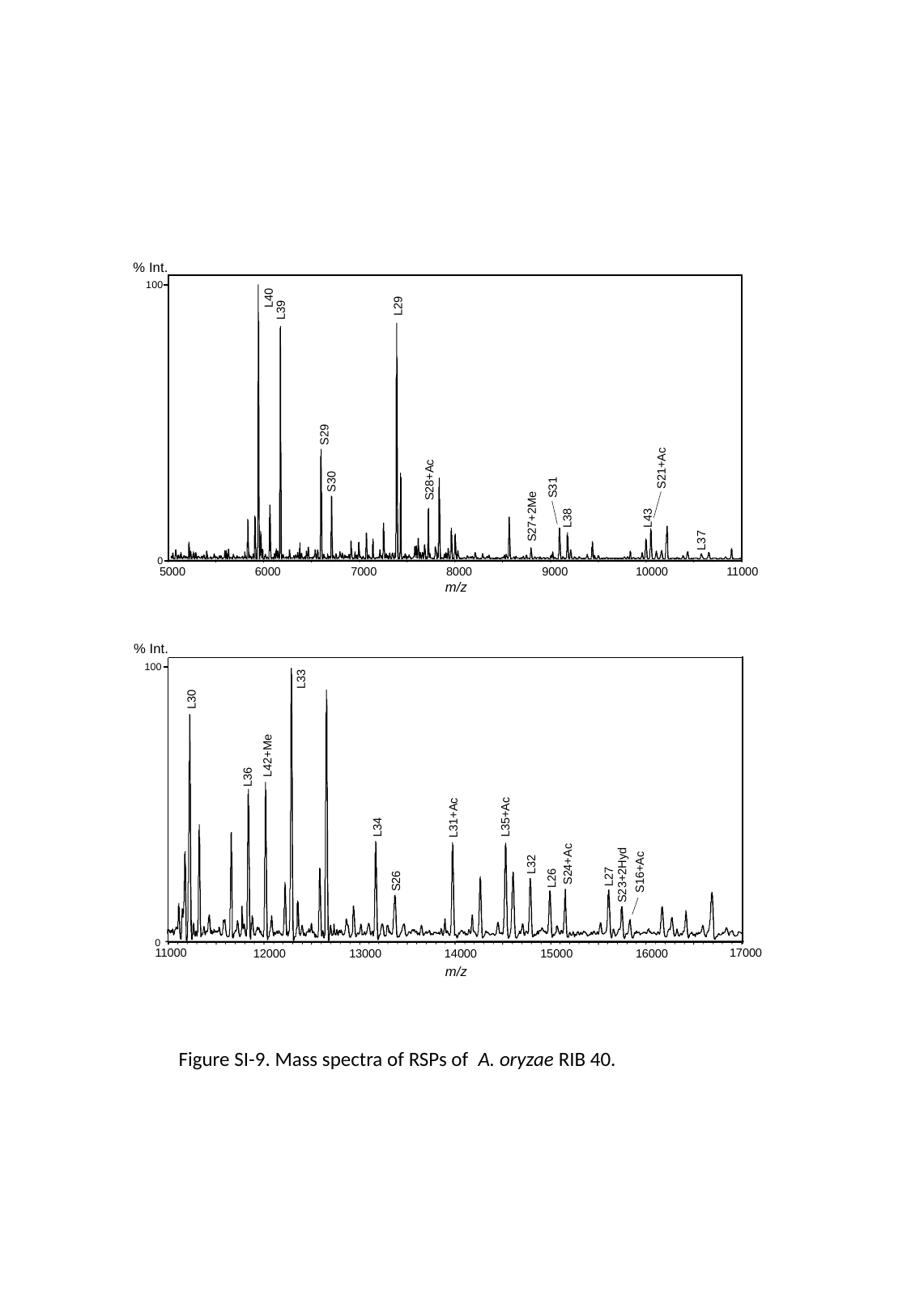

% Int.
100
L40
L29
L39
S29
S21+Ac
S28+Ac
S30
S31
S27+2Me
L43
L38
L37
0
5000
6000
7000
8000
9000
10000
11000
m/z
% Int.
100
L33
L30
L42+Me
L36
L35+Ac
L31+Ac
L34
S24+Ac
L32
S16+Ac
S23+2Hyd
L27
L26
S26
0
17000
11000
12000
13000
14000
15000
16000
m/z
Figure SI-9. Mass spectra of RSPs of A. oryzae RIB 40.

## Slide 10
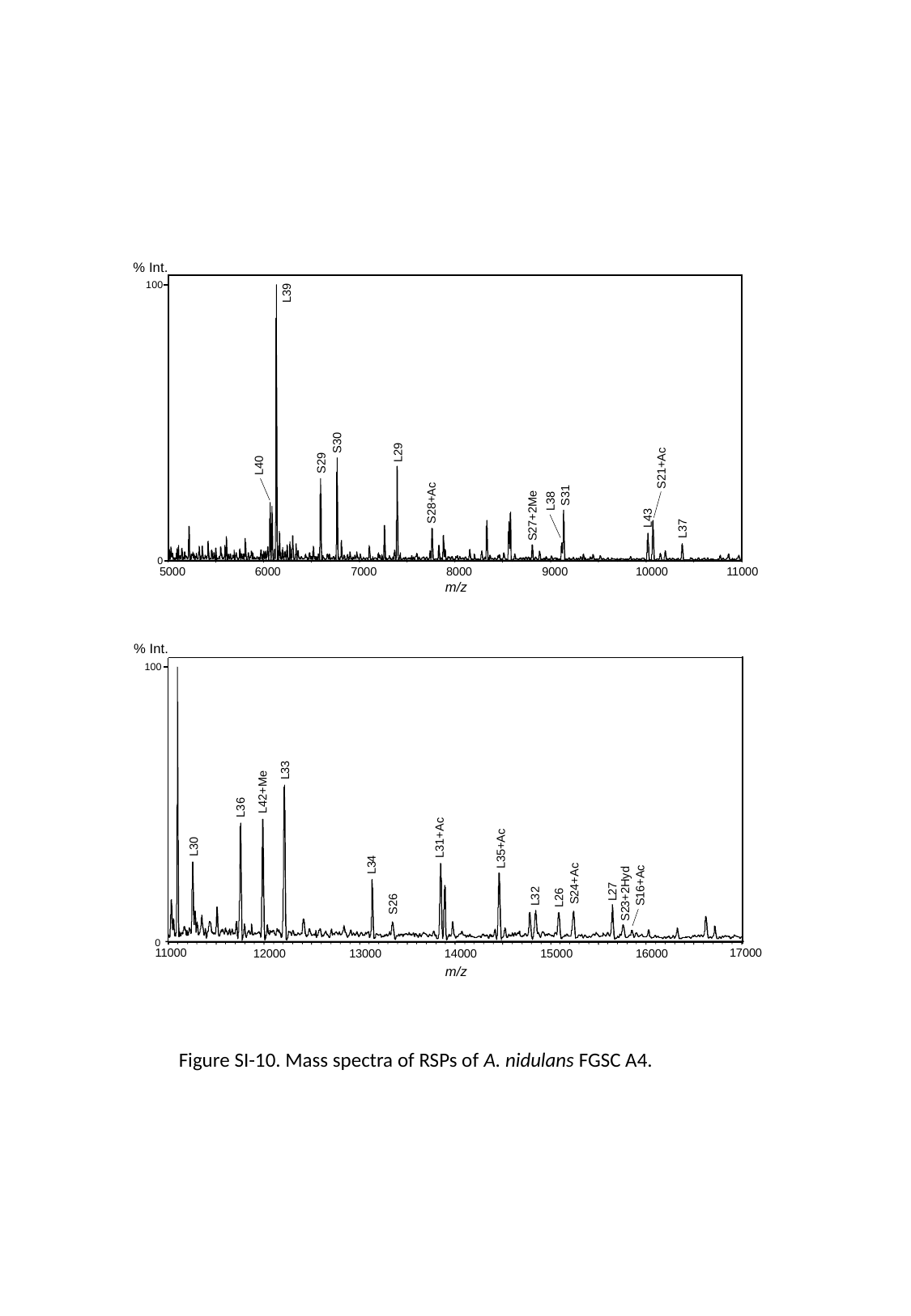

% Int.
100
L39
S30
L29
S29
L40
S21+Ac
S31
L38
S28+Ac
S27+2Me
L43
L37
0
5000
6000
7000
8000
9000
10000
11000
m/z
% Int.
100
L33
L42+Me
L36
L31+Ac
L30
L35+Ac
L34
S24+Ac
S16+Ac
L27
S23+2Hyd
L32
L26
S26
0
17000
11000
12000
13000
14000
15000
16000
m/z
Figure SI-10. Mass spectra of RSPs of A. nidulans FGSC A4.
