# Supplementary material for: Ribosomal subunit protein typing using matrix-assisted laser desorption ionization time-of-flight mass spectrometry (MALDI-TOF MS) for the identification and discrimination of Aspergillus species
Source: BMC Microbiol. 2017 Apr 26;17:100. doi: 10.1186/s12866-017-1009-3 (PMC5405522; doi:10.1186/s12866-017-1009-3)
Supplement: Supplementary file 4 — Post-translational modifications. Figure SI-11. Peak shift (+42 Da) of S24 with acetylation. (a) A. fumigatus A1163, (b) A. viridinutans IFM 47045T, (c) A. clavatus NRRL 1NT, (d) A. niger CBS 513.88, (e) A. flavus NRRL 3357, and (f) A. nidulans FGSC A4. Figure SI-12. Methylation of L42. (1) Peak shift of +14 Da from sequence mass in L42 of the Aspergillus species; (a) A. fumigatus A1163, (b) N. fischeri NRRL 181T, (c) A. lentulus IFM 54703T, (d) A. viridinutans IFM 47045T, (e) A. udagawae IFM 46973T, (f) A. clavatus NRRL 1NT, (g) A. niger CBS 513.88, (h) A. flavus NRRL 3357, and (i) A. nidulans FGSC A4. (2) Amino acid sequences around Lys-55. Figure SI-13. Dihydroxylation of S23. (1) Peak shift of +32 Da from sequence mass in L23 of Aspergillus species; (a) A. fumigatus A1163, (b) N. fischeri NRRL 181T, (c) A. niger CBS 513.88, (d) A. flavus NRRL 3357, and (e) A. nidulans FGSC A4. (2) Amino acid sequences around Pro-64. Figure SI-14. Peak shift (+28 Da) of S27 with dimethylation. (a) A. fumigatus A1163, (b) A. niger CBS 513.88, (c) A. flavus NRRL 3357, and (d) A. nidulans FGSC A4. (DOCX 187 kb) [file 12866_2017_1009_MOESM4_ESM.docx]

**
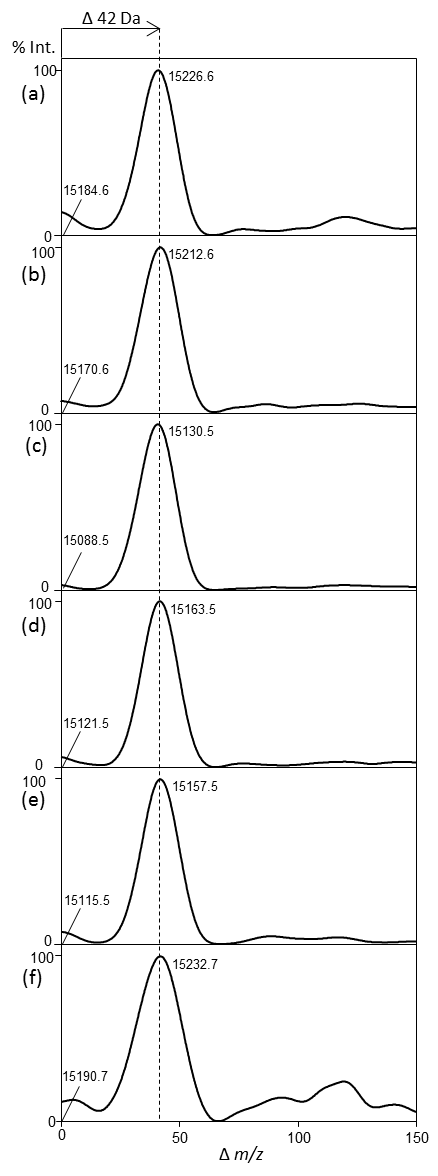
**

**Figure SI-11. Peak shift (+42 Da) of S24 with acetylation. (a) *A. fumigatus* A1163, (b) *A. viridinutans* IFM 47045^T^, (c) *A. clavatus* NRRL 1^NT^, (d) *A. niger* CBS 513.88, (e) *A. flavus* NRRL 3357, and (f) *A. nidulans* FGSC A4.**

Peak shift of +42 Da corresponds to acetylation. Such peak shifts from sequence mass (zero of x-axis) of S24 are commonly observed in all *Aspergillus* sample strains.


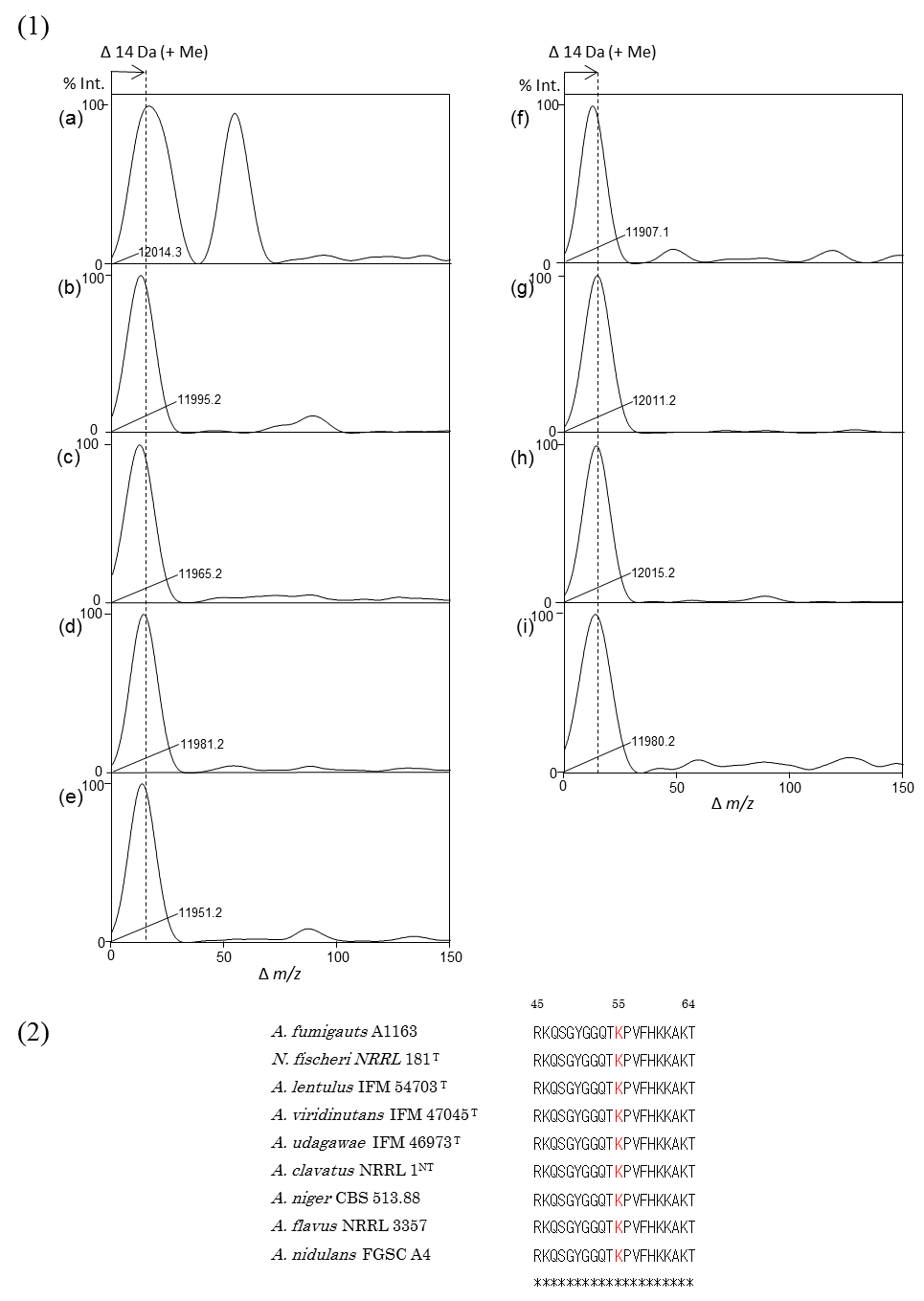


**Figure SI-12. Methylation of L42. (1) Peak shift of +14 Da from sequence mass in L42 of the *Aspergillus* species; (a) *A. fumigatus* A1163, (b) *N. fischeri* NRRL 181^T^, (c) *A. lentulus* IFM 54703^T^, (d) *A. viridinutans* IFM 47045^T^, (e) *A. udagawae* IFM 46973^T^, (f) *A. clavatus* NRRL 1^NT^, (g) *A. niger* CBS 513.88, (h) *A. flavus* NRRL 3357, and (i) *A. nidulans* FGSC A4. (2) Amino acid sequences around Lys-55.**

Peak shifts of +14 Da corresponding to methylation are commonly observed in the *Aspergillus* species. Methylation of L42 at Lys-55 is evolutionally conserved among eukaryotes [ref]. Amino acid sequences around Lys-55 in the *Aspergillus* species are the same among *Aspergillus* species. Therefore, methylation is supposed to occur commonly in these strains.

[ref] A. Shirai, M. Sadaie, K. Shinmyozu, J. Nakayama: **Methylation of ribosomal protein L42 regulates ribosomal function and stress-adapted cell growth**. *J Biol Chem* 2010, **285**(29):22448–22460.


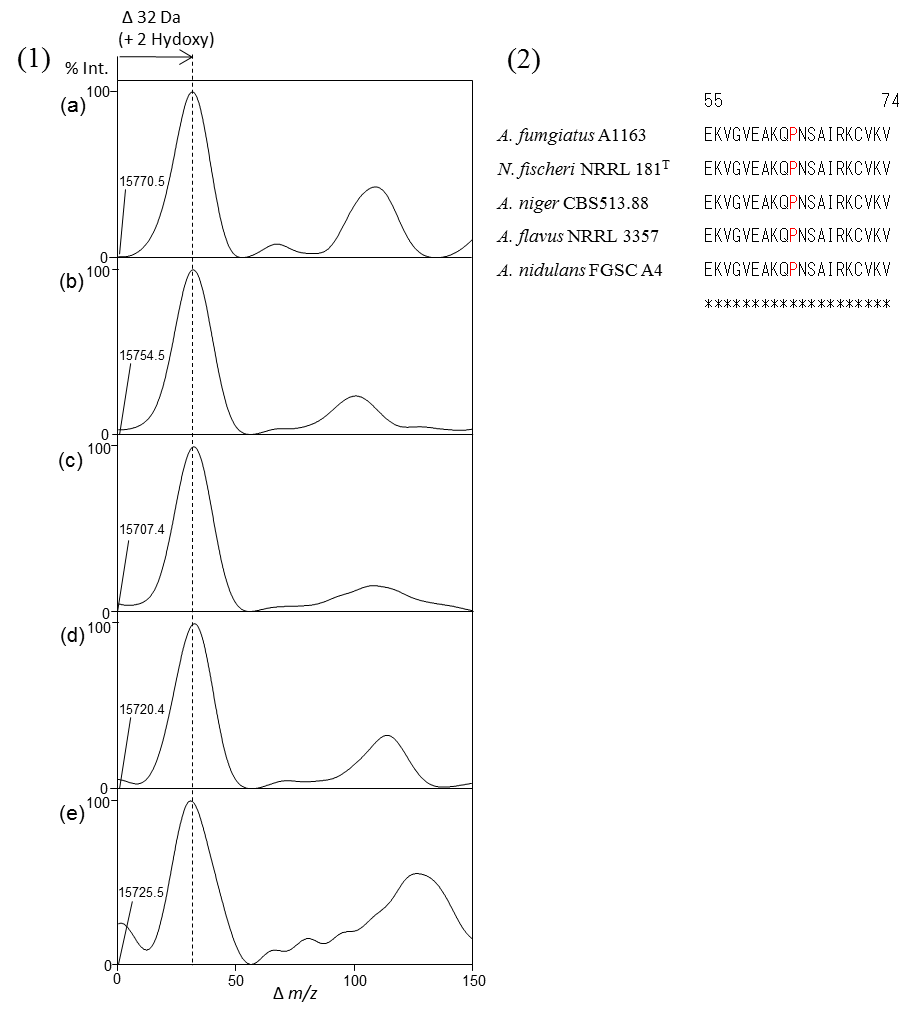


**Figure SI-13. Dihydroxylation of S23. (1) Peak shift of +32 Da from sequence mass in L23 of *Aspergillus* species; (a) *A. fumigatus* A1163, (b) *N. fischeri* NRRL 181^T^, (c) *A. niger* CBS 513.88, (d) *A. flavus* NRRL 3357, and (e) *A. nidulans* FGSC A4. (2) Amino acid sequences around Pro-64.**

Peak shifts of +32 Da corresponding to dihydroxylation are commonly observed in the *Aspergillus* species. Prolyl dihydroxylation of eukaryotic S23 is known as an evolutionally conserved modification [ref]. Amino acid sequences around Pro-64 are the same among the *Aspergillus* species. Therefore, dihydroxylation is supposed to occur commonly in these strains.

[ref] C. Loenarz, R. Sekirnik, A. Thalhammer, W. Ge, E. Spivakovsky, M. M. Mackeen, M. A. McDonough, M. E. Cockman, B. M. Kessler, P. J. Ratcliffe, A. Wolf, C. J. Schoeld: **Hydroxylation of the eukaryotic ribosomal decoding center affects translational accuracy.** *Proc Natl Acad Sci USA* 2014, **111**(11): 4019–4024.


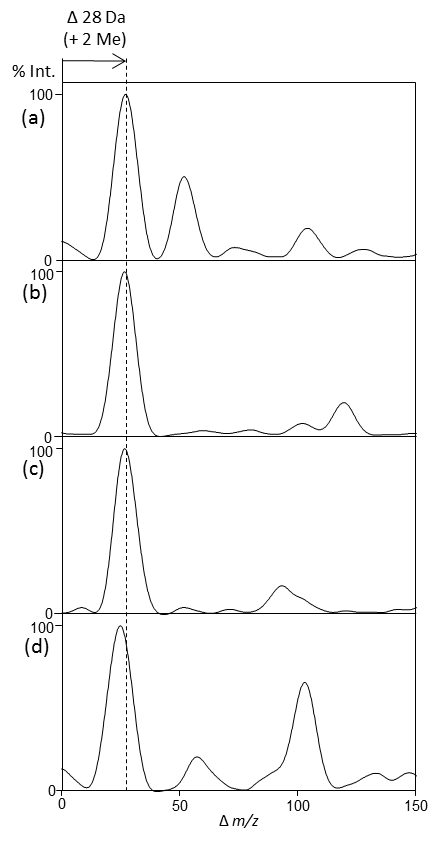


**Figure SI-14. Peak shift (+28 Da) of S27 with dimethylation. (a) *A. fumigatus* A1163, (b) *A. niger* CBS 513.88, (c) *A. flavus* NRRL 3357, and (d) *A. nidulans* FGSC A4.**

Peak shift of +28 Da corresponds to dimethylation. Such peak shifts from sequence mass (zero of x-axis) of S27 are commonly observed in all *Aspergillus* sample strains.
