# Supplementary material for: Ribosomal subunit protein typing using matrix-assisted laser desorption ionization time-of-flight mass spectrometry (MALDI-TOF MS) for the identification and discrimination of Aspergillus species
Source: BMC Microbiol. 2017 Apr 26;17:100. doi: 10.1186/s12866-017-1009-3 (PMC5405522; doi:10.1186/s12866-017-1009-3)
Supplement: Supplementary file 5 — Detailed experimental protocols. Detailed sample preparation procedures. Figure SI-16. Detailed construction procedure of the ribosomal protein biomarker list together with peak assignments. (PPTX 61 kb) [file 12866_2017_1009_MOESM5_ESM.pptx]

## Slide 1
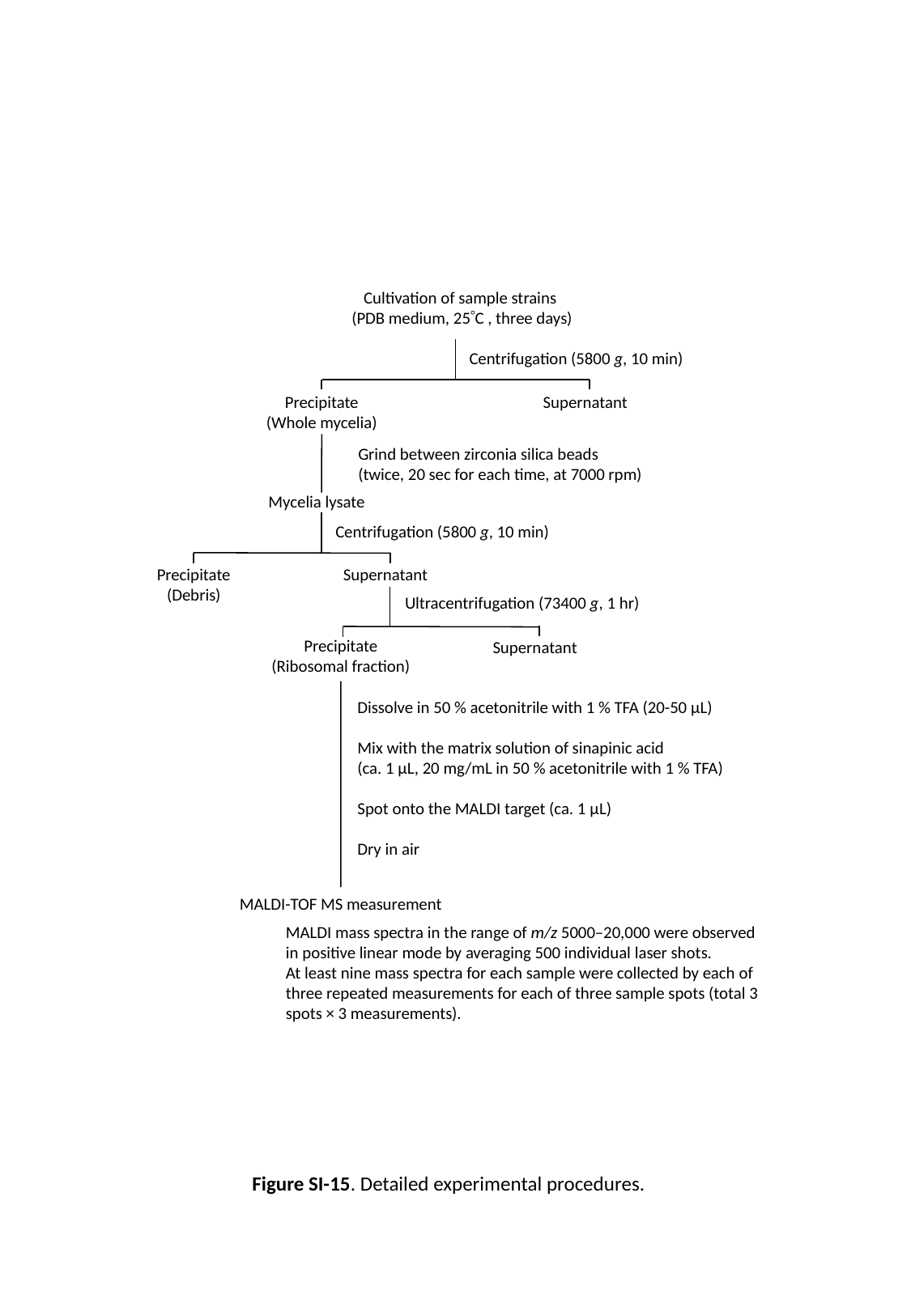

Cultivation of sample strains
 (PDB medium, 25°C , three days)
Centrifugation (5800 g, 10 min)
Precipitate
(Whole mycelia)
Supernatant
Grind between zirconia silica beads
(twice, 20 sec for each time, at 7000 rpm)
Mycelia lysate
Centrifugation (5800 g, 10 min)
Supernatant
Precipitate
(Debris)
Ultracentrifugation (73400 g, 1 hr)
Precipitate
(Ribosomal fraction)
Supernatant
Dissolve in 50 % acetonitrile with 1 % TFA (20-50 μL)
Mix with the matrix solution of sinapinic acid
(ca. 1 μL, 20 mg/mL in 50 % acetonitrile with 1 % TFA)
Spot onto the MALDI target (ca. 1 μL)
Dry in air
MALDI-TOF MS measurement
MALDI mass spectra in the range of m/z 5000–20,000 were observed in positive linear mode by averaging 500 individual laser shots.
At least nine mass spectra for each sample were collected by each of three repeated measurements for each of three sample spots (total 3 spots × 3 measurements).
Figure SI-15. Detailed experimental procedures.

## Slide 2
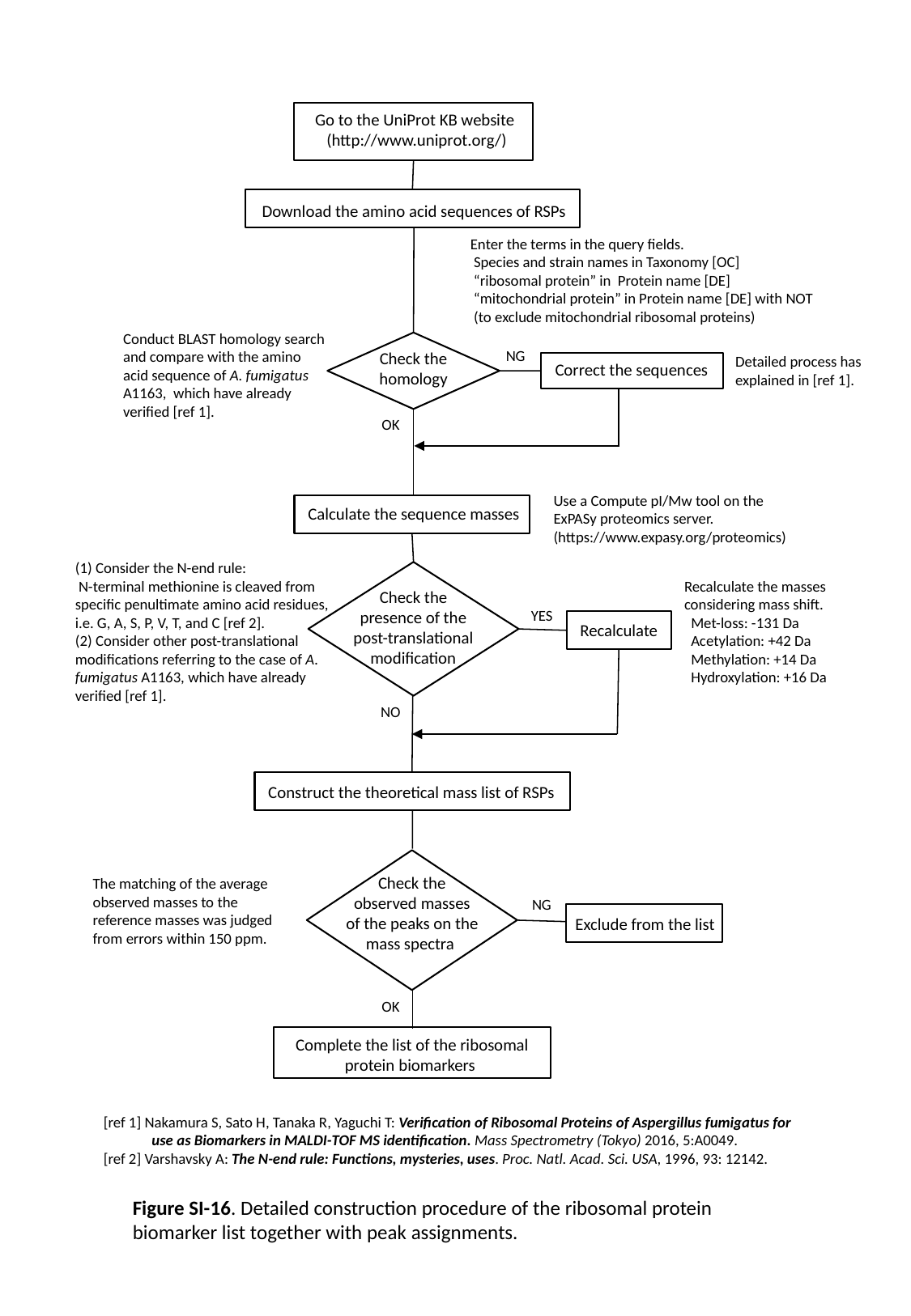

Go to the UniProt KB website
(http://www.uniprot.org/)
Download the amino acid sequences of RSPs
Enter the terms in the query fields.
 Species and strain names in Taxonomy [OC]
 “ribosomal protein” in Protein name [DE]
 “mitochondrial protein” in Protein name [DE] with NOT
 (to exclude mitochondrial ribosomal proteins)
Conduct BLAST homology search and compare with the amino acid sequence of A. fumigatus A1163, which have already verified [ref 1].
NG
Check the homology
Detailed process has explained in [ref 1].
Correct the sequences
OK
Use a Compute pI/Mw tool on the ExPASy proteomics server.
(https://www.expasy.org/proteomics)
Calculate the sequence masses
(1) Consider the N-end rule:
 N-terminal methionine is cleaved from specific penultimate amino acid residues, i.e. G, A, S, P, V, T, and C [ref 2].
(2) Consider other post-translational modifications referring to the case of A. fumigatus A1163, which have already verified [ref 1].
Recalculate the masses considering mass shift.
 Met-loss: -131 Da
 Acetylation: +42 Da
 Methylation: +14 Da
 Hydroxylation: +16 Da
Check the presence of the post-translational modification
YES
Recalculate
NO
Construct the theoretical mass list of RSPs
Check the observed masses of the peaks on the mass spectra
The matching of the average observed masses to the reference masses was judged from errors within 150 ppm.
NG
Exclude from the list
OK
Complete the list of the ribosomal protein biomarkers
[ref 1] Nakamura S, Sato H, Tanaka R, Yaguchi T: Verification of Ribosomal Proteins of Aspergillus fumigatus for use as Biomarkers in MALDI-TOF MS identification. Mass Spectrometry (Tokyo) 2016, 5:A0049.
[ref 2] Varshavsky A: The N-end rule: Functions, mysteries, uses. Proc. Natl. Acad. Sci. USA, 1996, 93: 12142.
Figure SI-16. Detailed construction procedure of the ribosomal protein biomarker list together with peak assignments.
